# Intracellular complement factor H protects neurons during CNS inflammation

**DOI:** 10.1038/s41586-026-10981-y

**Published:** 2026-09-02

**Authors:** Christina Mayer, Marcel S. Woo, Jana K. Sonner, Lars Binkle-Ladisch, Felix Fischbach, Patricia Sekol, Matthew D. Smith, Fernando Lucas-Ruiz, Darwin Manteufel, Kuno M.-J. Mattern, Lena Kristina Pfeffer, Yubing Guo, Bente Siebels, Albert Miguela, Vanessa Vieira, Nina Meurs, Simone Bauer, Sophia Schwarz, Sonia Wulf, Anne Katrin Mühlig, Jan Broder Engler, Marcus Conrad, Florence M. Bareyre, Thorsten Wiech, Susanne Krasemann, Markus Glatzel, Peter F. Zipfel, Tilo Freiwald, Peter A. Calabresi, Lucas Schirmer, Manuel A. Friese

**Affiliations:** 1 Institute of Neuroimmunology and Multiple Sclerosis (INIMS), University Medical Center Hamburg-Eppendorf, Hamburg, Germany; 2 Hamburg Center for Translational Immunology (HCTI), University Medical Center Hamburg-Eppendorf, Hamburg, Germany; 3 Division of Neuroimmunology, Department of Neurology, Medical Faculty Mannheim, Heidelberg University, Mannheim, Germany; 4 Departments of Neurology and Neuroscience, Johns Hopkins University School of Medicine, Baltimore, MD USA; 5 Group of Experimental Ophthalmology, Instituto Murciano de Investigación Biosanitaria Virgen de la Arrixaca (IMIB-Arrixaca) and Ophthalmology Department, Universidad de Murcia, Murcia, Spain; 6 III. Department of Medicine, University Medical Center Hamburg-Eppendorf, Hamburg, Germany; 7 Hamburg Center for Kidney Health (HCKH), University Medical Center Hamburg-Eppendorf, Hamburg, Germany; 8 Section Mass Spectrometry and Proteomics, University Medical Center Hamburg-Eppendorf, Hamburg, Germany; 9 Neurodegeneration and Neuroinflammation Group, Girona Biomedical Research Institute (IDIBG-CERCA), Salt, Spain; 10 Institute of Pathology, Nephropathology Section, University Medical Center Hamburg-Eppendorf, Hamburg, Germany; 11 University Children’s Hospital, University Medical Center Hamburg-Eppendorf, Hamburg, Germany; 12 University Children’s Research@Kinder-UKE, University Medical Center Hamburg-Eppendorf, Hamburg, Germany; 13 Institute of Metabolism and Cell Death, Molecular Targets and Therapeutics Center, Helmholtz Munich, Neuherberg, Germany; 14 Translational Redox Biology, Technical University of Munich (TUM), TUM Natural School of Sciences, Garching, Germany; 15 Institute of Clinical Neuroimmunology, University Hospital LMU Munich, Munich, Germany; 16 Institute of Neuropathology, University Medical Center Hamburg-Eppendorf, Hamburg, Germany; 17 Department of Infection Biology, Leibniz Institute for Natural Product Research and Infection Biology, Jena, Germany; 18 Mannheim Center for Translational Neuroscience, Medical Faculty Mannheim, Heidelberg University, Mannheim, Germany; 19 Mannheim Institute for Innate Immunoscience, Medical Faculty Mannheim, Heidelberg University, Mannheim, Germany; 20 Interdisciplinary Center for Neurosciences, Heidelberg University, Heidelberg, Germany

**Keywords:** Neuroimmunology, Multiple sclerosis, Neurodegeneration

## Abstract

Neurodegeneration is a major driver of disability in multiple sclerosis (MS), the most common chronic inflammatory disease of the central nervous system (CNS)^1^. Retinal ganglion cells (RGCs), a heterogeneous neuronal population in the eye, undergo degeneration in MS and provide a model to study neuronal subtype-specific resilience to inflammatory injury^2^. However, the neuron-intrinsic mechanisms underlying differential vulnerability remain unclear. Here we identify a neuroprotective role for intracellular complement factor H (CFH) in neurons. Using single-nucleus RNA-sequencing analysis of RGCs from donors with MS and control individuals, we found that *CFH* expression was strongly correlated with intrinsic resilience to RGC degeneration. Mechanistically, *CFH* was induced in retinal and other CNS neurons in response to inflammatory and oxidative stress, where it limited reactive oxygen species accumulation and lipid peroxidation. CFH localized to the endoplasmic reticulum, a major site of lipid peroxidation during neuronal ferroptosis. Its protective activity was dependent on its C-terminal SCR20 domain, was independent of CFH secretion and was preserved in the absence of complement component C3. These findings reveal a non-canonical intracellular function of CFH in neurons. Together, our results identify CFH as a key mediator of neuronal resilience across the CNS in mice and humans and provide mechanistic insight into inflammatory neurodegeneration with implications for MS therapy and neuroprotection more broadly.

## Abstract (web summary)

CFH is a key mediator of neuronal resilience across the central nervous system in mice and humans.

## Main

In MS, a disease that predominantly affects young adults, inflammation is followed by early and widespread neurodegeneration throughout the CNS^1^. The disease is thought to be driven by autoreactive T cells infiltrating the CNS and triggering chronic myeloid cell activation that sustains low-grade inflammation and gradually drives neurodegeneration across CNS regions, including the eye^3,4^. Although several immunomodulatory therapies target the peripheral immune system, none address the CNS-intrinsic inflammatory environment or reinforce stressed neurons against inflammation-induced degeneration, limiting their impact on progressive disability^5^.

In addition to motor and sensory impairments, visual disturbances are among the most common presenting symptoms in people with MS^4^. Retinal atrophy is an early feature of MS and can be detected even without a previous episode of optic neuritis^6^. Thus, acute and chronic low-grade CNS inflammation leads to substantial degeneration of RGCs, the output neurons of the retina of which the axons form the optic nerve.

RGCs are among the most diverse neuronal populations, with over 40 distinct subtypes described in mice^2^. This diversity has driven investigations into subtype-specific vulnerability, particularly in models such as optic nerve crush, where around 80% of RGCs degenerate within the first 2 weeks^2^. Notably, because humans evolved a fovea for high-resolution colour vision, RGCs in this region may face a trade-off of increased susceptibility to degeneration, as seen in age-related macular degeneration (AMD) and MS, emphasizing the need to study the mechanisms of retinal degeneration in humans^7^.

Recent work has begun to resolve the transcriptional heterogeneity of human RGCs^8–10^, but cell type identification remains inconsistent and datasets are dominated by midget RGCs, limiting analysis of rarer subtypes. Although MS substantially affects the retina, single-cell studies have focused largely on the brain^11–13^ and histopathological studies of the MS retina are sparse^14^, leaving a gap in our understanding of how MS-associated inflammation affects retinal neurons. Moreover, most single-cell studies describe molecular changes without translating them into clinical concepts or therapies. To address this, we performed single-nucleus sequencing analysis of RGCs from control and MS retinas to identify genes determining neuronal vulnerability to inflammation, then validated and mechanistically explored our findings to identify how neurons protect themselves against inflammation across the CNS.

## RGCs in health and MS

**Fig. 1:**
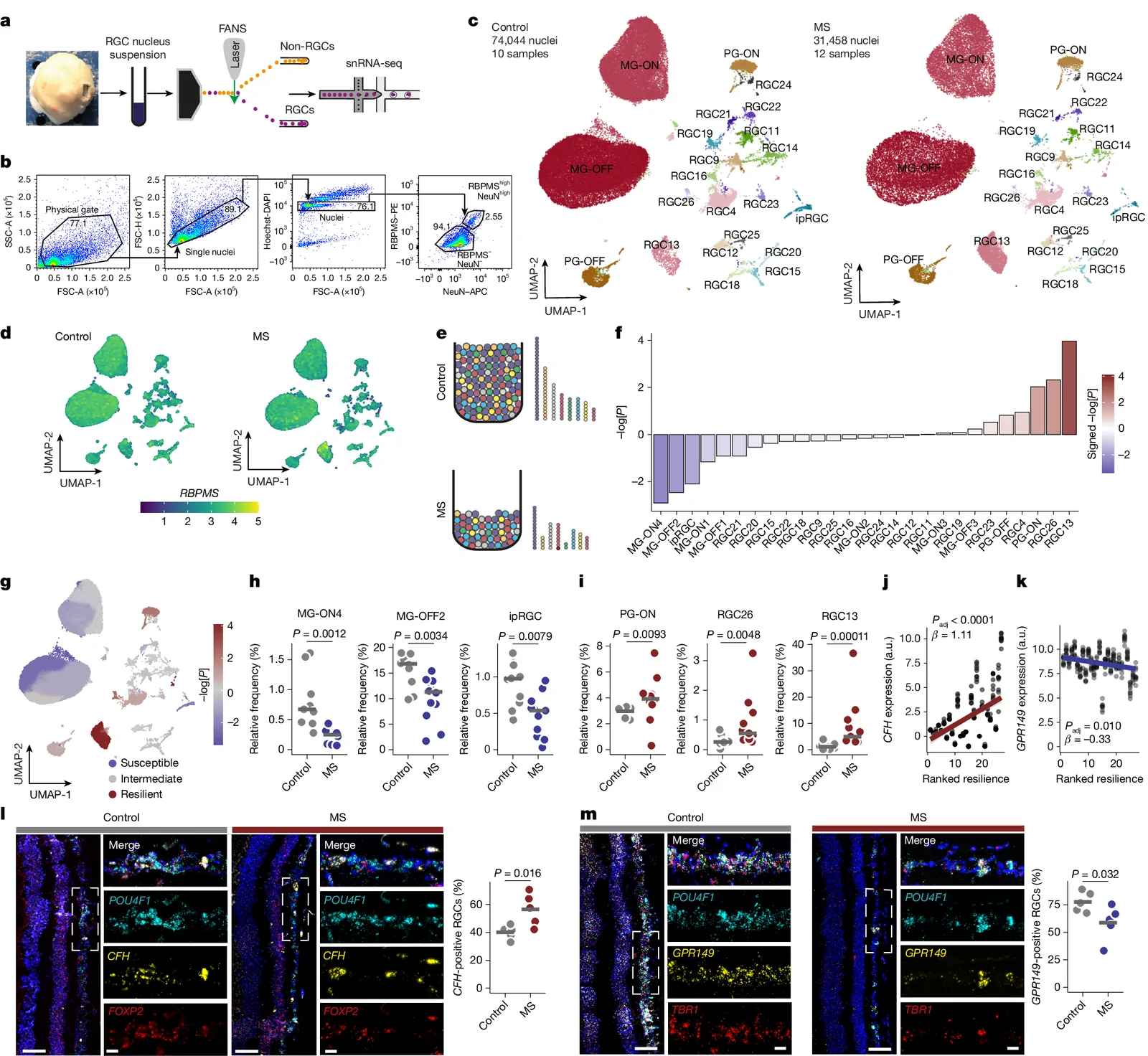
snRNA-seq analysis of human RGCs reveals subtype-specific vulnerability in MS. **a**, The snRNA-seq workflow. **b**, The FANS strategy to isolate RGC nuclei from human post-mortem retinas using NeuN and RBPMS antibodies. **c**, UMAP of 74,044 RGC nuclei from controls (*n* = 10 retinas, 10 donors) and 31,458 RGC nuclei from MS (*n* = 12 retinas, 10 donors). **d**, Validation of *RBPMS* expression. **e**, The distribution of RGC subtypes in control and MS samples. **f**, RGC subtype survival ranked by the –log[*P*] relative frequency (MS versus control). **g**, Integrated UMAP highlighting susceptible (blue) and resilient (red) RGC subtypes. **h**,**i**, The relative frequencies of the most susceptible (ON4 midget (MG-ON4), OFF2 midget (MG-OFF2) and ipRGCs) RGC (**h**) and resilient (ON parasol (PG-ON), RGC26 and RGC13) RGC (**i**) subtypes in controls (*n* = 10 retinas, 10 donors) and MS (*n* = 12 retinas, 10 donors). **j**,**k**, Linear regression of subtype- and sample-specific pseudobulk gene expression (arbitrary units, a.u.) against RGC subtype resilience rank identified *CFH* as positively (**j**) and *GPR149* as negatively (**k**) associated with resilience. RGC subtypes were ranked from most susceptible (0) to most resilient (26). Datapoints represent subtype- and sample-specific pseudobulk expression; the shaded areas indicate 95% confidence intervals; *β* estimates and false-discovery rate (FDR)-adjusted *P* (*P*_adj_) values are shown. **l**,**m**, Validation by smFISH in human retinas showing proportions of *CFH*-positive RGCs (POU4F1^+^) (controls: *n* = 18 slides, 5 donors; MS: *n* = 14 slides, 5 donors) (**l**) and *GPR149*-positive RGCs (*n* = 16 slides, 5 donors per group) (**m**). *GPR149* was co-stained with *TBR1*, and *CFH* was co-stained with FOXP2. Scale bars, 100 µm (overview) and 25 µm (magnified images). The datapoints represent biological replicates; the bars indicate the mean. Statistical analysis was performed using two-tailed Mann–Whitney *U*-tests after Grubbs’ outlier detection (**h** and **i**) or two-tailed Mann–Whitney *U*-tests (**l** and **m**). Source data

**Extended Data Fig. 1:**
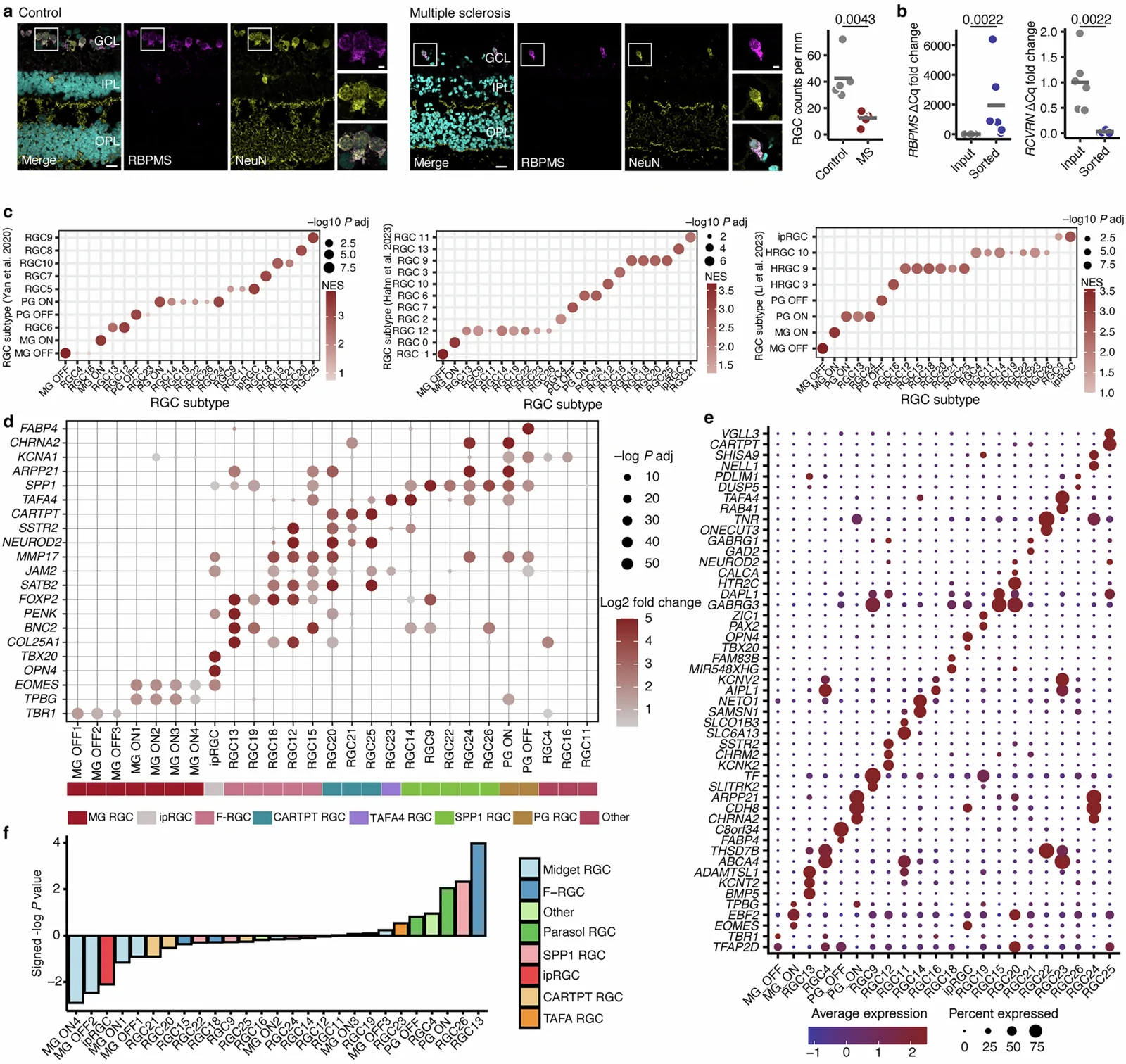
RGC snRNA-seq cluster annotation. **a**, Representative immunohistochemistry with antibodies against RBPMS and NeuN in retinas from a control individual and an individual with MS. Scale bars, 20 µm (overview) and 5 µm (magnified images). Corresponding quantification of retinal ganglion cells (RGCs), identified as NeuN + /RBPMS+ cells, in controls (*n* = 5) and MS (*n* = 6). **b**, qRT-PCR showing enrichment of *RBPMS* transcripts and deenrichment of *RCVRN* transcripts in human retinal nuclei sorted for positivity of both NeuN and RBPMS (*n* = 6), compared with input (*n* = 6), presented as ΔCq fold change relative to input. **c**, Dot plot showing gene set enrichment analysis of the top cluster marker genes from the indicated reference data sets^8–10^ against the ranked list of cluster marker genes identified in this study. Dot colour indicates normalized enrichment score (NES), and dot size the −log10 FDR-adjusted *P* value. **d**, Dot plot of selected marker genes across RGC subtypes. Dot colour indicates log2 fold change of differential gene expression; dot size represents −log10 adjusted *P* value. **e**, Dot plot showing top marker genes per each RGC subtype. Dot colour reflects mean expression per cell, and dot size the percentage of cells expressing each gene. **f**, Relative survival of RGC subtypes, colour-coded by family and ranked by relative frequency ratio (MS vs. control). Individual data points represent biological replicates; bars indicate the mean. Statistical comparisons used unpaired two-sided Mann-Whitney *U* test (**a,b**). Source data

To capture the heterogeneity of RGCs, which constitute only around 1% of retinal cells, we developed a fluorescence-activated nucleus sorting (FANS) protocol to extract their nuclei from human post-mortem retina^8^ (Fig. 1a). We used NeuN as a pan-neuronal marker and also labelled RGCs with an antibody against RNA-binding protein with multiple splicing (RBPMS), the principal RGC marker^15^, revealing the expected severe RGC loss in individuals with MS (Extended Data Fig. 1a). We sorted NeuN^high^RBPMS^high^ nuclei (Fig. 1b) and confirmed RGC enrichment using quantitative PCR with reverse transcription (RT–qPCR), observing a strong increase in *RBPMS* transcripts and depletion of recoverin (*RCVRN*), a photoreceptor marker (Extended Data Fig. 1b).

For single-nucleus RNA-sequencing (snRNA-seq) analysis, we next focused on the macula region, where MS-associated RGC degeneration is most severe^16^. Macula tissue was obtained from ten donors without retinal pathology (40% female; mean age, 55.8 years) and ten donors with MS (60% female; mean age, 58.6 years; Supplementary Table 1). After quality control, including removal of doublets, low-quality nuclei, nuclei with over 5% mitochondrial gene content (Supplementary Fig. 1a) and non-RGC nuclei, we retained 105,502 high-quality RGC transcriptomes (Fig. 1c and Supplementary Fig. 1c,d), with a mean of 4,567 genes detected per nucleus (Supplementary Fig. 1d).

RGC identity was confirmed by *RBPMS* expression (Fig. 1d). Small or inconsistent clusters were merged with their nearest neighbour (Supplementary Fig. 1e), and the resulting clusters were annotated using gene set enrichment against three published human RGC datasets^8–10^ (Extended Data Fig. 1c) and known RGC markers^2,8^ (Extended Data Fig. 1d,e). Consistent with previous studies^8–10^, midget RGCs were the largest population, followed by parasol RGCs (PGs), which subdivided into PG-ON and PG-OFF groups based on the transcriptional profile. The third most frequent type comprised *FOXP2*-expressing F-RGCs. RGC20, RGC21 and RGC25 expressed markers of ON–OFF direction-selective RGCs^2^ (*SATB2*, *CARTPT*, *NEUROD2* and *MMP17*), while RGC9, RGC14, RGC22, RGC24, RGC26 and the PGs expressed *SPP1*, a marker for alpha RGCs in mice^2^. We also identified *OPN4-*expressing intrinsically photosensitive RGCs (ipRGCs) and *TAFA4*-expressing populations^2^ (Extended Data Fig. 1d,e). Thus, our dataset resolved all major RGC subtypes with high concordance to existing human datasets in both control and MS samples.

## RGC subtype-specific vulnerability

To assess whether RGC subtypes differ in vulnerability to inflammation, we calculated the relative frequency of each cluster within the total RGC population in MS and control samples (Fig. 1e), revealing significant differences in survival (Fig. 1f,g). Midget RGCs and ipRGCs showed the greatest loss, whereas the PG-ON, RGC26 and RGC13 subtypes were relatively preserved (Fig. 1h,i and Extended Data Fig. 1f).

**Extended Data Fig. 2:**
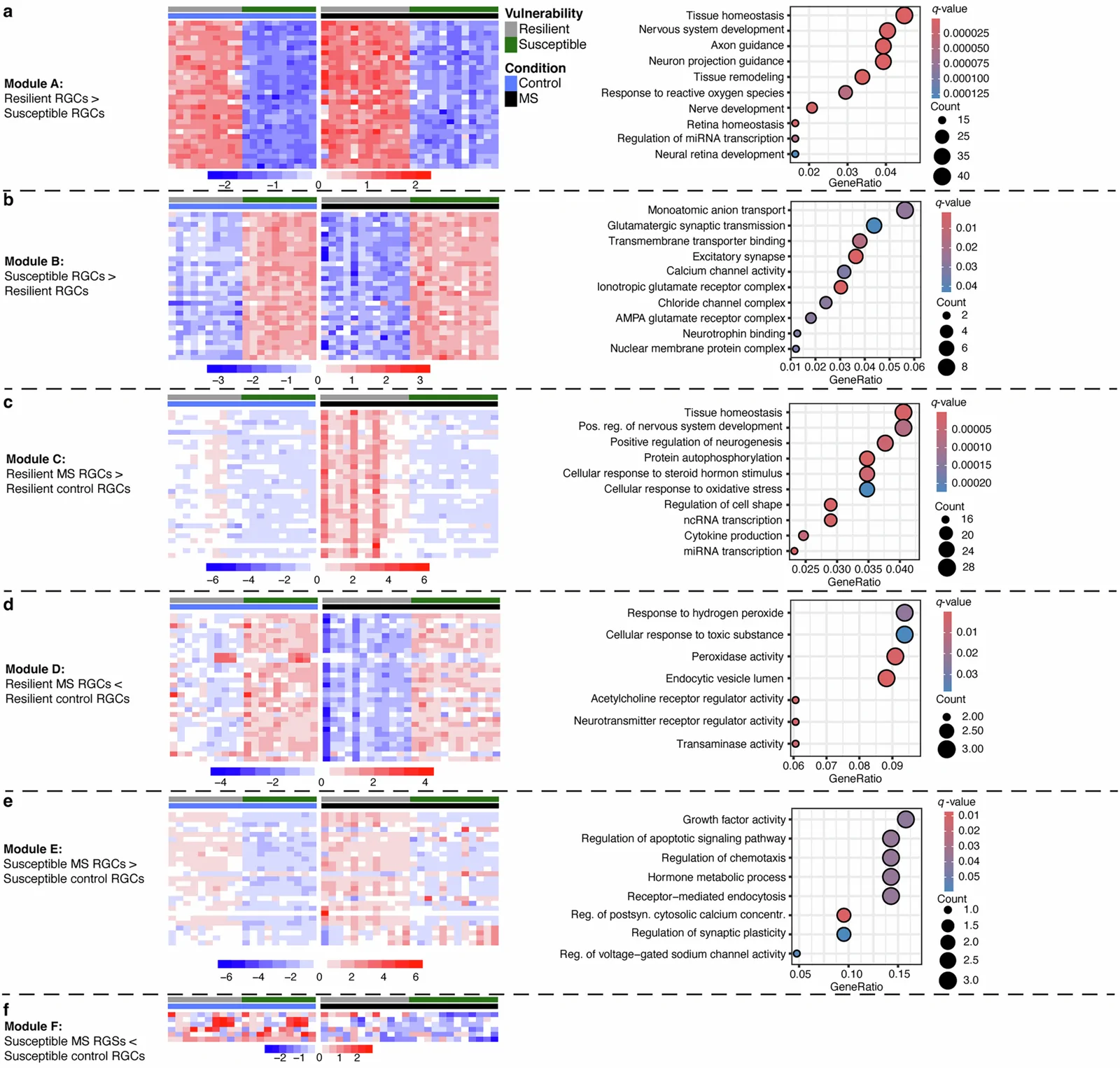
Molecular signatures of resilient and susceptible RGCs. **a**, Heatmap of the top 30 genes with higher expression in pseudobulked resilient retinal ganglion cells (RGCs) than in susceptible RGCs (Module A), with corresponding enriched gene ontology (GO) terms. **b**, Heatmap of the top 30 genes with higher expression in pseudobulked susceptible RGCs than in resilient RGCs (Module B), with corresponding enriched GO terms. **c**, Heatmap of the top 30 genes upregulated in pseudobulked resilient RGCs during MS (Module C), with corresponding enriched GO terms. **d**, Heatmap of the top 30 genes downregulated in pseudobulked resilient RGCs during MS, with corresponding enriched GO terms (Module D). **e**, Heatmap of the top 30 genes upregulated in pseudobulked susceptible RGCs during MS (Module E), with corresponding enriched GO terms. **f**, Six genes were downregulated in pseudobulked susceptible RGCs during MS (Module F). Colour scale shows row scaled expression. Source data

**Extended Data Fig. 3:**
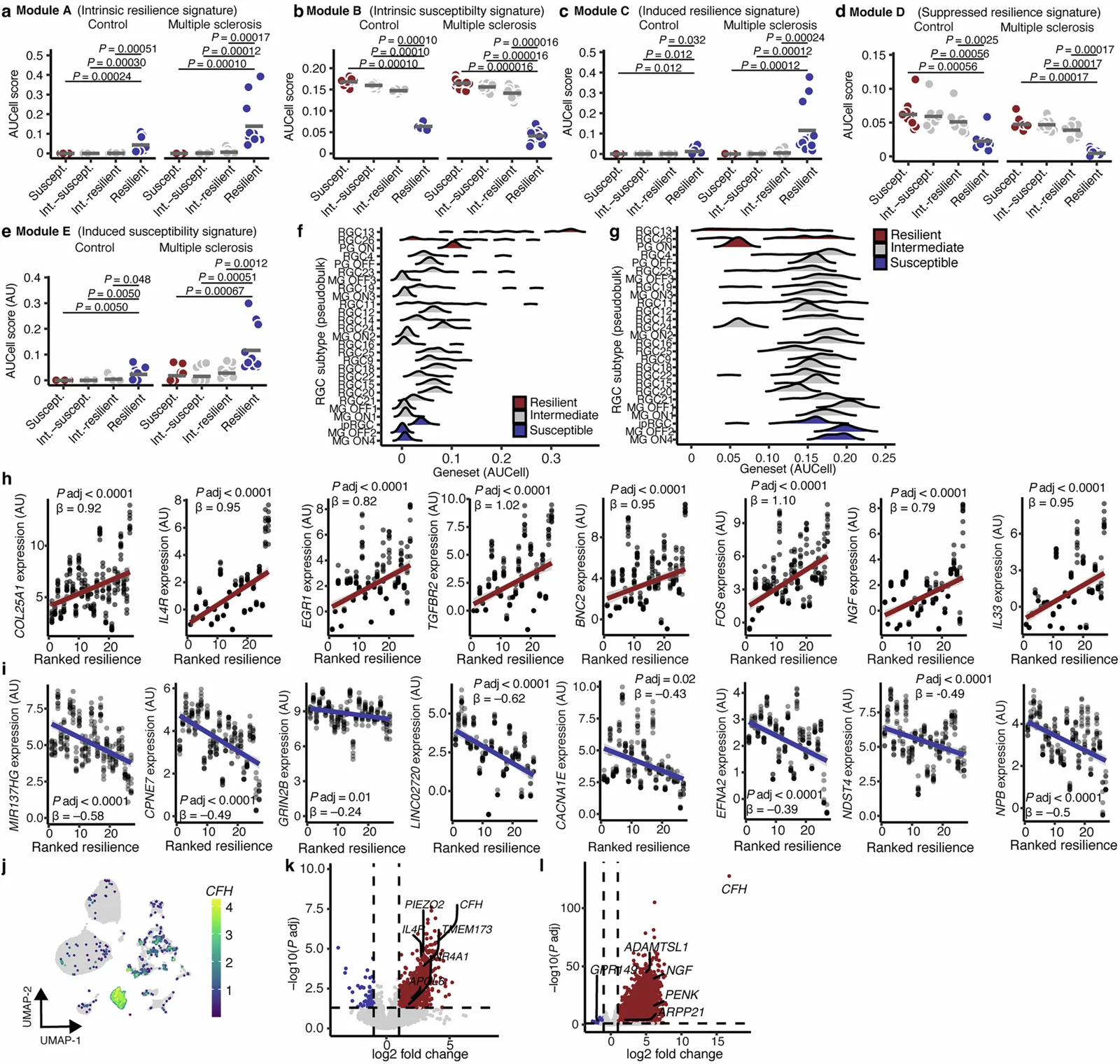
Distinct transcriptional signatures define resilient and susceptible RGCs. **a–e**, AUCell analysis of pseudobulked expression data using gene signatures derived from expression modules: resilience signature (top 200 Module A genes enriched in resilient RGCs vs. susceptible RGCs) (**a**); susceptibility signature (top 200 Module B genes enriched in susceptible vs. resilient RGCs) (**b**); induced resilience signature (top 200 Module C genes upregulated selectively in resilient RGCs during MS) (**c**); suppressed resilience signature (all 91 Module D genes downregulated selectively in resilient RGCs during MS) (**d**); and induced susceptibility signature (all 27 Module E genes upregulated selectively in susceptible RGCs during MS) (**e**). **f,g**, AUCell scores of the resilience (top 200 genes from Module A) (**f**) and susceptibility signatures (top 200 genes from Module B) (**g**) across RGC subtypes ranked from most susceptible (bottom) to most resilient (top). **h,i**, Linear regression of subtype- and sample-specific pseudobulk expression with RGC resilience. Subtypes were ranked from most susceptible (0) to most resilient (26) using signed −log(*P*) values from relative frequency differences between control (*n* = 10 retinas, 10 donors) and MS (*n* = 12 retinas, 10 donors). Eight genes showing strong positive (**h**) or negative (**i**) associations with resilience are shown. β estimates and FDR-adjusted *P* values are indicated; individual points represent subtype- and sample-specific pseudobulk expression from individual control and MS retinas with shaded 95% confidence intervals. **j**, UMAP showing *CFH* expression in the human RGC data set. **k**, Volcano plot of differential gene expression between control (*n* = 10 retinas, 10 donors) and MS (*n* = 12 retinas, 10 donors) after pseudobulk aggregation by sample and condition; genes with |log2 fold change| > 1 and FDR-adjusted *P* < 0.05 are highlighted. **l**, Volcano plot of differential gene expression comparing *CFH*-positive and *CFH*-negative nuclei after pseudobulk aggregation; genes with |log2 fold change| > 1 and FDR-adjusted *P* < 0.05 are highlighted. Statistical analyses used FDR-corrected two-sided Mann-Whitney *U* tests (**a–e**; only significant pairwise comparisons with resilient group shown), linear regression with FDR-adjusted *P* values (**h, i**), FDR-corrected differential expression analysis (**k, l**). AU, arbitrary units. Source data

To identify transcriptional features underlying resilience while reducing the influence of subtype-specific marker genes, we generated mixed pseudobulk profiles by aggregating total gene counts from the most susceptible and most resilient RGCs for each sample and condition, followed by differential expression analysis (Extended Data Fig. 2a–f). We defined several gene modules: module A (higher in resilient than susceptible RGCs; intrinsic resilience genes) was associated with synapse organization (such as *GRID2*, *SYNDIG1* and *LRRTM3*), axonogenesis (*NGF*, *SEMA5B*, *EFNA5* and *SLIT2*), complement system (*CFH*) and potassium ion transmembrane transport (*KCNJ16*, *KCNB2* and *CHRNA2*); module B (higher in susceptible than resilient RGCs; intrinsic susceptibility genes) was mainly involved in glutamatergic and calcium signalling (*GRIA4*, *GRIN2B*, *GRIN2C* and *CACNG2*); module C comprised genes upregulated during MS in resilient RGCs associated with stress responses (*JUN*, *JUND*, *FOSB*, *ACSL4* and *SQSTM1*) and inflammatory signalling (*NFKBIA*, *NFKBIZ* and *TGFB2*); module D contained transcripts downregulated in resilient RGCs in MS, indicating suppression of neuronal identity (*TBR1*, *POU4F1* and *EBF2*) and excitability programs (*DLGAP1*, *SORCS1* and *GRIN2C*), alongside downregulation of metabolic and redox-defence pathways (*GPT2* and *PSAT1*); module E comprised genes upregulated in susceptible RGCs during MS, reflecting extracellular-matrix remodelling (*DCN*, *SCUBE1* and *BMP5*) and mitochondrial stress (*MT-ATP8*, *MT-ND3*, *MT-ND4* and *MT-ND4L*); and module F comprised six downregulated transcripts in susceptible RGCs during MS. We validated modules A–E using AUCell analysis of pseudobulked susceptible, intermediate-susceptible, intermediate-resilient and resilient RGCs (Extended Data Fig. 3a–e), and assessed the resilience (module A) and susceptibility (module B) signatures across all 27 RGC subtypes (Extended Data Fig. 3f,g).

We next performed linear regression between resilience (change in relative frequency during MS; Fig. 1f) and pseudobulked gene expression, identifying genes that are positively (resilience candidates) and negatively (susceptibility candidates) associated with resilience (Fig. 1j,k and Extended Data Fig. 3h,i). *CFH* and *GPR149* emerged as the strongest, most biologically plausible candidates from both analyses. *CFH* was enriched in resilient RGC populations (module A), particularly RGC13 and RGC26 (Extended Data Fig. 3j), correlated positively with resilience (Fig. 1j) and was upregulated in RGCs from people with MS (Extended Data Fig. 3k), suggesting an adaptive neuronal stress response. *CFH* encodes a secreted glycoprotein that is the principal inhibitor of the alternative complement pathway and is abundant in plasma^17^. Notably, *CFH* variants have been implicated in neurodegenerative diseases, and its expression in retinal pigment epithelium has been studied in AMD, where the Y402H CFH variant accounts for over 40% of genetic risk for AMD^18–20^.

Conversely, *GPR149* was enriched in susceptible RGCs (module B), correlated with increasing susceptibility (Fig. 1k), was de-enriched in *CFH*-expressing RGCs (Extended Data Fig. 3l) and has a single-nucleotide polymorphism associated with increased MS risk^21^.

Multiplex single-molecule fluorescence in situ hybridization (smFISH) validated our bioinformatic findings, showing significant preservation of *CFH*-expressing RGCs in MS retinas, while the relative frequency of *GPR149*-expressing RGCs was reduced (Fig. 1l,m). Moreover, several other module A/B genes were recovered by the regression analysis (Extended Data Fig. 3h,i). As *CFH* expression correlated with baseline resilience and increased further in MS, we focused on it as a candidate mediator of both intrinsic and adaptive neuroprotection.

## Neuronal CFH is neuroprotective

**Fig. 2:**
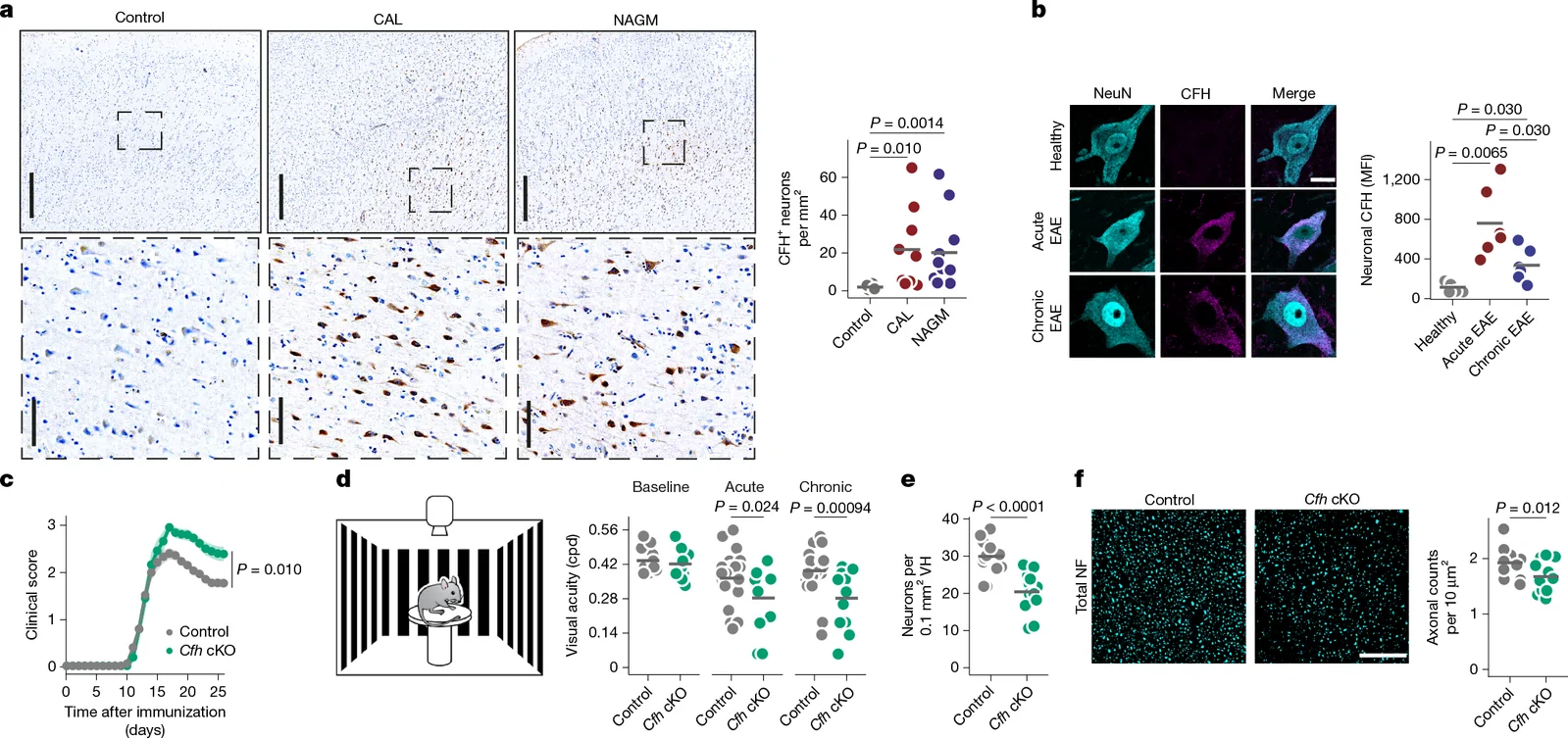
CFH protects neurons from inflammation-induced neurodegeneration. **a**, Quantification of CFH-positive neurons in post-mortem human cortex from chronic active lesions (CAL, *n* = 10 donors), normal-appearing grey matter (NAGM, *n* = 11 donors) of individuals with primary or secondary progressive MS, and control individuals (*n* = 5). Scale bars, 500 µm (overview) and 100 µm (magnified images). **b**, The neuronal CFH mean fluorescence intensity (MFI) in spinal cord motor neurons of healthy (*n* = 6), acute (*n* = 6) and chronic (*n* = 6) EAE mice. Scale bar, 15 µm. **c**, EAE clinical disease course of *Cfh*^*flox/flox*^ mice injected with AAV-hSYN1-GFP (control; *n* = 51) compared with AAV-hSYN1-cre to generate neuron-specific *Cfh* knockout (*Cfh*-KO; *n* = 22). Statistical comparisons were performed on the area under the curve (AUC). Data are mean ± s.e.m. **d**, Schematic of visual acuity measurements using the Optodrum system and analysis of visual acuity (cycles per degree, cpd) in control and *Cfh*-cKO mice at the baseline (1 week before EAE induction; control, *n* = 11; *Cfh*-cKO, *n* = 10), acute EAE (3–4 days after disease onset; control, *n* = 29; *Cfh*-cKO, *n* = 13) and chronic EAE (control, *n* = 31; *Cfh*-cKO, *n* = 12). **e**,**f**, Ventral horn (VH) neuronal counts in control (*n* = 15) or *Cfh*-cKO (*n* = 18) EAE mice (**e**) and axonal integrity at day 26 after immunization. Representative images (left) and quantification of axon numbers in the dorsal column as assessed by total neurofilament staining (right) (control, *n* = 16; *Cfh*-cKO, *n* = 17) (**f**). Scale bar, 100 µm. Individual datapoints represent biological replicates. The bars indicate the mean. Statistical comparisons were performed using FDR-corrected two-sided Mann–Whitney *U*-tests between groups (**b**) against controls (**a**, **c**, **e** and **f**) and within each timepoint (**d**). Source data

**Extended Data Fig. 4:**
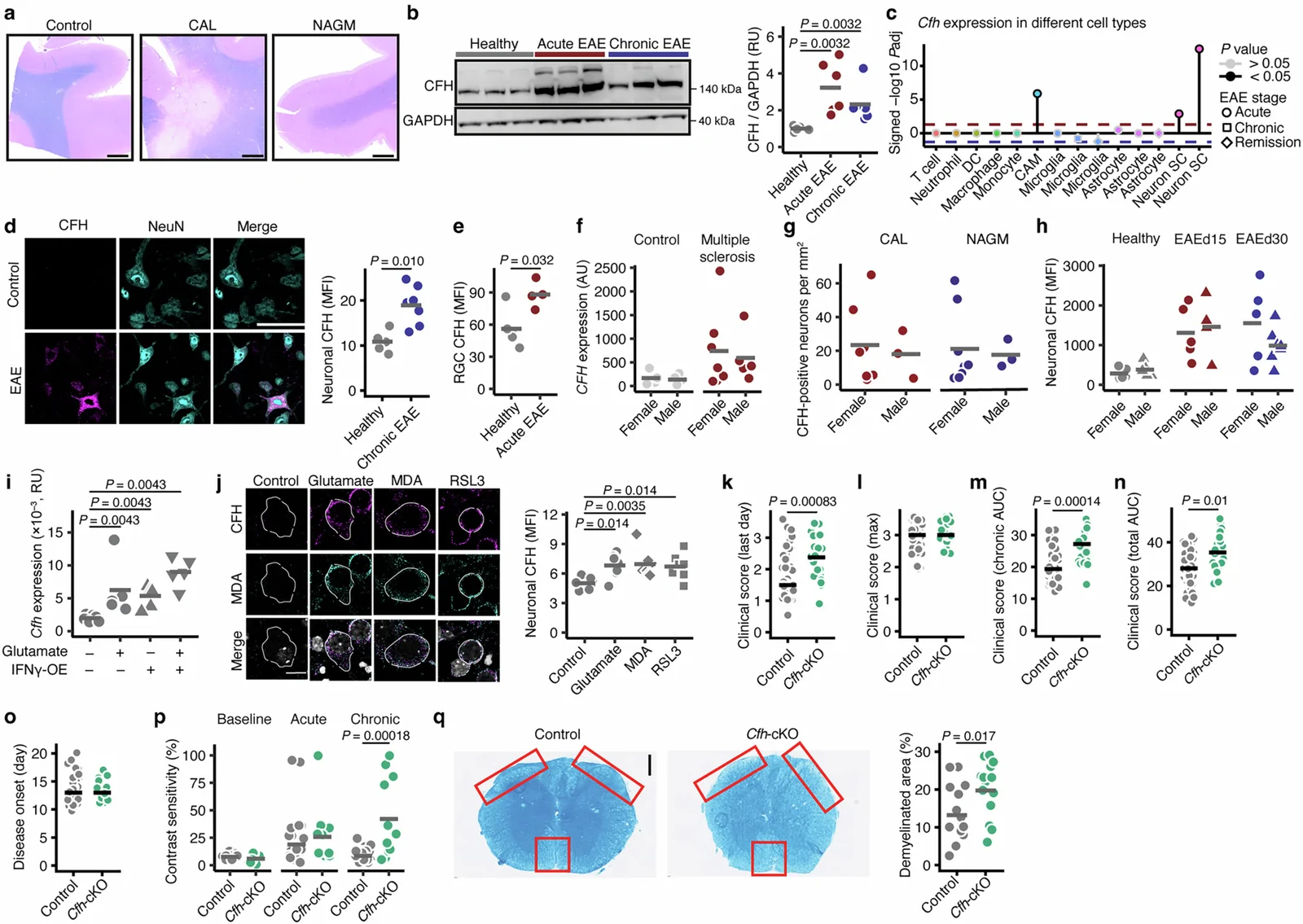
CFH is upregulated in neurons during EAE. **a**, Luxol fast blue (LFB) staining of human cortex used for CFH immunohistochemistry shown in Fig. 2a. Representative images show control cortex (left), a chronic active lesion (CAL, middle) and normal-appearing grey matter (NAGM, right). Scale bar, 2.5 mm. **b**, Immunoblot quantification of CFH normalized to GAPDH in spinal cord lysates from healthy (*n* = 6), acute (*n* = 6), and chronic (*n* = 6) EAE mice. **c**, Analysis of published spinal cord sequencing data sets showing *Cfh* regulation during EAE in the indicated cell populations. Dashed lines indicate FDR-adjusted *P* < 0.05. **d**, Representative images and CFH MFI in spinal cord motor neurons of healthy (*n* = 5) and EAE SJL mice (37 days after induction; *n* = 7). Scale bar, 50 µm. **e**, CFH MFI in RBPMS-positive retinal ganglion cells (RGCs) of healthy (*n* = 5) and acute EAE C57BL/6 mice (*n* = 4). **f**, Pseudobulk *CFH* expression in RGCs from female controls (*n* = 4), male controls (*n* = 6), female MS (*n* = 7) and male MS (*n* = 5). **g**, CFH-positive neurons per mm^2^ from Fig. 2a stratified by sex in CAL (female: *n* = 7; male: *n* = 3) and NAGM (female: *n* = 8; male: *n* = 3). **h**, Neuronal CFH MFI in spinal cord motor neurons from female and male healthy and acute EAE mice (*n* = 5 per group), and female (*n* = 5) and male chronic EAE mice (*n* = 7). **i**, *Cfh* transcript expression measured by qRT-PCR in primary neurons following glutamate treatment, chronic exposure to IFNγ by overexpression (IFNγ-OE) or combined stimulation (*n* = 5 per group; controls, *n* = 6; *Tbp* housekeeping gene). **j**, Representative images and neuronal CFH (MFI) of primary neurons stimulated with glutamate, MDA, or RSL3 (*n* = 7 per group). Scale bar, 20 µm. **k–o**, Clinical EAE parameters in control (*n* = 51) and *Cfh*-cKO mice (*n* = 22): clinical score on the final observation day (**k**), maximum score (**l**), chronic phase area under the curve (AUC; days 15–26 p.i.) (**m**), total AUC (**n**), and disease onset (**o**). **p**, Visual contrast sensitivity at baseline in control and *Cfh*-cKO mice (control, *n* = 11; *Cfh*-cKO, *n* = 10), acute EAE (control, *n* = 29, *Cfh*-cKO, *n* = 13) and chronic EAE (control, *n* = 31; *Cfh*-cKO, *n* = 12). **q**, Representative LFB staining of cervical spinal cord sections in control and *Cfh*-cKO mice at day 26 post-immunization and quantification of demyelinated white matter area in the indicated region (control, *n* = 16; *Cfh*-cKO, *n* = 16). Scale bar, 500 µm. Individual data points represent biological replicates; bars indicate the mean or median (**k–o**). Statistical comparisons used FDR-corrected two-sided unpaired Mann-Whitney *U* tests against controls or between sexes (**f-h**). AU, arbitrary units; MFI, mean fluorescence intensity; RU, relative units. Source data

To examine whether neuronal CFH induction during CNS inflammation extends beyond the retina and represents a general feature of CNS neurons, we performed immunohistochemistry analysis of post-mortem cortex from donors with MS and control donors without inflammatory diseases (Fig. 2a, Extended Data Fig. 4a and Supplementary Table 2). Notably, CFH expression was minimal to absent in control individuals but was significantly elevated during MS both in chronic active lesions and normal-appearing grey matter, with upregulation predominantly localized to neurons (Fig. 2a).

In experimental autoimmune encephalomyelitis (EAE), CFH protein increased significantly in spinal-cord motor neurons of C57BL/6 mice during the acute and chronic phase of EAE (Fig. 2b). This was corroborated by immunoblotting of EAE spinal cords (Extended Data Fig. 4b), and by published cell-type-specific mRNA datasets^22–26^ showing increased CFH primarily in neurons and CNS-associated macrophages, with no significant changes in other cell types (Extended Data Fig. 4c). Confirming that this is a general response across MS models, neuronal CFH was similarly induced during chronic EAE in SJL/JRj (hereafter, SJL) mice, a relapsing–remitting model^27,28^ (Extended Data Fig. 4d). Analogous to the human retina, we observed a significant increase in CFH in RGCs of the C57BL/6 EAE mouse retina (Extended Data Fig. 4e). In CNS inflammation, CFH levels were comparable between sexes across human RGCs, cortical neurons and mouse spinal motor neurons (Extended Data Fig. 4f–h).

To identify CFH-inducing stimuli, we exposed primary mouse neurons to interferon-γ (IFNγ) and glutamate. *Cfh* expression was induced not only by the combination of IFNγ and glutamate but also by each stimulus individually (Extended Data Fig. 4i). As glutamate causes oxidative stress among other effects, we applied the GPX4 inhibitor (1*S*,3*R*)-RSL3 (RSL3) as a more targeted oxidative stressor, which is known to induce lipid peroxidation^29^, and the neurotoxic aldehyde malondialdehyde (MDA)^30^. Immunocytochemistry analysis confirmed a significant increase in CFH protein under all conditions, indicating that inflammatory and oxidative stressors independently induce neuronal CFH (Extended Data Fig. 4j).

To determine its functional relevance in vivo, we used an adeno-associated virus (AAV) expressing Cre recombinase under the neuron-specific human synapsin 1 (h*SYN1*) promoter to achieve neuronal *Cfh* deficiency in *Cfh*^*flox/flox*^ mice before inducing EAE (Supplementary Fig. 2a–c). Neuronal *Cfh*-deficient mice showed a more severe disease course and greater visual impairment affecting both visual acuity and contrast sensitivity (Fig. 2c,d and Extended Data Fig. 4k–p), corroborated by increased neuronal loss in the ventral horn of the spinal cord (Fig. 2e) and accompanied by more extensive axonal damage and demyelination (Fig. 2f and Extended Data Fig. 4q). Notably, neuronal *Cfh* deficiency did not alter the peripheral immune cell compartment or immune cell infiltration into the spinal cord during acute EAE (Supplementary Figs. 3 and 4). Together, CFH is upregulated in neurons during MS and EAE, probably as a stress response, and its absence exacerbates disease severity and visual impairment, demonstrating a critical role in protecting neurons against inflammation-induced degeneration.

## CFH expression rescues neurons

**Fig. 3:**
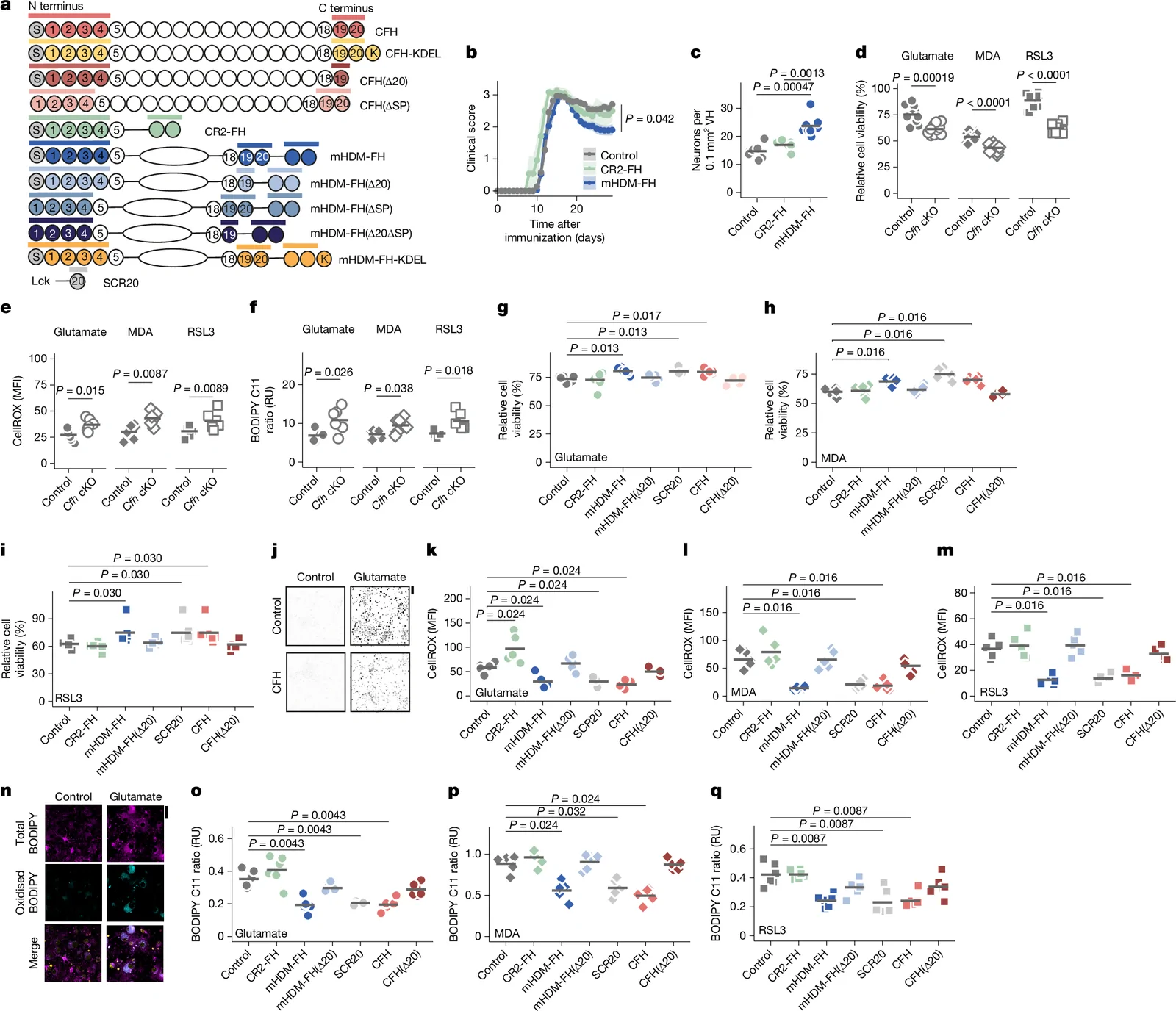
Neuronal CFH expression protects against neurodegeneration in EAE. **a**, Schematic of the truncated and modified CFH variants used in this study. N- and C-terminal SCR domains are indicated. S, signal peptide; K, KDEL; Lck, lymphocyte-specific protein tyrosine kinase. **b**, EAE disease course in female C57BL/6J mice with AAV-mediated neuronal expression of GFP (control, *n* = 16), mouse homodimeric minimal factor H (mHDM-FH, *n* = 16) or CR2-FH (*n* = 6) under the neuron-specific h*SYN1* promoter. Statistical analysis was performed on the chronic disease AUC (days 20–29 after immunization). Data are mean ± s.e.m. **c**, Ventral horn neuronal counts in GFP controls (*n* = 8), mHDM-FH (*n* = 8) and CR2-FH (*n* = 6) EAE mice. **d**–**f**, Primary cortical neurons from *Cfh*^*flox/flox*^ mice transduced with AAV-hSYN1-GFP (control) or AAV-hSYN1-cre (*Cfh*-cKO). Cell viability (**d**; *n* = 11), ROS measured by CellROX (**e**; *n* = 6) and lipid peroxidation measured by BODIPY C11 (**f**; *n* = 6) after glutamate, MDA or RSL3 stimulation. **g**–**i**, Cell viability of neurons expressing the indicated CFH constructs after glutamate (**g**; *n* = 6), MDA (**h**; *n* = 5) or RSL3 (**i**; *n* = 6) treatment. **j**, Representative CellROX images of unstimulated and glutamate-stimulated neurons. Scale bar, 20 µm. **k**–**m**, CellROX MFI in neurons expressing the indicated CFH constructs after glutamate (**k**), MDA (**l**) or RSL3 (**m**) treatment. *n* = 5. **n**, Representative BODIPY C11 images after glutamate stimulation. Scale bar, 20 µm. **o**–**q**, BODIPY C11 ratios in neurons expressing the indicated CFH constructs after glutamate (**o**; *n* = 6), MDA (**p**; *n* = 5) or RSL3 (**q**; *n* = 6) treatment. Individual datapoints represent biological replicates. The bars indicate the mean. Statistical comparisons were performed using unpaired two-sided Mann–Whitney *U*-tests between groups (**b** and **c**) against controls (**d** and **g–q**), or unpaired two-sided *t*-tests against controls (**e** and **f**). RU, relative units. Source data

**Extended Data Fig. 5:**
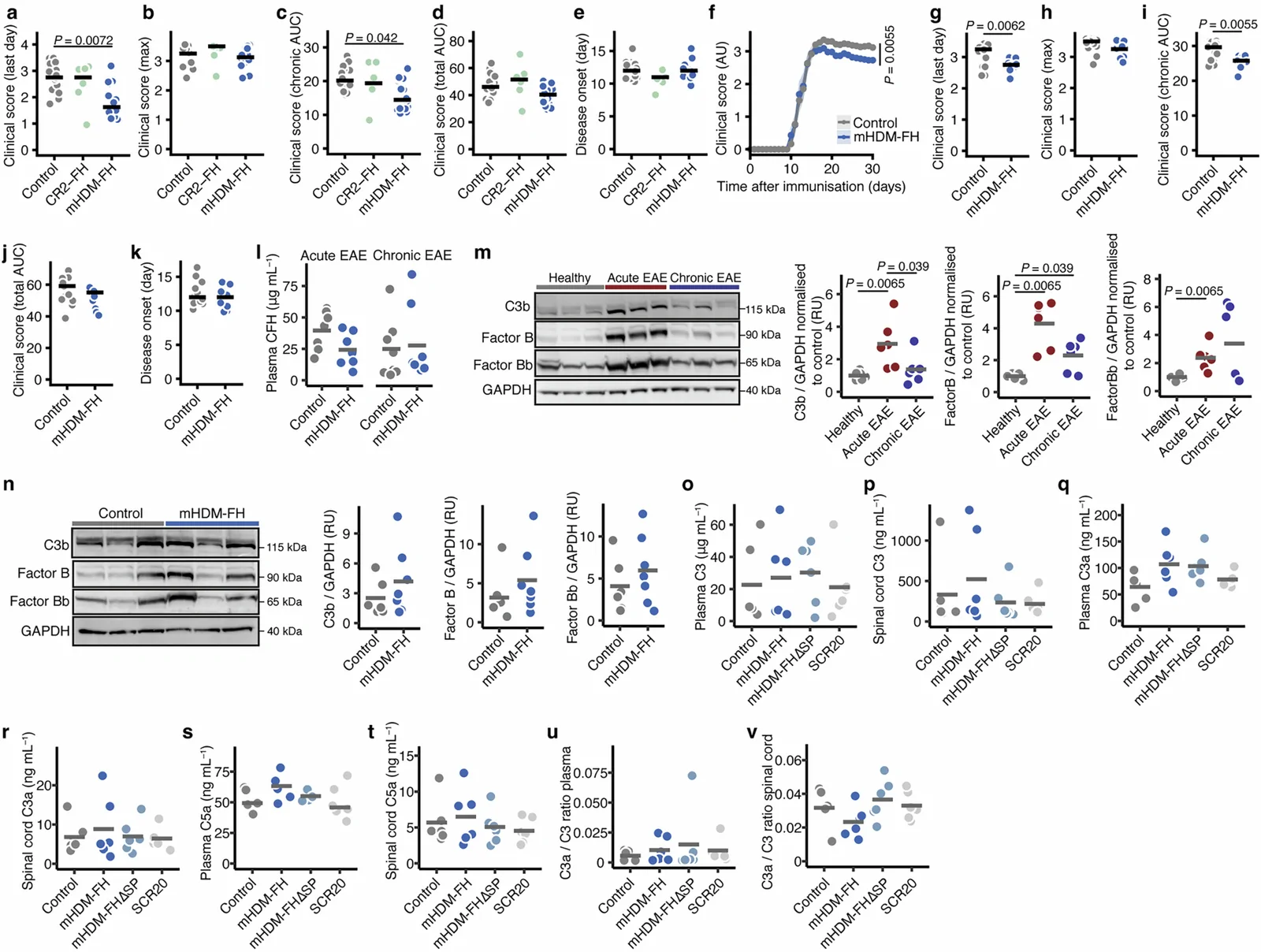
Neuronal CFH construct expression does not alter complement activation during EAE. **a–e**, Clinical EAE parameters in mice expressing neuronal GFP (*n* = 16), mHDM-FH (*n* = 16), or CR2-FH (*n* = 6): clinical score at day 29 post-immunization (p.i) (**a**), maximum clinical score (**b**), chronic phase area under the curve (AUC; 20–29 days p.i.) (**c**), total AUC (days 0–29 p.i.) (**d**), and disease onset (**e**). **f–k**, EAE clinical course (**f;** mean ± s.e.m.; symbols indicate the mean and shaded areas the s.e.m.) and corresponding clinical parameters (**g–k**) in male mice expressing neuronal GFP (*n* = 15), or mHDM-FH (*n* = 11): clinical score at day 30 p.i. (**g**), maximum clinical score (**h**), chronic phase AUC (20–30 days p.i) (**i**), total AUC (0–30 days p.i.) (**j**) and disease onset (**k**). **l**, Plasma CFH concentrations measured by ELISA in mice expressing neuronal GFP (acute, *n* = 7; chronic, *n* = 9) or mHDM-FH (acute, *n* = 7; chronic, *n* = 8). **m**,**n**, Immunoblot quantification of C3b, factor B, and factor Bb normalized to GAPDH in spinal cord lysates from healthy, acute, and chronic EAE mice (**m**; *n* = 6 per group; shown relative to healthy controls), or day 15 p.i. EAE mice expressing neuronal GFP (control, *n* = 6) or mHDM-FH (*n* = 7) (**n**). The blots in **m** share the same loading control as Extended Data Fig. 4b. **o–v**, Plasma and spinal cord concentrations of C3 (**o,p**), C3a (**q,r**), C5a (**s,t**), and C3a/C3 ratios (**u,v**), measured by ELISA in acute EAE mice (day 15 p.i.) expressing neuronal GFP, mHDM-FH, mHDM-FHΔSP (*n* = 6 per group), or SCR20 (*n* = 6 for **o,p,r–t,v**; *n* = 5 for **q,u**). Individual data points represent biological replicates; bars indicate the mean or median (**a–e, g–k**). Statistical comparisons used FDR-corrected two-sided unpaired Mann-Whitney *U* tests. RU, relative units. Source data

To evaluate the therapeutic potential of neuronal CFH and elucidate its mechanism of action, we examined whether expression could pre-emptively protect against inflammation-induced neurodegeneration. Given the large size of CFH, we used two published truncated versions to compare its N- and C-terminal domains. The first, mouse homodimeric minimal factor H (mHDM-FH), is an effective cofactor for human factor-I-mediated cleavage of mouse C3b and reduces glomerular C3 staining in *Cfh*-deficient mice^31,32^. The second, CR2-FH, is a fusion of a complement receptor 2 fragment with the N-terminal short consensus repeat (SCR) domains 1–5 of CFH, reported to reduce choroidal neovascularization in an AMD model when delivered by AAV^33^ (Fig. 3a). At 3 weeks before EAE induction, we administered AAVs encoding mHDM-FH, CR2-FH or a GFP control intravenously under the h*SYN1* promoter for neuron-specific CNS expression (Supplementary Fig. 5a–c), without relevant expression in the enteric nervous system (Supplementary Fig. 5d). Notably, only mHDM-FH significantly reduced EAE scores in both male and female mice and neuronal loss in the chronic phase (20–29 days after immunization), whereas CR2-FH had no measurable effect (Fig. 3b,c and Extended Data Fig. 5a–k).

To determine whether protection reflected altered immune responses, we performed a comprehensive immune cell characterization using flow cytometry during acute EAE and found no significant differences in the immune cell infiltrate or activation between mHDM-FH- and GFP-expressing mice (Supplementary Fig. 6a–e). Analysis using an enzyme-linked immunosorbent assay (ELISA) validated the unchanged plasma CFH levels (Extended Data Fig. 5l). We also examined C3b and factor Bb, which together constitute the alternative-pathway C3 convertase that is inhibited by CFH. Both were significantly elevated in the spinal cord during acute EAE (Extended Data Fig. 5m), but did not differ between mHDM-FH- and GFP-expressing animals (Extended Data Fig. 5n). As plasma and spinal cord levels of C3, C3a and C5a and the C3a/C3 ratio at peak disease also did not differ between constructs (Extended Data Fig. 5o–v), there was no evidence that complement activation contributed to the phenotype. Thus, neuronal mHDM-FH, but not CR2-FH, protects against inflammatory neurodegeneration without altering the immune response or directly inhibiting the alternative complement pathway.

## CFH reduces neuronal oxidative stress

**Extended Data Fig. 6:**
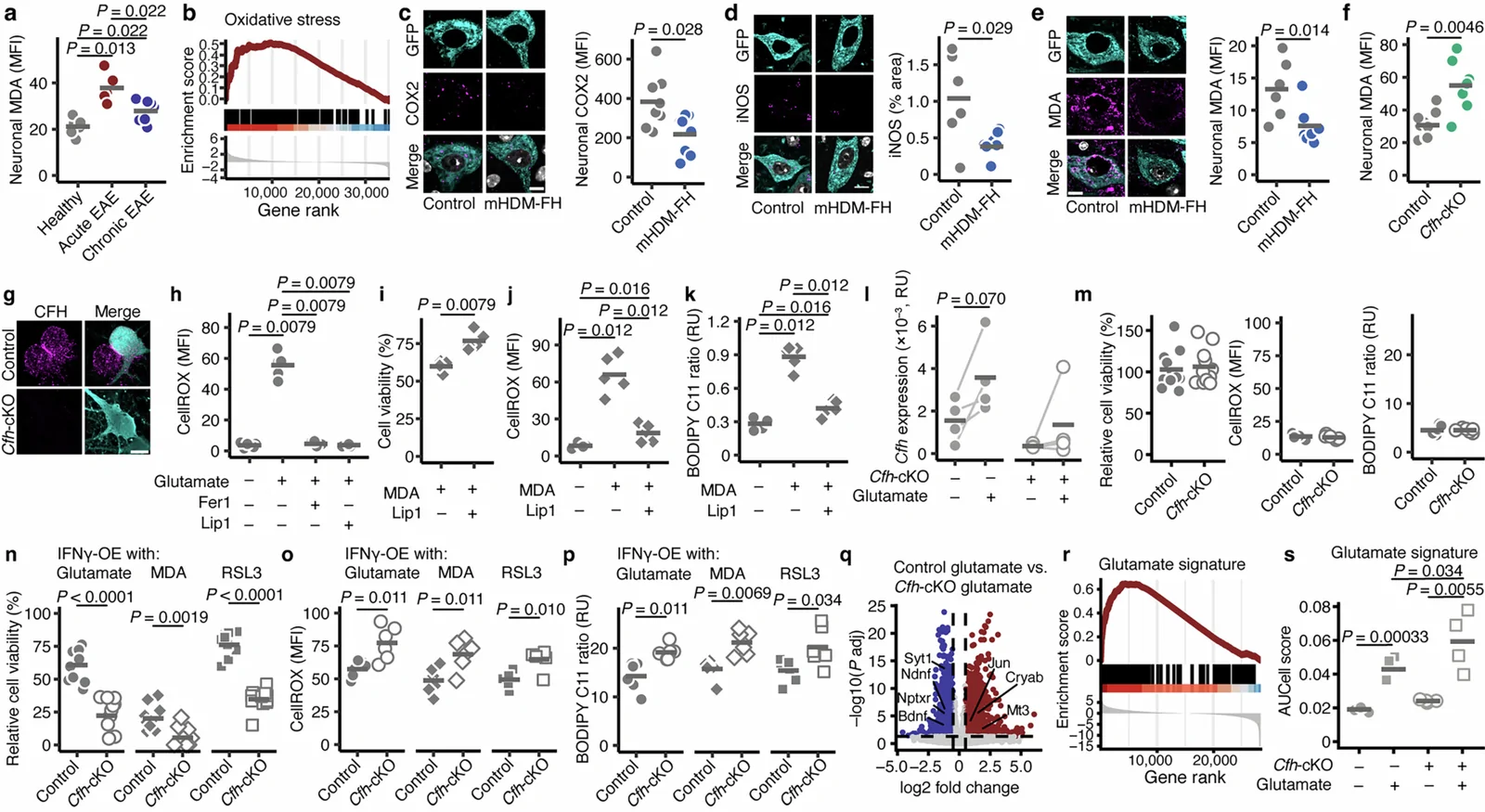
CFH mitigates neuronal oxidative stress. **a**, Neuronal malondialdehyde (MDA) MFI in spinal cord motor neurons from healthy (*n* = 6), acute (*n* = 5), and chronic EAE (*n* = 7) mice. **b**, Gene set enrichment analysis of the term “response to oxidative stress” in pseudobulked retinal ganglion cells (RGCs) from MS (*n* = 12 retinas, 10 donors) compared with controls (*n* = 10 retinas, 10 donors). **c–e**, Neuronal cyclooxygenase-2 (COX2) MFI (**c**; *n* = 8 per group), inducible nitric oxide synthase (iNOS)-positive neuronal area (**d**; control, *n* = 6; mHDM-FH, *n* = 8), and MDA-modified protein MFI (**e**; control, *n* = 6; mHDM-FH, *n* = 7) in the spinal cord ventral horn (VH) of chronic EAE mice expressing neuronal GFP (control) or mHDM-FH. Scale bar, 10 µm. **f**, Neuronal MDA-modified protein MFI in VH of control (*n* = 10) or *Cfh*-cKO (*n* = 7) mice. **g**, Representative images of *Cfh*^*flox/flox*^ primary neurons transduced with AAV-hSyn1-GFP (control) or AAV-hSyn1-Cre (*Cfh*-cKO). AAVs co-expressed a fluorophore (cyan). Scale bar, 10 µm. **h–k**, Effects of ferrostatin-1 (Fer1) or liproxstatin-1 (Lip1) pretreatment on glutamate- or MDA-induced reactive oxygen species (CellROX), cell viability, and lipid peroxidation (BODIPY C11) in primary neurons (*n* = 5 per condition). **l**, *Cfh* mRNA expression measured by qRT–PCR in control or *Cfh*-cKO neurons with or without glutamate treatment (*n* = 4 per condition; *Tbp* housekeeping gene). **m**, Baseline cell viability (*n* = 11 per condition), ROS (CellROX MFI, *n* = 6 per condition), and lipid peroxidation (BODIPY C11 MFI, *n* = 6 per condition) in control and *Cfh*-cKO neurons. **n–p**, Relative cell viability (**n**; *n* = 11), ROS (**o**; *n* = 6), and lipid peroxidation (**p**; *n* = 6) in control and *Cfh*-cKO neurons chronically exposed to IFNγ by overexpression (IFNγ-OE) and stimulated with glutamate, MDA, or RSL3. **q–s**, mRNA sequencing of the neuronal cultures shown in **l** (*n* = 4 per condition): volcano plot with differentially expressed genes with |log2 fold change | > 0.5 and FDR-adjusted *P* value < 0.05 highlighted (**q**), gene set enrichment analysis using a top 200 glutamate-response signature (**r**), and AUCell analysis of all glutamate-induced genes (**s**). Individual data points represent biological replicates; bars indicate the mean. Statistical comparisons used FDR-corrected unpaired two-sided Mann-Whitney *U* tests (**a,c–k**) against controls (**m–n**), FDR-corrected unpaired *t* tests against controls (**o–p**), paired two-sided *t* test (**l**) and FDR-corrected pairwise comparisons of estimated marginal means (**s**). MFI, mean fluorescence intensity; RU, relative units. Source data

mHDM-FH, but not CR2-FH, contains the C-terminal SCR18–20 domains, implicating them in neuroprotection. Whereas N-terminal SCR1–4 confer complement-regulatory activity, C-terminal SCR18–20 mediate binding to cell surfaces and dying cells by recognizing danger signals including sialic-acid-containing glycosaminoglycans, heparan sulphates, surface-bound C3b and MDA^34^. MDA is a byproduct of lipid peroxidation, an indicator of oxidative stress and a highly reactive aldehyde that modifies nearby proteins and lipids. Its levels were increased in neurons during EAE (Extended Data Fig. 6a), indicating oxidative damage, lipid peroxidation and ferroptosis^35^, all of which are implicated in neuronal damage in MS and EAE^36–38^. Consistently, our pseudobulk analysis of MS versus control RGCs confirmed pronounced enrichment of oxidative-stress signatures (Extended Data Fig. 6b). mHDM-FH-expressing neurons showed reduced levels of the oxidative-stress markers COX2 and inducible nitric oxide synthase as well as MDA-modified proteins (Extended Data Fig. 6c–e), whereas MDA-modified proteins were increased in neuronal *Cfh*-deficient mice during EAE (Extended Data Fig. 6f), leading us to hypothesize that CFH acts by mitigating oxidative stress.

For mechanistic studies, we generated neuronal *Cfh*-knockout cells in vitro by transducing *Cfh*^*flox/flox*^ neurons with AAV-hSYN1-cre (*Cfh-*cKO; Extended Data Fig. 6g) and used lentiviral vectors to expand our construct repertoire, generating full-length CFH and additional truncated variants (Fig. 3a). On the basis of the hypothesis that neuroprotection is mediated by the C-terminal domains, we also created constructs lacking SCR20, lacking the signal peptide for intracellular retention and a construct comprising the SCR20 domain alone^39^.

To induce oxidative damage, we applied three stimuli: glutamate, an excitotoxic agent that increases reactive oxygen species (ROS), promotes lipid peroxidation and activates ferroptosis (Extended Data Fig. 6h); MDA, which can bind to CFH^40^, reduces neuronal viability in a manner partly rescued by the ferroptosis inhibitor liproxstatin-1 (Extended Data Fig. 6i) and increases ROS and lipid peroxidation, suggesting that it sensitizes cells to ferroptosis in a feed-forward manner (Extended Data Fig. 6j,k); and the GPX4 inhibitor RSL3, which directly induces lipid peroxidation. Glutamate induced *Cfh* in wild-type neurons, but not in *Cfh*-cKO neurons (Extended Data Fig. 6l), with no baseline differences in viability, ROS or lipid peroxide content between genotypes (Extended Data Fig. 6m). After glutamate, MDA or RSL3 treatment, *Cfh*-cKO neurons showed reduced viability (Fig. 3d), increased ROS (Fig. 3e) and increased lipid peroxidation (Fig. 3f). These differences were further amplified by chronic IFNγ exposure (Extended Data Fig. 6n–p), which exacerbates ferroptosis susceptibility^37^. Bulk RNA-seq also revealed an increased excitotoxicity signature in *Cfh*-cKO neurons treated with glutamate (Extended Data Fig. 6q–s). Conversely, CFH construct expression conferred significant protection against all stressors, but only when constructs included SCR20 (Fig. 3g–q).

**Extended Data Fig. 7:**
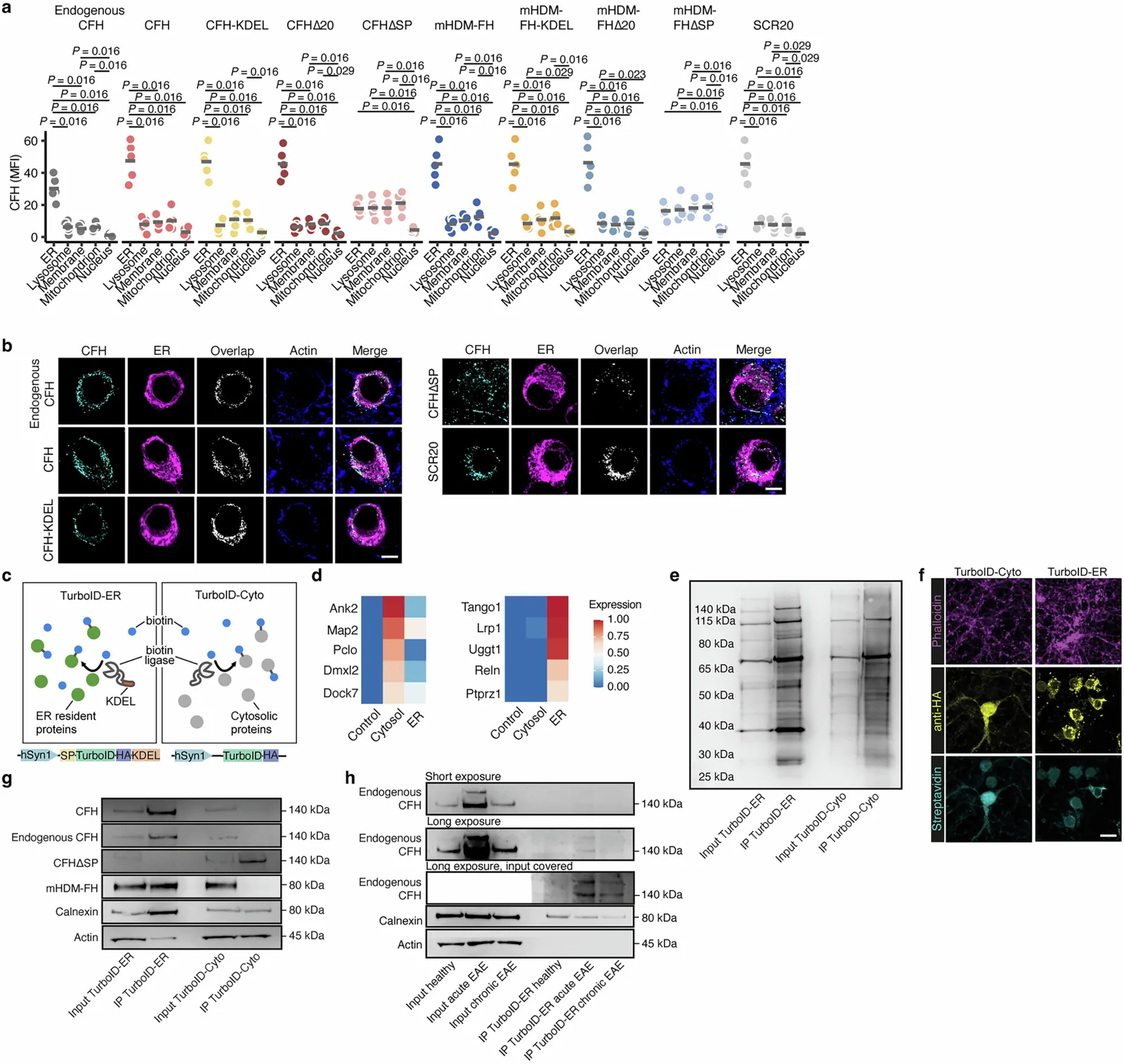
CFH localizes to the ER in neurons. **a**, Quantification of CFH mean fluorescence intensity (MFI) within the indicated subcellular compartments for endogenous CFH induced by glutamate and for each CFH construct shown in Supplementary Fig. 7 (*n* = 5 per condition). **b**, Representative images showing ER localization of endogenous CFH after stimulation with glutamate, or of expressed CFH, CFH-KDEL, CFHΔSP, or SCR20. Scale bar, 5 µm. Representative of five independent experiments with similar results; quantification shown in a. **c**, Schematic of TurboID proximity labelling of ER-localized (TurboID-ER) and cytosolic (TurboID-Cyto) proteins. **d**, Mass spectrometry validation of cytosolic (left) and ER (right) protein enrichment in mouse primary cortical neurons following streptavidin immunoprecipitation of biotin-labelled proteins. Untransduced neurons served as controls (*n* = 2 per condition). **e**, Immunoblot validating TurboID-mediated biotinylation following streptavidin immunoprecipitation in primary neurons expressing TurboID-ER or TurboID-Cyto under the hSyn1 promoter. **f**, Representative image of primary neuronal cultures transduced with lentiviral vectors carrying the constructs shown in **c**. Scale bar, 10 µm. **g**, Immunoblot of primary neuronal cultures transduced with the constructs shown in **c** and co-transduced to express CFH, CFHΔSP, or mHDM-FH. Endogenous CFH was induced by chronic IFNγ exposure followed by glutamate stimulation. Calnexin and β-actin validated enrichment of ER-localized and cytosolic proteins, respectively. **h**, Immunoblot of spinal cord lysates from one healthy mouse, one mouse with acute EAE and one with chronic EAE following AAV-mediated neuronal expression of TurboID-ER. The experiments shown in **e-h** were each performed once. Individual data points represent biological replicates; bars indicate the mean. Statistical comparisons used FDR-corrected unpaired two-sided Mann-Whitney *U* tests. Source data

The subcellular localization of each construct was validated by immunocytochemistry in primary neurons (Supplementary Fig. 7a–j and Extended Data Fig. 7a,b), Pfa1 fibroblasts^41^ (Supplementary Fig. 8a–i) and HEK293T cells (Supplementary Fig. 9a–i). CFH, CFH-KDEL, mHDM-FH, mHDM-FH-KDEL and the SCR20 subunit localized predominantly to the endoplasmic reticulum (ER), whereas the signal-peptide-deficient CFH(ΔSP) and mHDM-FH(ΔSP) variants distributed diffusely, partially co-localizing with ER, lysosomes, mitochondria and nucleus. To corroborate this, we performed proximity labelling with an engineered biotin ligase, TurboID^42^, targeted to the cytoplasm (TurboID-Cyto) or ER lumen (TurboID-ER), validating compartment-specific enrichment in cortical neurons (Extended Data Fig. 7c–f). Both endogenous and expressed CFH and mHDM-FH localized to the ER, whereas CFH(ΔSP) was mainly detected in the cytoplasm (Extended Data Fig. 7g). In vivo, neuron-specific ER-targeted TurboID revealed that endogenous neuronal CFH was largely undetectable at steady state but became robustly induced and enriched in the ER fraction during EAE (Extended Data Fig. 7h). Together, neuronal CFH localizes to the ER and reduces oxidative stress and lipid peroxidation in an SCR20-dependent manner.

## SCR20 of CFH limits lipid peroxidation

**Extended Data Fig. 8:**
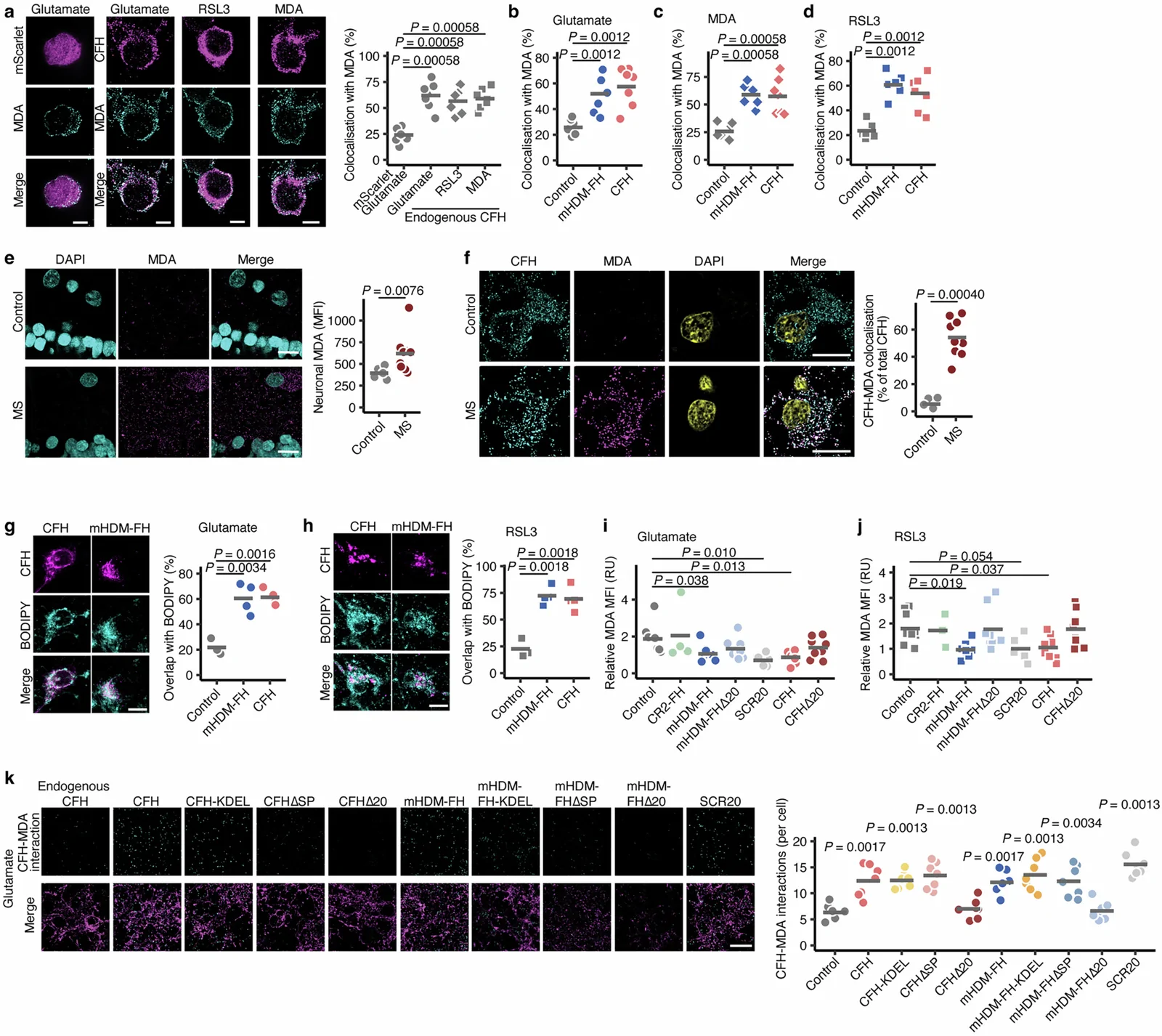
MDA colocalises with CFH in neurons. **a**, Colocalisation of MDA-modified proteins with mScarlet or endogenous CFH in primary cortical neurons following glutamate (mScarlet, *n* = 6; CFH, *n* = 7), RSL3 (*n* = 7) or MDA (*n* = 7) treatment. Representative images (left) and quantification (right). Scale bar, 5 µm. **b–d**, Colocalisation of CFH with MDA-modified proteins in neurons expressing mScarlet, CFH, or mHDM-FH (*n* = 7) following glutamate (**b**), MDA (**c**), or RSL3 (**d**) treatment. **e**,**f**, Representative images (left) and quantification (right) of MDA-modified protein MFI (**e**) and CFH colocalisation with MDA-modified proteins, expressed as the percentage of total CFH signal (**f**), in retinal ganglion cells (RGCs) from control (*n* = 6) and MS (*n* = 9) retinas. Scale bars, 50 µm (**e**) and 25 µm (**f**). **g**,**h**, Colocalisation of BODIPY C11 with CFH in neurons expressing mScarlet control (*n* = 3), CFH (*n* = 4), or mHDM-FH (*n* = 4) following glutamate (**g**) or RSL3 (**h**) treatment. Scale bar, 5 µm. **i,j**, Neuronal MDA-modified protein MFI relative to unstimulated controls in neurons expressing mScarlet, CR2-FH, mHDM-FH, mHDM-FHΔ20, SCR20, CFH, or CFHΔ20 following glutamate (**i**) or RSL3 (**j**) treatment. Sample sizes for **i**: mScarlet (*n* = 9), CR2-FH (*n* = 4), mHDM-FH (*n* = 8), mHDM-FHΔ20 (*n* = 8), SCR20 (*n* = 6), CFH (*n* = 8), CFHΔ20 (*n* = 8); for **j**, mScarlet (*n* = 9), CR2-FH (*n* = 3), mHDM-FH (*n* = 9), mHDM-FHΔ20 (*n* = 8), SCR20 (*n* = 5), CFH (*n* = 8), CFHΔ20 (*n* = 8). **k**, Proximity ligation assay for CFH and MDA-modified proteins after glutamate stimulation. Representative images (left) and quantification of puncta per cell (right) (*n* = 7 biological replicates per construct). Scale bar, 50 µm. Individual data points represent biological replicates; bars indicate the mean. Statistical comparisons used FDR-corrected unpaired two-tailed Mann-Whitney *U* tests against controls (**a–f,k**), or FDR-corrected unpaired two-tailed *t*-tests against controls (**g–j**). MFI, mean fluorescence intensity; RU, relative units. Source data

Neurons expressing SCR20-containing constructs of CFH showed significantly lower levels of ROS and lipid peroxidation after glutamate, MDA or RSL3 exposure, whereas neurons expressing SCR20-lacking constructs exhibited markedly higher levels (Fig. 3j–q). CFH has been identified as a binding partner of MDA-modified proteins in the plasma^40,43^, so we examined whether it also associates with them intracellularly under oxidative stress. Co-localization of CFH with MDA-modified proteins increased significantly after glutamate, MDA or RSL3 treatment, for both endogenous and lentivirally delivered CFH (Extended Data Fig. 8a–d). In post-mortem human retina, MDA-modified proteins increased significantly in MS (Extended Data Fig. 8e) with enhanced CFH–MDA co-localization (Extended Data Fig. 8f). CFH also overlapped strongly with the lipid-peroxidation marker BODIPY C11 after glutamate and RSL3 treatment (Extended Data Fig. 8g,h), and constructs containing SCR20 accumulated significantly fewer MDA-modified proteins (Extended Data Fig. 8i,j). To further validate this spatial association, proximity ligation assays using the CFH variants, which were reliably detected with an antibody directed against CFH (Supplementary Figs. 7–9), showed no proximity under control conditions (Supplementary Fig. 10a) but a significant increase after glutamate treatment (Extended Data Fig. 8k). A similar increase was observed in the GFP controls, consistent with the upregulation of endogenous CFH, whereas no signal was detected in *Cfh*-cKO cultures (Supplementary Fig. 10b).

**Fig. 4:**
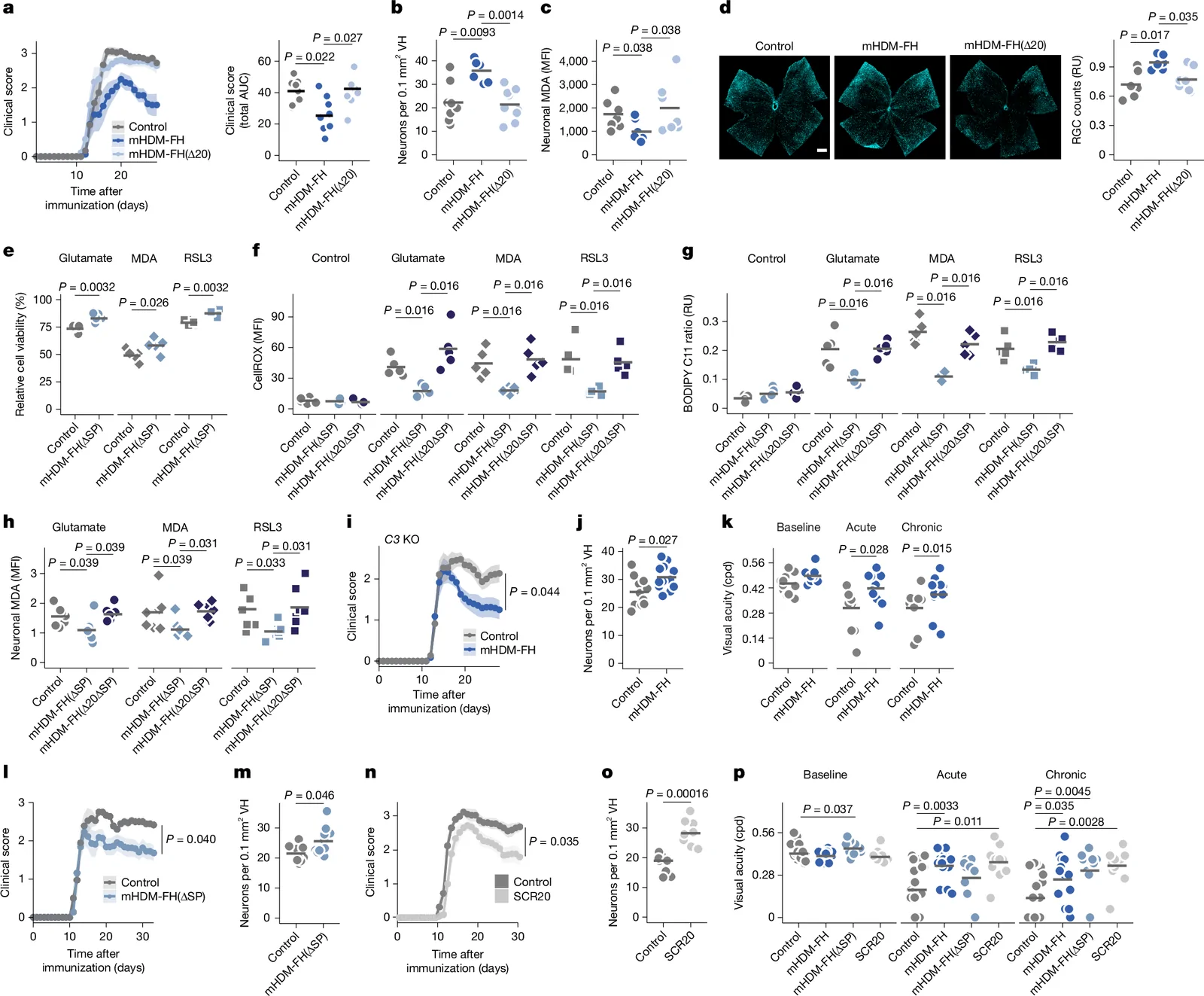
Intracellular CFH protects neurons against oxidative stress. **a**, EAE disease course and total AUC in female C57BL/6J mice after AAV-mediated neuronal expression of GFP (*n* = 9), mHDM-FH (*n* = 8) or mHDM-FH(Δ20) (*n* = 9). **b**,**c**, Ventral horn neuronal density (**b**; GFP, *n* = 8; mHDM-FH, *n* = 7; mHDM-FH(Δ20), *n* = 9) and neuronal MDA MFI (**c**; GFP, *n* = 8; mHDM-FH, *n* = 7; mHDM-FH(Δ20), *n* = 7) at day 30 after immunization. **d**, GFP-positive RGC survival at day 30 after immunization, normalized to the healthy controls (GFP, *n* = 6; mHDM-FH, *n* = 6; mHDM-FH(Δ20), *n* = 7). Scale bar, 500 µm. **e**–**h**, The relative cell viability (**e**; *n* = 6), ROS (CellROX MFI; **f**; *n* = 5), lipid peroxidation (BODIPY C11 ratio; **g**; *n* = 5) and neuronal MDA MFI (**h**; *n* = 7) in neurons expressing mScarlet (control), mHDM-FH(ΔSP) or mHDM-FH(Δ20ΔSP) after glutamate, MDA or RSL3 treatment. **i**–**k**, EAE disease course (**i**), ventral horn neuronal density at day 28 after immunization (**j**) and visual acuity (**k**) in *C3*-KO mice expressing neuronal GFP or mHDM-FH (disease course: GFP, *n* = 11; mHDM-FH, *n* = 12; visual acuity: baseline, GFP, *n* = 13; mHDM-FH, *n* = 9; acute, GFP, *n* = 10; mHDM-FH, *n* = 11; chronic, GFP, *n* = 11; mHDM-FH, *n* = 12). **l**–**o**, EAE disease course (**l**,**n**) and ventral horn neuronal density at day 30 after immunization (**m**,**o**) after neuronal expression of GFP or mHDM-FH(ΔSP) (**l**,**m**; GFP, *n* = 9; mHDM-FH(ΔSP), *n* = 11), or GFP or SCR20 (**n**,**o**; disease course: GFP, *n* = 8; SCR20, *n* = 11; neuronal density: *n* = 8 per group). **p**, Visual acuity in mice expressing GFP and mHDM-FH (*n* = 21 per timepoint), mHDM-FH(ΔSP) or SCR20 (*n* = 12 per timepoint) at baseline and at acute and chronic disease timepoints. Datapoints represent biological replicates; bars indicate the median (**a**) or mean (**b**–**p**). For the EAE curves, data are mean ± s.e.m. (**a**, **i**, **l** and **n**), and the AUC was analysed. Statistical comparisons were performed using FDR-corrected two-sided unpaired Mann–Whitney *U*-test comparisons against mHDM-FH (**a**–**d**), mHDM-FH(ΔSP) (**e**–**h**) or GFP (**i**–**p**). Source data

**Extended Data Fig. 9:**
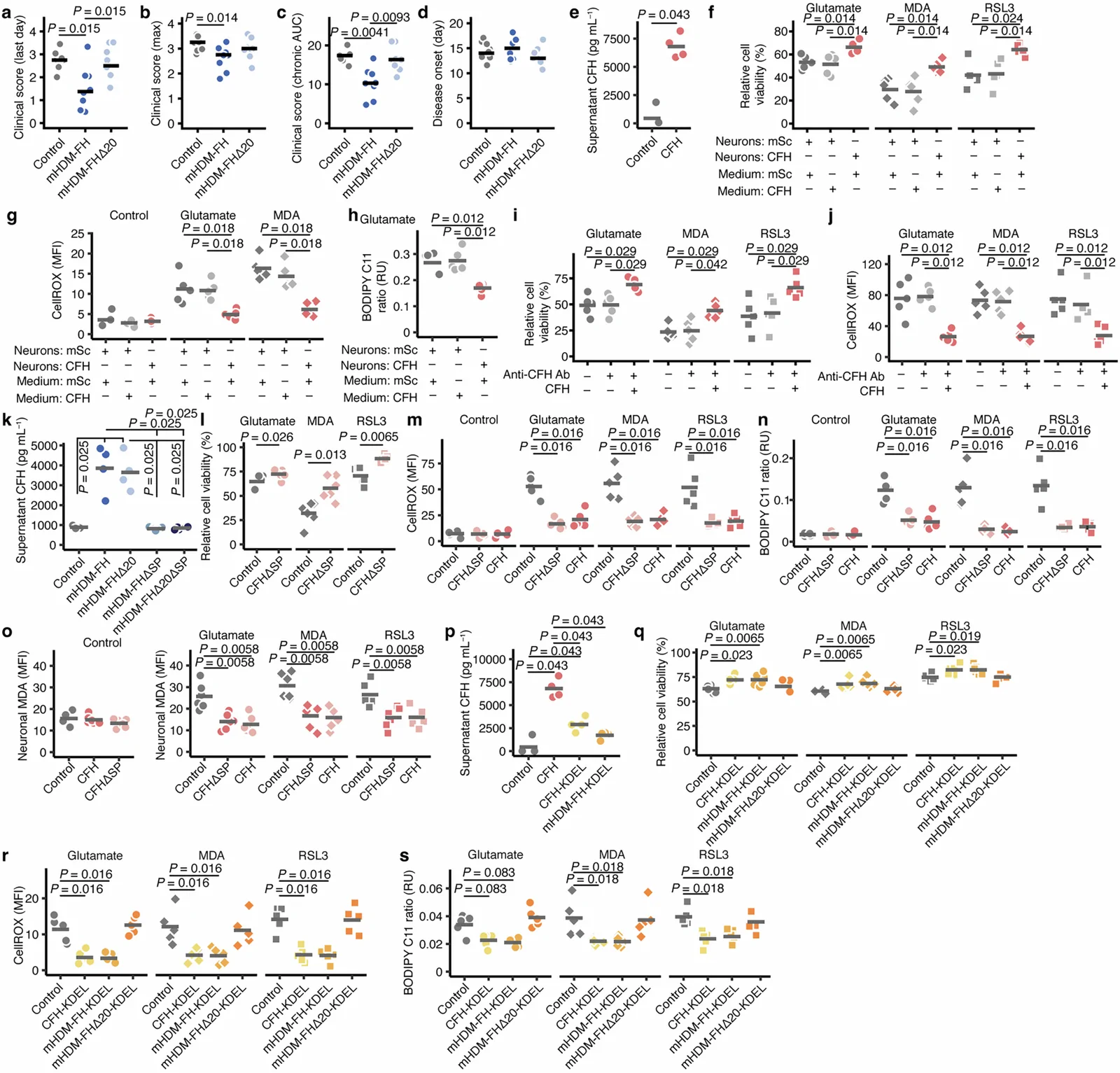
Intracellular but not secreted CFH confers neuroprotection. **a–d**, Clinical EAE parameters at day 30 post-immunization in mice expressing neuronal GFP (*n* = 9), mHDM-FH (*n* = 8), or mHDM-FHΔ20 (*n* = 9): clinical score (**a**), maximum clinical score (**b**), chronic area under the curve (AUC) (**c**), and disease onset (**d**). **e**, CFH concentrations measured by ELISA in supernatant of neuronal cultures expressing mScarlet control or CFH (*n* = 4 per group). **f–h**, Relative cell viability (**f**), reactive oxygen species (ROS; CellROX MFI; **g**), and lipid peroxidation (BODIPY C11 ratio) (**h**) in mScarlet- or CFH-expressing neurons following medium exchange and glutamate, MDA, or RSL3 treatment (*n* = 5 per group); mScarlet cultures without medium exchange served as controls (*n* = 5). **i**,**j**, Relative cell viability (**i**), and ROS (**j**) in mScarlet or CFH-expressing neurons treated as in **f–h** in the presence of a CFH C-terminal blocking antibody (*n* = 5 per group); untreated mScarlet cultures served as controls (*n* = 5). **k**, ELISA quantification of CFH in supernatants of neurons expressing mScarlet (*n* = 3), mHDM-FH (*n* = 4), mHDM-FHΔ20 (*n* = 4), mHDM-FHΔSP (*n* = 8), or mHDM-FHΔ20ΔSP (*n* = 7). **l–o**, Relative cell viability (**l**), ROS (**m**), lipid peroxidation (**n**), and neuronal MDA-modified protein MFI (**o**) in neurons expressing mScarlet control, CFH, or CFHΔSP following glutamate, MDA, or RSL3 treatment (cell viability and MDA MFI, *n* = 6; ROS and lipid peroxidation, *n* = 5). **p**, ELISA quantification of CFH in supernatants of neurons expressing mScarlet, CFH, CFH-KDEL, or mHDM-FH-KDEL (*n* = 4). The mScarlet and CFH data shown in **p** are identical to those shown in **e**. **q–s**, Relative cell viability (**q**), ROS (**r**), and lipid peroxidation (**s**) in neurons expressing mScarlet, CFH-KDEL, mHDM-FH-KDEL, or mHDM-FHΔ20-KDEL following glutamate, MDA, or RSL3 treatment (*n* = 5). Individual data points represent biological replicates; bars indicate the mean or median (**a–d**). Statistical comparisons used FDR-corrected two-sided unpaired Mann-Whitney *U* tests (**e–j**) against mHDM-FH (**a–d**) or controls (**l–s**), or FDR-corrected two-sided unpaired *t* tests (**k**). MFI, mean fluorescence intensity; RU, relative units. Source data

We next examined whether CFH-mediated protection extended to other cell types and cell death modalities. In Pfa1 fibroblasts, MDA or RSL3 reduced viability and increased lipid peroxidation, both rescued by the radical-trapping agent liproxstatin-1 (Supplementary Fig. 11a,b). SCR20-containing CFH constructs conferred robust protection, preserving viability and markedly reducing lipid peroxidation after RSL3 treatment (Supplementary Fig. 11c,d). Protection was ferroptosis specific, as MDA-modified proteins increased only after RSL3-induced ferroptosis, not after apoptosis, necroptosis or proteotoxic stress (Supplementary Fig. 11e), and neither CFH nor mHDM-FH protected against these non-ferroptotic pathways (Supplementary Fig. 11f). Consistently, while ferrostatin-1 and liproxstatin-1 alone conferred some protection against glutamate-induced stress, their combination with CFH or mHDM-FH expression added no further benefit, probably because CFH already exerted substantial protection (Supplementary Fig. 11g,h). To test SCR20 dependence in vivo, AAVs encoding mHDM-FH, the truncated mHDM-FH(Δ20) (lacking SCR20) or GFP under the h*SYN1* promoter were delivered 3 weeks before EAE (Fig. 4a and Extended Data Fig. 9a–d). mHDM-FH, but not mHDM-FH(Δ20), conferred significant neuroprotection, preserving ventral horn neuronal counts, reducing MDA levels and significantly protecting transduced RGCs (Fig. 4b–d). Thus, the C-terminal SCR20 domain is essential for neuroprotection against oxidative stress in neuronal and non-neuronal cells.

## CFH acts intracellularly in neurons

Despite the established extracellular functions of CFH, including binding MDA modifications on cell surfaces^40^ and regulating complement^39,43^, our data support an intracellular mechanism in neurons. We first confirmed that CFH was secreted into the supernatant after lentiviral transduction (Extended Data Fig. 9e). To test whether extracellular CFH contributes to protection, we performed a medium-exchange experiment; primary cultures expressing CFH or an mScarlet control construct were incubated with swapped medium before stimulation with glutamate, MDA or RSL3. Applying medium from CFH-expressing cultures to mScarlet cultures did not improve viability, ROS or lipid peroxidation, whereas CFH-expressing cultures retained protection when given mScarlet medium (Extended Data Fig. 9f–h). Similarly, a C-terminal CFH-blocking antibody^44^ did not diminish protection of viability or ROS production after stimulation with glutamate, MDA or RSL3 (Extended Data Fig. 9i,j), further supporting an intracellular mechanism independent of extracellular C-terminal interactions.

To confirm that intracellular CFH is sufficient for neuroprotection, we generated signal-peptide-deficient variants (Fig. 3a and Supplementary Figs. 7e,i, 8c,f and 9c,f) that were undetectable in the supernatant by ELISA (Extended Data Fig. 9k). Intracellular mHDM-FH(ΔSP) protected against cell death, ROS production, lipid peroxidation and accumulation of MDA-modified proteins, whereas mHDM-FH(Δ20ΔSP) did not (Fig. 4e–h). Likewise, CFH(ΔSP) was as neuroprotective as CFH (Extended Data Fig. 9l–o). We next engineered KDEL-tagged constructs to retain CFH and mHDM-FH in the ER (Fig. 3a and Supplementary Figs. 7c,g, 8b,e and 9b,e); ELISA confirmed significantly reduced secretion (Extended Data Fig. 9p) and these ER-retained constructs also robustly reduced cell death, ROS and lipid peroxidation after glutamate, MDA and RSL3 (Extended Data Fig. 9q–s). To understand how differently localized variants confer protection, we mapped the subcellular distribution of lipid peroxidation. Consistent with reports implicating the ER and lysosomes as primary ferroptotic sites^45–47^, lipid peroxidation localized early to lysosomes and the ER in Pfa1 cells after RSL3 (Supplementary Fig. 12a–f) and predominantly to the ER, followed by lysosomes, mitochondria, other membranes and cytosol, in neurons (Supplementary Fig. 13a–f). These findings support ER involvement in neuronal ferroptosis and are consistent with protection by ER-retained CFH and mHDM-FH variants.

## CFH counteracts IFNγ-driven stress

**Extended Data Fig. 10:**
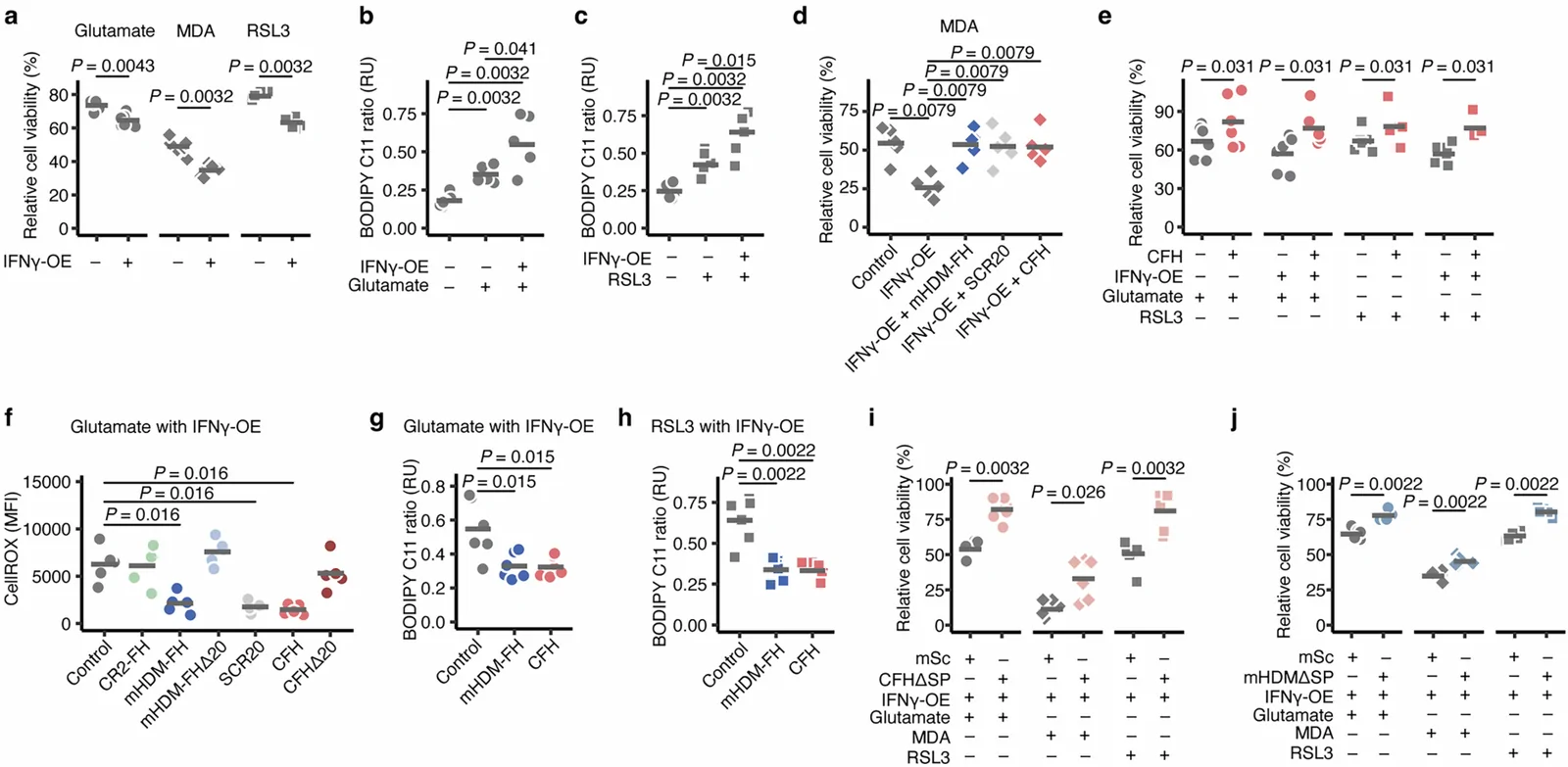
CFH counteracts IFNγ-exacerbated oxidative stress. **a**, Relative cell viability of primary cortical neurons following glutamate, MDA, or RSL3 treatment, with or without chronic IFNγ overexpression (*n* = 6 per condition). **b,c**, Lipid peroxidation (BODIPY C11 ratio, RU) of primary neuronal cultures after glutamate (**b**), or RSL3 (**c**) treatment, with or without chronic IFNγ overexpression (*n* = 6 per condition). Unstimulated cultures served as controls. **d,e** Relative cell viability following MDA stimulation of neurons expressing mScarlet, mHDM-FH, SCR20 or CFH (**d**) or following RSL3 treatment of mScarlet- or CFH-expressing neurons (**e**), with or without chronic IFNγ overexpression (*n* = 5 per condition). **f**, Reactive oxygen species (ROS; CellROX mean fluorescence intensity, MFI) in primary neurons expressing mScarlet, CR2-FH, mHDM-FH, mHDM-FH∆20, SCR20, CFH, or CFHΔ20 after chronic IFNγ overexpression and glutamate treatment (*n* = 5 per construct). **g,h**, Lipid peroxidation (BODIPY C11 ratio, RU) in primary neurons expressing mScarlet, mHDM-FH, or CFH following chronic IFNγ overexpression and glutamate (**g**), or MDA (**h**) treatment (*n* = 6 per condition and construct). **i,j**, Relative cell viability after glutamate, MDA, or RSL3 treatment of neurons expressing CFHΔSP (**i**) or mHDM-FHΔSP (**j**) compared with mScarlet controls after chronic IFNγ overexpression (*n* = 6 per condition). Individual data points represent biological replicates; bars indicate the mean. Statistical comparisons used FDR-corrected two-sided unpaired Mann-Whitney *U* tests (**b–d**) against controls (**a,f–j**), or FDR-corrected two-sided paired Mann-Whitney *U* tests (**e**). IFNγ-OE, IFNγ overexpression. Source data

Given that MS is characterized by inflammation-driven neurodegeneration, we examined whether CFH confers protection under inflammatory conditions. To model this, we chronically exposed primary neurons to IFNγ by overexpression before ferroptotic stimulation, which markedly exacerbated oxidative stress as viability declined further after glutamate, MDA or RSL3 exposure (Extended Data Fig. 10a), and lipid peroxidation increased after treatment with glutamate and RSL3 (Extended Data Fig. 10b,c). CFH, mHDM-FH and SCR20 each rescued the IFNγ-augmented reduction in viability after MDA (Extended Data Fig. 10d), glutamate or RSL3 treatment (Extended Data Fig. 10e), reduced ROS after glutamate treatment (Extended Data Fig. 10f) and attenuated lipid peroxidation after glutamate or MDA exposure (Extended Data Fig. 10g,h). CFH(ΔSP) and mHDM-FH(ΔSP) were comparably protective against IFNγ-exacerbated neuronal loss (Extended Data Fig. 10i,j). Together, IFNγ amplifies ferroptotic neuronal death, and this inflammation-enhanced toxicity is mitigated by SCR20-containing intracellular CFH constructs, directly supporting a protective role for CFH against oxidative stress in an inflammatory milieu.

## CFH protection persists without C3

**Extended Data Fig. 11:**
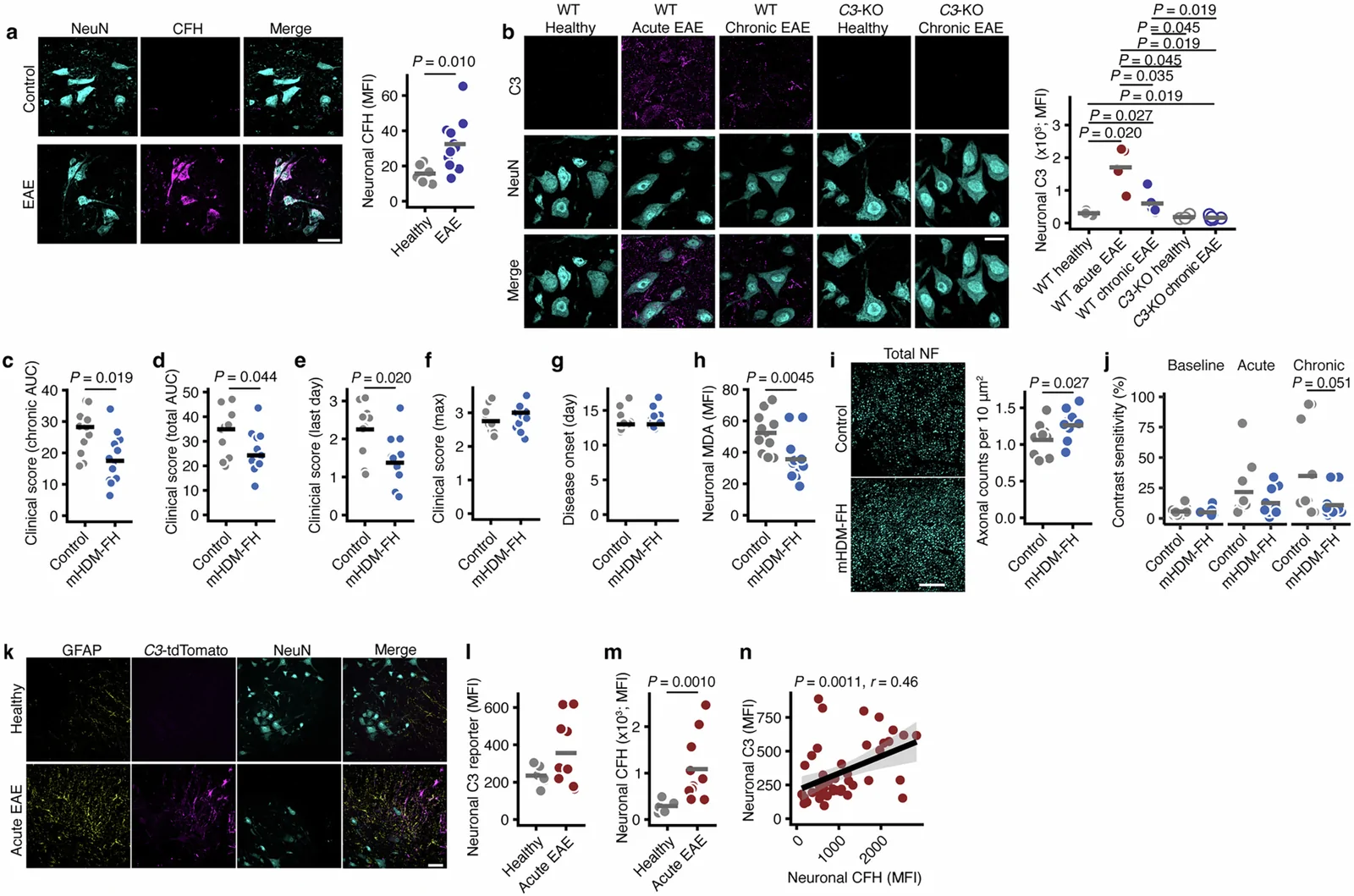
Neuroprotective effects of mHDM-FH are retained in the absence of C3. **a**, Neuronal CFH representative images (left) and MFI (right) in the spinal cord ventral horn (VH) of healthy C3 knockout (*C3*-KO) mice (*n* = 6) and EAE *C3*-KO mice at day 28 post-immunization (p.i.; *n* = 11). Scale bar, 50 µm. **b**, Neuronal C3 representative images (left) and MFI (right) in spinal cord VH neurons of healthy wild-type (WT) mice (*n* = 6), acute (*n* = 5) and chronic WT EAE mice (*n* = 5), healthy *C3*-KO mice (*n* = 3), and chronic EAE *C3*-KO mice (*n* = 7). Scale bar, 20 µm. **c–g**, Clinical EAE parameters from Fig. 4i in *C3*-KO mice expressing neuronal GFP (control, *n* = 11) or mHDM-FH (*n* = 12): post-acute area under the curve (AUC) (15–28 days p.i.) (**c**), total AUC (**d**), final clinical score (**e**), maximum clinical score (**f**), and disease onset (**g**). **h,i** Neuronal MDA-modified protein levels in VH spinal cord (**h**) and dorsal column axon numbers with representative neurofilament staining (**i**) in *C3*-KO mice expressing GFP (control, *n* = 11) or mHDM-FH (*n* = 12) at day 28 p.i. Scale bar, 50 µm. **j**, Contrast sensitivity in *C3*-KO mice expressing GFP or mHDM-FH at baseline (control: *n* = 13; mHDM-FH, *n* = 9), during acute disease (control, *n* = 10; mHDM-FH, *n* = 11), and during chronic disease (control, *n* = 11; mHDM-FH, *n* = 12). **k**, Representative spinal cord images from healthy and acute EAE C3-tdTomato reporter mice. Scale bar, 50 µm. **l,m**, Neuronal tdTomato (**l**) and CFH (**m**) MFI in healthy control (*n* = 6) and acute EAE (*n* = 10) *C3*-tdTomato reporter mice. **n**, Spearman correlation between neuronal CFH and C3 MFI (*n* = 47 neurons from 10 acute EAE mice). Individual data points represent biological replicates (**a–m**) or individual neurons (**n**); bars indicate the mean or median (**c–g**) and the shaded area the 95% confidence interval. Statistical comparisons used FDR-corrected two-sided unpaired Mann-Whitney *U* test (**a–m**). Correlation was assessed using a two-sided Spearman’s rank correlation test (**n**). MFI, mean fluorescence intensity. Source data

Recent work shows that CFH can regulate intracellular C3 in macrophages cell autonomously, with *Cfh* deficiency increasing intracellular C3 consumption independently of downstream complement activation^48^. To test whether a similar mechanism, or an interaction between CFH and C3b through its C-terminal SCR19–20 domains, accounts for the observed neuroprotection, we performed EAE experiments in *C3*-deficient (*C3*-KO) mice. As in wild-type mice, neuronal CFH increased significantly during EAE (Extended Data Fig. 11a), while neuronal C3 was undetectable in *C3*-KO mice (Extended Data Fig. 11b). We next transduced *C3*-KO mice with AAVs driving neuronal GFP or mHDM-FH 3 weeks before EAE. Consistent with previous reports, *C3*-KO mice developed an overall ameliorated disease course^49^; nevertheless, neuronal mHDM-FH expression further reduced severity, improved neuronal survival (Fig. 4i,j and Extended Data Fig. 11c–g), decreased neuronal accumulation of MDA-modified proteins (Extended Data Fig. 11h), preserved axonal integrity (Extended Data Fig. 11i) and maintained visual function (Fig. 4k and Extended Data Fig. 11j) in the absence of C3. To assess whether neuronal CFH indirectly modulates C3, we used C3-tdTomato reporter mice^50^ (Extended Data Fig. 11k). At peak disease, intraneuronal C3 showed a non-significant upward trend (Extended Data Fig. 11l) while neuronal CFH was markedly elevated (Extended Data Fig. 11m), and the two correlated positively, consistent with co-regulation as part of a neuronal innate-immune response (Extended Data Fig. 11n). Thus, the protective effect of neuronal mHDM-FH is retained without neuronal C3, supporting a non-canonical mechanism.

## Intracellular CFH is neuroprotective in vivo

Finally, to determine whether intracellular CFH is protective in vivo, we delivered an AAV carrying mHDM-FH(ΔSP) under the h*SYN1* promoter 3 weeks before EAE. Mice expressing neuronal mHDM-FH(ΔSP) showed reduced severity during the chronic phase (20–30 days after immunization) without changes in disease onset or maximum score (Fig. 4l and Supplementary Fig. 14a–e), supported by reduced neuronal loss and diminished MDA-modified proteins (Fig. 4m and Supplementary Fig. 14f). To assess whether SCR20 alone suffices in vivo, we delivered an AAV encoding SCR20 under the same promoter; this similarly reduced severity (Fig. 4n and Supplementary Fig. 14g–k), neuronal loss (Fig. 4o) and MDA-modified proteins (Supplementary Fig. 14l), with attenuation of MDA and 4-hydroxynonenal levels already evident at peak disease (Supplementary Fig. 14m,n). Functionally, neuronal expression of mHDM-FH, mHDM-FH(ΔSP) or SCR20 significantly ameliorated deficits in visual acuity and contrast sensitivity (Fig. 4p and Supplementary Fig. 14o), reduced demyelination in the cervical and thoracic spinal cord (Supplementary Fig. 14p,q) and attenuated axonal damage (Supplementary Fig. 14r). Importantly, these constructs did not alter peripheral or central immune cell activation or infiltration (Supplementary Fig. 15a–d). To test generalizability across EAE models, we induced disease in SJL mice. Consistent with our other findings, mice expressing mHDM-FH, mHDM-FH(ΔSP) or SCR20 showed improved recovery after relapses, while the peak severity was unchanged (Supplementary Fig. 16a–d), together with reduced MDA and 4-hydroxynonenal levels, decreased neuronal loss and less axonal damage (Supplementary Fig. 16e–h). Together, CFH protects neurons through an intracellular antioxidative mechanism.

## Discussion

Using the retina as a discovery platform, we leveraged RGC heterogeneity to identify neuron-intrinsic determinants of resilience to inflammation (a graphical summary is shown in Supplementary Fig. 17). In the retina, light exposure generates free radicals, subjecting neurons to constant oxidative stress and making it an ideal environment for studying intrinsic neuronal defence mechanisms against oxidative damage, a hallmark of neurodegeneration in MS and other neurological diseases^1^. Midget RGCs, the retinal neurons of the parvocellular colour-vision pathway, were most vulnerable in MS, consistent with dyschromatopsia in optic neuritis and reports of preferential parvocellular degeneration after optic neuritis^51,52^. Melanopsin-expressing ipRGCs were also susceptible, echoing clinical evidence of early ipRGC loss and their vulnerability in Alzheimer’s disease^53,54^.

Among candidate genes, CFH correlated with RGC survival and was induced in MS. Notably, CFH was absent at the baseline in cortical and spinal motor neurons but was induced in MS and EAE, suggesting that constantly oxidatively stressed RGCs constitutively deploy a defence that other CNS neurons activate only under inflammation, representing a broadly conserved neuronal strategy. The identified intracellular function of CFH is distinct from its canonical secreted role and may reflect a distinct splice variant or conformation^55^. Consistent with this, the SCR7 region, which contains the AMD-associated Y402H variant, was dispensable for neuroprotection against oxidative stress^40,56^, whereas SCR20 was essential. Mechanistically, inflammatory (IFNγ) and oxidative-stress-induced neuronal CFH interrupted a self-perpetuating cycle of ROS accumulation, lipid peroxidation and ferroptosis in an SCR20-dependent, C3-independent manner. Protection was maintained when CFH was retained in the ER or cytoplasm, probably by sequestration of locally generated MDA-modified proteins, consistent with the spatial association between protective CFH constructs and MDA-modified proteins. Protection extended from RGCs to cortical and spinal motor neurons, guarding against both visual and motor deficits. While the lipid-peroxidation-dependent cell death process ferroptosis is a key driver of neuronal loss in MS^36–38^, our data position the ER as a prominent site of neuronal lipid peroxidation, as recently suggested in other cell types^35,45–47,57,58^. We identify intracellular CFH as a key regulator of the protective neuronal inflammatory stress response and a potential counterbalance to STING-driven GPX4 degradation^37,59^, positioning enhancement of intracellular CFH as a promising therapeutic strategy for inflammation-induced neurodegeneration.

## Methods

### Human tissue

#### Post-mortem human tissue samples

Eyeballs from individuals diagnosed with MS were obtained from the Netherlands Brain Bank, and those from control donors without recognizable neuropathological changes were obtained from Johns Hopkins University. Detailed information about donors used for snRNA-seq, immunohistochemistry, qPCR and smFiSH are provided in Supplementary Table 1. The macula region of these donors was used for snRNA-seq while smFISH was performed on tissue adjacent to the macula. qPCR and immunohistochemistry were conducted with the peripheral retina. Paraffin-embedded cortex tissue for histopathology was obtained from the UK Multiple Sclerosis Tissue Bank at Imperial College London. Samples were classified as chronic active lesions or normal appearing grey matter according to the histopathological assessment provided by the UK Multiple Sclerosis Tissue Bank’s histopathology reports. Although no statistical methods were used to predetermine sample size, our sample size is comparable to those reported in previous studies^12,13,60^.

#### Nucleus isolation and library preparation

Eyeballs were enucleated from deceased healthy controls and people with MS. Only tissue from patients with a post-mortem interval ≤24 h was used. To isolate the macula region, fresh-frozen eyeballs were positioned in a CM3050 S cryostat (Leica Microsystems) at –20 °C, with the lens facing downward and the optic disc oriented toward the examiner. The macula was identified as a distinct yellow spot on the retina, and the tissue was stored at −80 °C until use. All of the subsequent steps were performed on ice according to a previously published protocol with minor adaptations. In brief, on each experimental day, the frozen tissue was transferred to ice-cold NP40 lysis buffer (0.1% NP-40 (Thermo Fisher Scientific, 85124), 10 mM Tris pH 8.0, 1 mM CaCl_2_, 8 mM MgCl_2_, 15 mM NaCl, 0.02 U µl^–1^ DNase I (Merck Millipore, D4527)). The retina was transferred to a Dounce homogenizer in 1 ml lysis buffer supplemented with 0.2 U µl^−1^ Ribolock RNase Inhibitor (Thermo Fisher Scientific, EO0382) and homogenized 20 times with both loose and tight pestles. The homogenate was passed through a 100 µm cell strainer and centrifuged at 500*g* for 5 min. All buffers, except for the washing buffer, were supplemented with 0.16 U µl^−1^ Ribolock RNase inhibitor. Pelleted nuclei were resuspended in staining buffer (Tris base buffer: 10 mM Tris pH 8.0, 1 mM CaCl_2_, 8 mM MgCl_2_, 15 mM NaCl, 1 U ml^−1^ DNase I) containing 0.02% Tween-20 and 2% BSA, with primary antibodies against NeuN (1:250, Merck Millipore, ABN91) and RBPMS (1:250, Abcam, ab194213) for 15 min at 4 °C. Nuclei were washed, centrifuged at 500*g* for 5 min and stained with secondary antibodies (anti-chicken 647, 1:500; Jackson ImmunoResearch, 703-605-155; and anti-rabbit PE, 1:200, BioLegend, Poly4064) for 15 min at 4 °C. After another wash step, nuclei were filtered through a 70 µm strainer, resuspended in sorting buffer (Tris base buffer with 2% BSA), and Hoechst (1:2,000) was added to visualize nuclei. NeuN^+^RBPMS^+^ nuclei were sorted using the BD FACS Aria III device running BD FACSDiva v.9.0.1 into Ames medium (Sigma-Aldrich, A1420) with 1.5% BSA. Sorted NeuN^+^RBPMS^+^ nuclei were pelleted at 500*g* for 5 min at 4 °C, resuspended in approximately 20 µl of 1% BSA in PBS, visually inspected, counted in a Neubauer chamber and adjusted to a concentration of around 1,000 nuclei per µl. Nuclei were then loaded onto the 10x Chromium Single Cell Chip G (10x Genomics) with a targeted recovery of about 12,000 nuclei per channel. Libraries were generated according to the manufacturer’s protocol using the Chromium Single Cell 3′ Reagent Kit version 3.1 (dual index), measured using the Agilent Bioanalyzer (TapeStation 4150) and sequenced on the Illumina NovaSeq 6000 platform (paired-end), aiming for a sequencing depth of around 30,000 reads per nucleus.

#### Data preprocessing and quality control

Count matrices for each sample were generated by aligning each library to the human reference mRNA transcriptome GRCh38-2020-A using Cell Ranger (v.7.0.1), including both exonic and intronic reads^61^. The Cell Ranger output was processed using CellBender v.0.3.0 with the default settings (epochs = 150, fpr = 0.01, learning rate = 10^−4^) to remove ambient RNA and other background noise^62^. Downstream analysis was performed with Seurat (v.5)^63^ in R Studio (R v.4.4.1). For each RNA count matrix, the following steps were carried out: cell counts were normalized to a total library size of 10,000 and log transformed (Seurat, NormalizeData). The top 2,000 variable features were identified (Seurat, FindVariableFeatures), followed by data scaling (Seurat, ScaleData) and dimensionality reduction (Seurat, RunPCA, npcs = 50). RPCA integration was performed to integrate the principal components (Seurat, IntegrateLayers) and used as input for *k* nearest-neighbour graph construction (Seurat, FindNeighbors, dims = 50) and Leiden clustering with a resolution of 2.5 (Seurat, FindClusters), initially deliberately overclustering the dataset. An initial dataset of 351,737 nuclei was subjected to quality control. Potential doublets were identified using the scDblFinder^64^ package. Given the defined cluster structure in our dataset, we used a cluster-based approach for doublet identification, estimating the standard 10x doublet rate of 0.8% per 1,000 nuclei. To remove non-RGC nuclei, we filtered the dataset for clusters with high expression of *RBPMS*, the main RGC marker gene. After subsampling, nuclei with abnormally high (greater than mean + 3 s.d.) gene counts, fewer than 2,300 gene counts, a doublet score of >0.5, as well as mitochondrial counts of >5% were removed.

#### Clustering and cell type annotation

After removing low-quality and non-RGC nuclei from the dataset, we repeated the normalization and clustering pipeline at a resolution of 0.5 (RunPCA, npcs = 30; FindNeighbors, dims = 30). For each cluster, we calculated differentially expressed marker genes compared with every other cluster (Seurat, FindMarkers). Clusters were merged if ≤5 differentially expressed genes were found between them, with an average log_2_-transformed fold change of >2 or <–2 and a *P* value < 0.05. Moreover, if a cluster contained fewer than 200 nuclei in the control condition or was absent in two or more samples, it was fused with its nearest neighbour based on a Euclidean distance matrix constructed in gene expression space (Seurat, BuildClusterTree; Supplementary Fig. 1e). The remaining 27 clusters were annotated manually based on their respective marker gene expression, using three published healthy human RGC datasets as reference^8–10^. To avoid disease-related transcriptional changes influencing cell type annotation, marker genes were identified exclusively from healthy control samples using Seurat’s FindAllMarkers function. Of the top 30 markers of each cluster, 2–3 are shown in Extended Data Fig. 1e. For each reference dataset, the top 50 marker genes per cell type were selected and used for gene set enrichment analysis (GSEA) through the ClusterProfiler^65^ package, applying the ranked cluster markers from the healthy control samples of our dataset. For the enrichment analysis in Extended Data Fig. 1c and the cell type marker identification in Extended Data Fig. 1e, the three midget OFF RGC subtypes (MG-OFF1, MG-OFF2, MG-OFF3) and the four midget ON RGC subtypes (MG-ON1, MG-ON2, MG-ON3, MG-ON4) were combined into single midget OFF and midget ON types. This consolidation streamlined marker gene identification and cell type annotation. The merged midget ON and midget OFF RGC clusters were also used for the UMAP representation in Fig. 1c.

#### Differential gene expression analysis

To analyse the transcriptional response of all RGCs during MS, we performed sample- and condition-wise pseudobulk aggregation of unnormalized counts (Seurat, aggregateExpression). The DESeq2 package^66^ (v.1.44) was used for normalization and differential gene expression analysis, considering genes with false discovery rate adjusted *P* value < 0.05 as differentially expressed. Enrichment analysis of biological themes was performed with the ClusterProfiler^65^ (v4.12.6) package.

#### Analysis of resilient and susceptible RGC subtypes

To assess differences in RGC vulnerability among cell types in MS, we calculated the relative frequency of each cell type in both control and MS RGC populations. Outliers were identified and excluded using Grubbs’ test. Significant differences in the relative frequencies between control and MS samples were assessed using Mann–Whitney *U*-tests. Clusters with significantly lower relative frequencies in MS compared with controls (*P* < 0.05, log_2_-transformed fold change < 0) were defined as susceptible. Clusters with a significantly higher relative frequency in MS compared with controls (*P* < 0.05, log_2_-transformed fold change > 0) were defined as resilient. Clusters falling into either term but not reaching significance (*P* > 0.05) were classified as intermediate susceptible (log_2_-transformed fold change < 0) or intermediate-resilient (log_2_-transformed fold change > 0). We performed sample- and condition-wise pseudobulk aggregation of significantly de-enriched (MG-ON4, MG-OFF2, ipRGC) and significantly enriched clusters (PG-ON, RGC26, RGC13) to compare transcriptional profiles between susceptible and resilient clusters. DESeq2 analysis was conducted to determine differentially expressed genes between resilient MS, resilient control, susceptible MS and susceptible control pseudobulks. Gene regulation modules were defined as follows: module A, genes upregulated in resilient RGCs compared to susceptible RGCs; module B, genes upregulated in susceptible RGCs compared with resilient RGCs; module C, genes upregulated in resilient RGCs during MS; module D, genes downregulated in resilient RGCs during MS; module E, genes upregulated in susceptible RGCs during MS; module F, genes downregulated in susceptible RGCs during MS. Gene Ontology analysis with the genes in each module was performed using the clusterProfiler^65^ package. To calculate resilience and vulnerability scores for the susceptible, intermediate susceptible, intermediate resilient and resilient pseudobulks of each donor and condition, we applied the AUCell algorithm^67^, using the top 200 module-defining genes from modules A–E as input, after pseudobulk aggregation and normalization of gene expression. To calculate intrinsic resilience signatures for each pseudobulked RGC subtype and donor, we used the top 200 genes of module A (intrinsic resilience signature) and module B (intrinsic susceptibility signature) as the input for AUCell analysis, after aggregation and normalization of gene expression by donor and cell type.

#### Linear regression analysis of intrinsic susceptibility and resilience genes

To identify genes of which the expression correlated with resilience, we extended the analysis to include all RGC types rather than focusing solely on the most resilient or susceptible clusters. Clusters were ranked from most susceptible to most resilient based on the signed negative logarithmic *P* value of their relative frequency in MS compared with controls. We focused on the ten samples from control individuals. To minimize biases arising from differences in cluster size across RGC types, we standardized cluster sizes by bootstrapping the dataset, randomly selecting 100 nuclei from each cluster within each sample. Clusters containing fewer than 10 nuclei were excluded. For clusters with fewer than 100 nuclei, sampling was performed with replacement; for larger clusters, it was performed without replacement. This process was repeated five times, and pseudobulk count vectors were summed across iterations. The DESeq2 package (v.1.44) was used to normalize gene counts of the size-adjusted pseudobulks, accounting for differences in sequencing depth and library size across samples. Variance stabilization was performed using the rlog function within DEseq2. For each gene, a linear regression was performed using the lm function in R (formula: normalized expression ~ resilience), modelling the normalized gene expression in a given cluster and sample as a function of the cluster resilience score, as defined previously by the signed negative logarithmic *P* value of a cluster’s relative frequency in MS compared with controls. *P* values for significance were adjusted for multiple comparisons using the FDR method.

#### Single-molecule fluorescence multiplex in situ RNA hybridization

smFISH was performed on representative post-mortem, unfixed MS and control cryosections (16 µm thickness) using the RNAscope Multiplex Fluorescent Detection Kit v2 (ACD Biotechne, 323100) according to the manufacturer’s protocol. The following human RNAscope assay probes were used (ACD Biotechne): POU4F1 (438441), TBR1 (425571-C2), FOXP2 (407261-C2), GPR149 (1040901-C3) and CFH (428731-C3). Probes were labelled with TSA Vivid Fluorophores (fluorescein, cyanine 3, cyanine 5, Akoya Biosciences). Each run included quality-control slides stained with the human 3-plex RNAscope positive-control probe (320861) and 3-plex negative-control probe (320871) from ACD Biotechne.

#### Image acquisition and quantification of fluorescence multiplex in situ RNA hybridization

Multiplex fluorescence images were acquired using the Leica DM6 B microscope equipped with a Leica K5 camera and the THUNDER imaging system for preprocessing. Images were captured at ×20 magnification as z stacks to ensure thorough signal capture within tissue sections. Acquisition was performed with LAS-X software, and images were exported as LIF files for subsequent analysis with QuPath^68^ (v.0.4.3). In QuPath, analysis was performed using the subcellular spot detection tool. The RGC layer was first selected, followed by cell detection based on the DAPI channel. Subcellular spot detection was then conducted for *POU4F1*, *FOXP2*, *TBR1*, *CFH* and *GPR149*. Double-positive cells (such as *POU4F1*-positive cells co-expressing specific subpopulation markers) were identified using an initial single-measurement classifier, followed by a composite classifier.

#### RT–qPCR

We reverse-transcribed RNA to complementary DNA using the RevertAid H Minus First Strand cDNA Synthesis Kit (Thermo Fisher Scientific, K1632) according to the manufacturer’s instructions. Samples were measured on the QuantStudio Flex Real-Time PCR System using TaqMan Gene Expression Assays (Thermo Fisher Scientific) for *Cfh* (mouse, Mm01299248_m1), *RBPMS* (human, Hs01060992_m1) and *RCVRN* (human, Hs00610056_m1). All analyses were performed in triplicate. We calculated gene expression as ${2}^{-\varDelta {C}_{{\rm{t}}}}$ relative to *Tbp* (mouse, Mm01277042_m1) or *GAPDH* (human, Hs99999905_m1) as the endogenous control.

#### Human histopathology of post-mortem tissues

Tissue paraffin sections were cut at 3 μm and stained with haematoxylin and eosin (H&E) according to standard procedures. For immunohistochemical staining, brain tissue sections were processed as follows. After dewaxing and inactivation of endogenous peroxidases (3% hydrogen peroxide), antibody-specific antigen retrieval was performed using the Ventana BenchMark XT autostainer. The sections were blocked with rabbit serum and then incubated with the primary antibody against human CFH (1:40; R&D, AF4779). For detection of specific binding, the anti-goat Histofine Simple Stain MAX PO immune-enzyme polymer (Nichirei, 414161F) was used as the secondary antibody. For DAB staining, the UltraView Universal DAB Detection Kit (Roche, 760-500) was used. Counterstaining and bluing were performed using haematoxylin (Ventana Roche, 760-2021) and Bluing Reagent (Ventana Roche, 760-2037) for 4 min. Subsequently, stained sections were mounted in mounting medium. For quantification, DAB-positive neurons were counted manually per area of cortical tissue within ImageJ (Fiji, v.2.14.0). Neurons were identified on the basis of their characteristic morphology in H&E-stained sections. Information about donors used for histopathology is provided in Supplementary Table 2.

### Animal models

#### Mice

All mice, including C57BL/6J WT (The Jackson Laboratory), SJL/JRj WT (Janvier Labs), C3 knockout (B6.129S4-*C3*^*tm1Crr*^/J, The Jackson Laboratory) and *Cfh*^*flox/flox*^ mice were maintained under specific pathogen-free conditions in the central animal facility of the University Medical Center Hamburg-Eppendorf (UKE). Adult mice aged 8–20 weeks were used for experiments. For EAE experiments involving C57BL/6J WT, SJL/JRj WT and *C3*-KO mice, only female animals were included. To assess potential sex dependent effects, an additional cohort of male C57BL/6J mice was used to compare neuronal mHDM-FH expression with a GFP control. For experiments with *Cfh*^*flox/flox*^ mice both sexes were used. The mice were kept under a 12 h light–12 h dark cycle at 22 ± 2 °C and 40–60% relative humidity with ad libitum access to standard chow (Altromin, 1328P) and water. EAE mice additionally received DietGel Recovery (Ssniff, H007-72065). For EAE experiments, if both sexes were included, equal numbers of male and female mice were allocated to each experimental group. Furthermore, animals from each litter were evenly distributed across groups to minimize potential litter effects. Within these constraints, mice were randomly assigned to receive either effector-AAVs or control AAVs. Sample sizes were selected based on the principles of the 3Rs (replacement, reduction and refinement), the use of genetically homogeneous inbred mouse strains and sample sizes commonly used in previous studies employing the EAE model that detected comparable biological effects^25,37,38^. Clinical EAE scoring was conducted in a blinded manner, with researchers unaware of both the genotype and the injected AAV to prevent observer bias.

#### Generation of *Cfh* neuronal knockout mice

*Cfh*^*tm1a(EUCOMM)Wtsi*^ sperm (The Jackson Laboratory) were revitalized on C57BL/6 wild-type female mice (The Jackson Laboratory), resulting in litters positive for the *Cfh*^*tm1a(EUCOMM)Wtsi*^ allele. To obtain the conditional (tm1c) allele (*Cfh*-flox), these animals were bred with ACTB:FLPe B6J mice expressing the Flp recombinase (The Jackson laboratory, 005703)^69^. To generate neuronal *Cfh-*KO mice, we performed retrobulbar intravenous injections of an AAV (serotype PhP.eB) carrying cre recombinase under the control of the h*SYN1* promoter in *Cfh*^*flox/flox*^ mice.

#### EAE model

For experiments involving AAV-mediated expression, mice received retrobulbar injections of 100 μl of the respective AAV (serotype PhP.eB) at a titre of 3 × 10^11^ viral genomes in PBS 3 weeks before EAE induction. Mice were monitored daily and weighed starting 1 week after the injection. Mice injected with effector constructs were compared to littermates receiving GFP-only control AAVs. To induce EAE in C57BL/6 mice, they were immunized subcutaneously with 200 μg MOG_35–55_ peptide (Schafer-N) emulsified in Complete Freund’s adjuvant (CFA) (Difco, DF0639-60-6) containing 2 mg ml^−1^ *Mycobacterium tuberculosis* (Difco, DF3114-33-8). Moreover, 300 ng of pertussis toxin (Calbiochem, CAS70323-44-3, 516562) was administered intraperitoneally on the day of immunization and again 48 h later. For experiments in SJL mice, EAE was induced by subcutaneous immunization with 100 µl of PLP_139–151_ peptide (1.5 mg ml^−1^) emulsified 1:1 with 100 µl of CFA (Difco, DF0639-60-6) containing *M. tuberculosis* H37RA (1 mg ml^−1^, 100 µg per mouse). Clinical signs were assessed daily using the following scoring system: 0, no clinical deficits; 1, tail weakness; 2, hind limb paresis; 3, partial hind limb paralysis; 3.5, full hind limb paralysis; 4, full hind limb paralysis with forelimb paresis; and 5, premorbid state or death. Animals reaching a clinical score of ≥4 were euthanized in accordance with the regulations of the German Animal Welfare Act. All EAE experiments were conducted with investigators blinded to the genotype and treatment conditions.

#### Visual assessment in mice

To evaluate visual acuity and contrast sensitivity in EAE mice, automated optomotor testing was performed using the OptoDrum system (Striatech)^70,71^. Mice were placed individually onto an elevated central platform surrounded by computer monitors and monitored from above by a camera. The optomotor reflex, a head-tracking movement elicited by rotating visual stimuli, was induced by a moving vertical black-and-white grating displayed on the surrounding screens. Visual acuity was determined by progressively increasing the spatial frequency (that is, the number of stripes per degree of visual angle) until the optomotor reflex could no longer be elicited. Contrast sensitivity was assessed by gradually reducing the contrast of the grating until the reflex was no longer observed. For each stimulus condition (spatial frequency or contrast level), two successful responses were required to advance to the next step, whereas three consecutive failures were defined as an unsuccessful trial.

#### Analysis of published EAE sequencing datasets

Raw count matrices and metadata from publicly available RNA-seq datasets were retrieved from the Gene Expression Omnibus (GEO) repository. The GSE118948 dataset includes single-cell sequencing data of CD45^+^ cells isolated from the spinal cords of EAE mice 15 days after immunization. Analysis of the single-cell data was performed using Seurat, with cell type annotations from the original publication^22^. Differential expression analysis was conducted with the FindMarkers function within Seurat using a Wilcoxon rank sum test to compare gene expression in healthy mice and those with acute EAE across T cells, neutrophils, dendritic cells, macrophages, monocytes, CNS-associated monocytes and microglia. Bulk RNA-seq data from GEO GSE194071 (ref. ^23^) were used to examine spinal cord microglia in acute and chronic recovery EAE mice, while GSE100329 (ref. ^24^) provided data for analysing spinal cord astrocytes in acute and chronic progressive EAE mice. Moreover, GSE104899 (ref. ^25^) and GSE279707 (ref. ^26^) were used to study spinal cord motor neurons in acute EAE mice. All bulk sequencing data were analysed using DESeq2 (v.1.44), and signed negative logarithmic FDR-adjusted *P* values were calculated for visualization.

#### Immunohistochemistry of spinal cord tissue

Mouse spinal cord tissue was obtained and processed as described previously^72^. Images were acquired using a Zeiss LSM 900 Airyscan 2 laser-scanning confocal microscope equipped with ZEN blue software v.3.9. Antibodies and their dilutions used for immunohistochemistry are specified in Supplementary Table 3.

#### Immunohistochemistry analysis of mouse retinal whole mounts

After transcardial perfusion of mice with ice-cold 4% PFA, the orientation of the eye was marked in situ, and eyes were enucleated and post-fixed in 4% PFA for 1 h. The eyeballs were then transferred to ice-cold PBS. Whole-mount dissection of retinas was performed under a dissection microscope. For immunohistochemistry, retinas were washed in PBS 0.5% Triton X-100 and permeabilized by freezing them at −70 °C for 15 min. After thawing, the retinas were rinsed with PBS 0.5% Triton X-100 and incubated overnight with primary antibodies at 4 °C in blocking buffer (PBS with 2% Triton X-100 and 2% normal donkey serum). The retinas were subsequently washed in PBS 0.5% Triton X-100 and incubated with secondary antibodies in PBS with 2% Triton X-100 for 2 h at room temperature. The retinas were washed with PBS and mounted vitreal side up. All antibodies used are listed in Supplementary Table 3. Retinal whole mounts were imaged using the Zeiss Axiocam 705 mono microscope. Semi-automated quantification of GFP-positive RGCs was performed within Fiji (ImageJ, v.2.14.0). Background subtraction was applied to correct for uneven illumination and contrast enhancement was performed to optimize illumination. Thresholding used the default autothreshold method and the image was then converted into a binary mask. To reduce noise, the Despeckle function was applied. Segmentation was refined with the Watershed algorithm to separate cells. Particle analysis was conducted on segmented objects with inclusion of particles with a size of 30–300 pixels and circularity range of 0.4–1.0.

#### Luxol fast blue staining

Myelination in EAE mice was assessed by Luxol Fast Blue staining in transverse sections of the dorsal columns in the cervical, thoracic and lumbar spinal cord as previously described^73^. The slides were imaged on the NanoZoomer 2.0-RS digital slide scanner with NDP.view2 software (Hamamatsu). A customized counting mask within Fiji (ImageJ, v.2.14.0) was created to quantify the area of Luxol Fast Blue-positive axons in the white matter of the spinal cord. The Luxol Fast Blue-positive area was normalized to the total analysed area within the dorsal column.

#### Immunoblot

Spinal cords of healthy and EAE mice were homogenized in 2 ml radioimmunoprecipitation buffer (50 mM Tris, 150 mM NaCl, 0.5 mM EDTA, 10% SDS, 1% NP-40, 10% sodium deoxycholate, with protease and phosphate inhibitor cocktails (cOmplete, Sigma-Aldrich, 11836170001)) using a tissue grinder and incubated at 4 °C for 30 min on a rotating wheel. The lysates were centrifuged for 5 min to remove the cell debris. Protein concentrations were determined by BCA assay (Pierce BCA Protein Assay Kit, Thermo Fisher Scientific, 23228 and 23224) according to the manufacturer’s protocol, and 25 µg of protein were loaded onto SDS–PAGE (NuPAGE, Thermo Fisher Scientific, NW04125BOX) followed by wet transfer to polyvinylidene fluoride membranes. Blocking was performed using 5% BSA for 1 h at room temperature. Membranes were incubated with primary antibodies overnight at 4 °C. Horseradish-peroxidase-coupled secondary antibodies were applied for 1 h at room temperature, and chemiluminescence was visualized using WesternSure PREMIUM Chemiluminescent Substrate (LI-COR, 926-95000) according to the manufacturer’s protocol. A list of all of the antibodies used is provided in Supplementary Table 3. All uncropped western blot membranes are provided in Supplementary Fig. 18.

#### Immune cell isolation for flow cytometry

Spinal cord tissue was collected after transcardial PBS perfusion and dissociated into single-cell suspensions using 1 mg ml^−1^ collagenase A (Roche, 11088793001) and 0.1 mg ml^−1^ DNase I from bovine pancreas (Merck Millipore, 260913) with the gentleMACS Octo Dissociator (Miltenyi Biotec, program: Multi_F). The dissociated tissue was passed through a 70 µm cell strainer, and immune and glial cells were enriched using a discontinuous density gradient. After centrifugation at 2,500 rpm, 4 °C for 30 min, cells were collected from the interphase between the 30% Percoll and 70% Percoll layers. Non-specific Fc-receptor-mediated antibody binding was blocked by pre-incubation with TruStain FcX anti-mouse CD16/32 antibody (BioLegend, 101320) for 10 min at 4 °C, followed by surface antibody staining in Brilliant Stain Buffer (BD Biosciences, 563794) for 30 min at 4 °C. Dead cells were excluded from analysis using Zombie Green Fixable Viability Stain (BioLegend, 423112). For intracellular staining of CD206 and CD68, cell suspensions were fixed for 20 min at room temperature using fixation buffer (BioLegend, 420801), followed by 20 min incubation with anti-CD68 antibody in intracellular staining permeabilization wash buffer (BioLegend, 421002). Absolute cell numbers for CD45^high^ leukocytes and CD45^med^ microglia were determined using Precision Count Beads (BioLegend, 424902). We obtained data using the BD Symphony A3 flow cytometer (BD Biosciences) and analysed data using FlowJo v.10.9 (BD Biosciences).

### Viral vectors

#### Vector construction

All primers and oligonucleotides used in this study are listed in Supplementary Table 4. For expression of synthetic minimal CFH-derived constructs, clonal genes were synthesized by Twist Bioscience (Supplementary Table 4 (gene synthesis tab)). In mHDM-FH, the N-terminal SCR1–5 are linked to C-terminal SCR18–20 through an SGSG linker. Amino acids 24–152 of mouse CFHR-1 were used as a dimerization unit to increase protein half-life. The amino acid sequence of mHDM-FH was derived from a previous study^31^. CR2-FH is a fusion protein consisting of a complement receptor 2 fragment linked to the N-terminal SCR domains 1–5 of CFH. The amino acid sequence of CR2-FH contains the signal peptide of mouse IgG κ light chain for optimal secretion, amino acid residues 1–257 of mature mouse CR2, a (G_4_S)_2_ linker, followed by the 5 N-terminal SCR domains of mouse factor H^33^. Both sequences were codon-optimized for expression in mouse cells. A Pfl23II restriction-enzyme-recognition site was attached to the 5′ end, and BamHI and SalI sites were added to the 3′ end. Clonal genes were digested with Pfl23II and SalI for ligation into a customized pAAV backbone with a h*SYN1* promoter derived from pAAV-hSYN1-mTurquoise2 (gift from V. Gradinaru, Addgene 99125), an eGFP:KASH cassette (AAV:ITR-U6-sgRNA(backbone)-hSyn-cre-2A-EGFP-KASH-WPRE-shortPA-ITR, gift from Feng Zhang, Addgene 60231) and a P2A-T2A cleavage peptide. A control plasmid containing a stop codon was cloned downstream of the P2A-T2A cleavage peptide. For lentiviral expression in primary neuronal cultures, clonal genes were digested with Pfl23II and BamHI for ligation into a custom lentiviral backbone with an h*SYN1* promoter, a mScarlet cassette and a P2A cleavage peptide. For lentiviral expression of full-length mouse CFH, two separate PCR fragments were generated and linked with a triple-ligation strategy. The full-length *Cfh* sequence was amplified from mouse kidney cDNA with primers containing Pfl23II and XbaI, or XbaI and SgsI restriction sites, respectively. The inserts were ligated into the same lentiviral backbone described above. These constructs were subsequently used as templates to generate different mHDM-FH and CFH variants using the primers in Supplementary Table 4. To create constructs lacking the signal peptide, forward primers excluding the first 54 base pairs (P15–P18) were used. To exclude SCR20, we used reverse primers complementary to SCR19 (P10–P12) and inserted the construct upstream of the dimerization domain. The 2×KDEL sequences for retention in the secretory pathway were included in the reverse primers (P19–P20). For expressing the SCR20 alone (P13–P14), SCR20 was added downstream of an Lck-transmembrane domain (P8–P9) for intracellular retention at the cell membrane. The Lck domain was amplified from PZac2.1 hSYN1-Lck-13×Linker-BioID2-BioID2-HA (gift from B. Khakh, Addgene 176854). The lentiviral IFNγ overexpression construct was generated as previously described^37^. We used an AAV carrying cre recombinase under the h*SYN1* promoter to generate conditional knockouts in neuronal cultures (pAAV-hSYN1-cre-P2A-dTomato, gift from R. Larsen (Addgene 107738). To generate conditional knockouts in mice, the plasmid pENN.AAV.hSYN.HI.eGFP-cre.WPRE.SV40, a gift from J. M. Wilson (Addgene 105540) was used. For expression of TurboID constructs, the sequence for the biotin ligase was amplified from pAAV-TBG-Cyto-TurboID (gift from J. Long, Addgene 149414) using primers for a C-terminal HA protein tag and inserted into a lentiviral backbone with a h*SYN1* promoter using NheI and BamHI (Turbo-Cyto; P21-P22) or Nhel and EcoRI (Turbo-ER; P23-P24). For ER retention of the TurboID-ER construct, we included an N-terminal signal peptide and a C-terminal KDEL sequence, separated by a GGGGS linker. For in vivo delivery, the TurboID-ER cassette was transferred into a pAAV backbone with a h*SYN1* promoter using NheI and SgsI (P25–P26). The following constructs were generated and used for in vitro or in vivo experiments in this study: Lenti-hS-mmCfh-P2a-mScarlet, Lenti-hS-mmCfhΔ20-P2a-mScarlet, Lenti-hS-mScarlet-P2a-mmCfh-2×KDEL, Lenti-hS-mmCfhΔSP-P2a-mScarlet, Lenti-hS-mmCfhΔ20ΔSP-P2a-mScarlet, Lenti-hS-mScarlet-P2a-CR2-FH, Lenti-hS-mHDM-FH-P2a-mScarlet, Lenti-hS-mHDM-FHΔ20-P2a-mScarlet, Lenti-hS-mScarlet-P2a-mHDM-FH-2xKDEL, Lenti-hS-mScarlet-P2a-mHDM-FHΔ20-2×KDEL, Lenti-hS-mHDMΔSP-FH-P2a-mScarlet, Lenti-hS-mHDM-FHΔ20ΔSP-P2a-mScarlet, Lenti-hS-Lck-scr20-P2a-mScarlet, Lenti-CMV-mmIfng-P2a-mScarlet, Lenti-hS-mScarlet, Lenti-CMV-mScarlet, Lenti-hS-TurboID-ER, Lenti-hS-TurboID-Cyto, pAAV-hS-eGFP(-KASH)-P2a(-T2a)-mHDM-FH, pAAV-hS-eGFP-P2a-mHDM-FHΔ20, pAAV-hS-eGFP-P2a-mHDM-FHΔSP, pAAV-hS-eGFP-P2a-Lck-scr20, pAAV-hS-eGFP(-KASH), pAAV-hS-eGFP-KASH-P2a-T2a-CR2-FH, pAAV-hS-TurboID-ER. All final products were confirmed by Sanger sequencing.

#### Lentiviral production

To produce VSV-G-pseudotyped lentiviruses, HEK293T cells were seeded out at 6 × 10^4^ per cm^2^ 1 day before transfection in DMEM containing glutamine and high glucose (Thermo Fisher Scientific), together with helper plasmids pMDLg/pRRE, pRSV-Rev and pMD2.G. pMDLg/pRRE (gift from D. Trono, Addgene 12251, 12253, 12259). For a 10 cm cell culture plate, we used 15 µg transfer plasmid, 10 µg pMDLg/pRRE, 5 µg pRSV-Rev and 2 µg pMD2.G. Plasmids were mixed in 1× HEPES-buffered saline (HBS) and 125 mM CaCl_2_ solution and applied to the HEK293T cells in the presence of 25 µM chloroquine diphosphate. After 12 h, the medium was changed and the supernatant was collected at 36 h after transfection, filtered through a 0.45 µm PES filter and immediately snap-frozen and stored at −80 °C. For CFH expression lentivirus production, lentiviral particles were concentrated using the Lenti-X Concentrator (Takara Bio, 631478) according to the manufacturer’s instructions. Primary neurons were transduced at 5–8 days in vitro (d.i.v.) with 70–80% efficiency, visually confirmed by fluorescent protein expression.

#### AAV production

For vector production, we selected the PhP.eB serotype, which has shown high efficiency in transducing the CNS^74^. AAV particles were produced according to the standard procedures of the UKE vector facility^38,75^.

### Cell culture

#### Primary neuronal cultures

For primary cortical cultures, pregnant C57BL/6J or *Cfh*^*flox/flox*^ mice were euthanized, and cortices were isolated and dissociated. Cells were plated at a density of 6 × 10^4^ cells per cm^2^ on poly-d-lysine-coated wells (5 µM, Sigma-Aldrich) and maintained in PNGM medium (Lonza, CC4461) at 37 °C, 5% CO_2_ and 98% relative humidity. Experiments were performed using cultures between 14 and 23 d.i.v. To generate *Cfh-*cKO neurons, neuronal cultures from *Cfh*^*flox/flox*^ mice were transduced at 4 d.i.v. with an AAV PhP.eB carrying cre recombinase (Addgene 107738) under the control of an h*SYN1* promoter at a multiplicity of transfection (MOI) of 30,000. The transduction efficiency, estimated visually based on eGFP expression, was 70–80%. Chronic IFNγ exposure was achieved by delivering a lentivirus containing an *Ifng* expression construct under the control of a CMV promoter at 7 d.i.v.^37^. Either mScarlet or GFP-only controls were used for all AAV and lentiviral expression experiments.

#### RNA-seq analysis of neuronal *Cfh-*cKO cultures

RNA from neuronal *Cfh-*cKO and control cultures with or without glutamate stimulation were isolated using the RNeasy Mini Kit (Qiagen). RNA-seq libraries were prepared using the TruSeq stranded mRNA Library Prep Kit (Illumina) according to the manufacturer’s instructions. After pooling, libraries were sequenced on the NovaSeq 6000 sequencer (Illumina) generating 150 bp paired-end reads. Reads were aligned to the mouse reference genome (mm10, 2020A) using STAR v.2.5.2b with the default parameters. Overlaps with annotated gene loci were counted using featureCounts v.1.5.1. Differential expression analysis was performed using DESeq2 (v.1.44), with genes showing a FDR-adjusted *P* < 0.05 considered differentially expressed. Gene lists were annotated using biomaRt (v.2.60.1). We then performed an AUCell (v.1.26) analysis to directly compare a transcriptional excitotoxicity signature between WT control, *Cfh-*cKO control, and glutamate-stimulated WT and *Cfh-*cKO neuronal cultures. The transcriptional excitotoxicity signature was defined as all differentially upregulated genes in our glutamate-stimulated WT neuronal cultures in comparison to the WT control condition. Moreover, we performed GSEA with the top 200 glutamate-induced genes in the *Cfh*-cKO glutamate versus WT glutamate condition.

#### Pfa1 and HEK293T cell lines

Immortalized mouse fibroblasts (Pfa1 cells) were received from the originating laboratory (Conrad laboratory, Helmholtz Institute)^41,76^ and were not further authenticated. HEK293T cells were purchased from an authorized vendor (ACC 635, DMSZ). Pfa1 cells and HEK293T cells were maintained in DMEM high glucose (4.5 g l^−1^ glucose, 21969-035, Gibco) supplemented with 10% FBS, 2 mM l-glutamine and 1% penicillin–streptomycin at 37 °C with 5% CO_2_. Cells were passaged after reaching approximately 80% confluency and were routinely tested for mycoplasma contamination. For experiments, they were seeded at 30,000 cells per cm^2^ 1 day before stimulation. The respective compounds and stimulation times are specified in the corresponding figure legends. All cell lines were regularly checked for mycoplasma contamination using the VenorGeM Advance kit (Minerva biolabs, 11-7024) according to the manufacturer’s instructions. Cell lines were free of mycoplasma contamination.

#### Cell viability assay

The RealTime-Glo MT Cell Viability Substrate and NanoLuc Enzyme (Promega, G9711) were combined, added to neuronal cultures and incubated for 5 h to allow equilibration of the luminescence signal before treatment application. Toxicity was assessed after exposure to 50 μM glutamate, 100 nM MDA (Sigma-Aldrich, 63287) or 6 µM RSL3 (Sigma-Aldrich, SML2234). Luminescence was recorded immediately before the stressors were applied and 15 h later using the Spark 10 M multimode microplate reader (Tecan) at 37 °C and 5% CO_2_. At least four technical replicates were included for each condition; each well’s datapoints were normalized to its final luminescence value before stressor application. Subsequently, the data were normalized to the mean of the control wells at each timepoint to account for well-to-well variability in cell seeding and to the maximal neuronal death (2 mM glutamate) condition. Statistical comparisons were performed using end-point measurements.

For the medium change, CFH antibody and IFNγ experiments, we measured cell viability by quantifying condensed nuclear signal. Neuronal cultures were exposed to 50 µM glutamate, 100 nM MDA or 6 µM RSL3 for 10 h. Where indicated, additional treatments were applied 30 min before the stressors. Hoechst 33342, a cell-permeant nuclear counterstain, was added to the culture wells at a final concentration of 20 µM and incubated for 1 h. After incubation, cells were washed twice with preconditioned medium before imaging. Image acquisition was performed using the Zeiss LSM 900 Airyscan 2 confocal microscope with a ×40 magnification objective. Using Fiji, we generated a mask to capture all nuclei and measured the Hoechst 33342 fluorescence intensity. We then defined condensed nuclei using a threshold corresponding to the mean signal of the control conditions plus 2.5 s.d. This was used to estimate the number of dying and surviving neurons after the respective stimuli were applied.

#### ROS imaging

ROS levels in response to glutamate, MDA and RSL3-induced stress were measured using CellROX Green Reagent (Thermo Fisher Scientific, C10444). Neuronal cultures were stimulated with 50 μM glutamate for 2 h, 100 nM MDA for 10 h or 6 µM RSL3 for 10 h. CellROX Green was added to each well at a final concentration of 5 μM after 30 min of stressor exposure, alongside Hoechst 33342, as a nuclear counterstain for visualization. After the incubation, cells were washed twice with preconditioned medium to remove excess reagents and imaged using the Zeiss LSM 900 Airyscan 2 confocal microscope at ×20 magnification. Nuclear fluorescence of the CellROX signal was quantified using Fiji (ImageJ, v.2.14.0) to assess ROS levels.

#### BODIPY C11 imaging

To assess lipid peroxidation, the BODIPY C11 probe (Image-iT, Thermo Fisher Scientific, D3861) was used. Neuronal cultures were exposed to 50 µM glutamate, 100 nM MDA or 6 µM RSL3 for 10 h. Where indicated, additional treatments were applied 30 min before the stressors. BODIPY C11 was added to the culture wells at a final concentration of 20 µM and incubated for 1 h. Simultaneously, Hoechst 33342, a cell-permeant nuclear counterstain, was included. After incubation, cells were washed twice with pre-conditioned medium before imaging. Image acquisition was performed using the Zeiss LSM 900 Airyscan 2 confocal microscope with a ×40 magnification objective. Lipid peroxidation was quantified using Fiji (ImageJ, v.2.14.0) software. In lentiviral expression experiments, the ratio of oxidized to non-oxidized lipids was calculated due to the negligible or undetectable mScarlet signal from lentiviral transduction under the microscopy settings used. Owing to the stronger tdTomato signal emitted after AAV transduction of our pAAV-hSYN1-cre-P2A-dTomato constructs, only the MFI of the oxidized lipids was quantified in these experiments. For time-resolved and subcellularly resolved lipid peroxidation analyses, we used the following live cell imaging dyes: 1 µM MitoTracker Deep Red FM (Thermo Fisher Scientific), 1 µM ER-Tracker Blue-White DPX (Thermo Fisher Scientific), 500 nM SiR-lysosome kit (Cytoskeleton), 10 µg ml^−1^ Hoechst 33342 (Thermo Fisher Scientific) and 500 nM SiR700 actine kit (Cytoskeleton). Dyes were applied in the indicated concentrations and washed out together with the BODIPY C11 probe. All imaging experiments, except for the ER-Tracker, included Hoechst 33342 staining. The oxidized BODIPY C11 signal in the overlap with the respective cell organelle marker was quantified.

#### Quantification of complement component levels by ELISA

Peripheral blood was collected from the vena cava at the time of euthanasia. The samples were centrifuged at 1,000*g* for 10 min at 4 °C, and plasma was stored at −80 °C until use. Spinal cord tissue was collected after perfusion with ice-cold PBS and snap-frozen in liquid nitrogen. Tissues were lysed in protein extraction buffer (100 mM Tris-HCl, pH 7.4, 150 mM NaCl, 1 mM EGTA, 1 mM EDTA, 1% Triton X-100, 0.5% sodium deoxycholate) supplemented with phosphatase and protease inhibitor cocktails and PMSF (final concentration 1 mM) added immediately before use. ELISAs were performed according to the manufacturers’ instructions. CFH levels were determined using the Mouse Complement Factor H ELISA Kit (Hycultec, RD-CFH-Mu); plasma samples were diluted 1:100,000 in PBS, and cell culture supernatants were used undiluted. Total C3 concentrations were measured using the Mouse C3 ELISA Kit (Abcam, ab263884); plasma was diluted 1:100,000 in sample diluent NS, and tissue lysates were diluted 1:1,000 in 1× cell extraction buffer PTR. C3a levels were quantified using the Mouse C3a ELISA Kit (MyBioSource, MBS2701721); plasma was diluted 1:500 in PBS, and tissue lysates were diluted 1:20 in reagent diluent. C5a concentrations were measured using the Mouse C5a DuoSet ELISA Kit (R&D Systems, DY2150); plasma was diluted 1:100 in PBS, and tissue lysates were diluted 1:10 in reagent diluent. To assess complement activation, C3 consumption was calculated by dividing total C3a concentrations by total C3 concentrations for each individual sample (C3a/C3 ratio). Standard curves for all ELISAs were generated using the provided recombinant standards and fitted with a four-parameter logistic regression model.

#### Immunocytochemistry

Neuronal cultures were grown on 12-mm-diameter coverslips and stimulated with 50 μM glutamate, 100 nM MDA or 6 µM RSL3 for 6 h. After stimulation, cells were fixed in 4% PFA for 15 min and blocked in 10% normal donkey serum containing 0.1% Triton X-100. Immunolabelling was performed using the specified antibodies (details are provided in Supplementary Table 3). Images were captured using the Zeiss LSM900 Airyscan 2 laser-scanning confocal microscope. Co-localization was analysed using Fiji (ImageJ, v.2.14.0). The percentage of overlap with CFH (from total CFH within the cell) was reported.

#### PLA between CFH and MDA-modified proteins

To investigate the interaction between CFH and MDA-modified proteins, we performed a Duolink in situ proximity ligation assay (PLA) according to the manufacturer’s instructions (Merck). In brief, PLA technology uses pairs of primary antibodies conjugated to complementary oligonucleotides that, when bound within around 40 nm of each other, enable rolling-circle amplification and generate a discrete fluorescence signal after hybridization with labelled probes. For detection of CFH–MDA interactions, a *Cfh*-KO-validated goat anti-CFH antibody (Quidel, A313, 1:200) was combined with the PLA Goat MINUS probe (Merck, DUO92006), and a rabbit anti-MDA-modified protein antibody (Abcam, ab27642, 1:200) was paired with the PLA Rabbit PLUS probe (Merck, DUO92002). Signal amplification was achieved using the Duolink in situ detection reagent, far-red channel (Merck, DUO92013). To identify neurons, actin co-staining was performed in parallel. Images were acquired using the Zeiss LSM900 Airyscan 2 laser-scanning confocal microscope. Puncta per neuron were counted and quantified.

#### Proximity labelling with TurboID

Proximity-dependent biotinylation was performed using the biotin ligase TurboID targeted either to the ER lumen or to the cytoplasm. Cytosolic TurboID (TurboID-Cyto) expresses an HA-tagged TurboID biotin ligase under the h*SYN1* promoter for cytosolic localization. ER-TurboID contains an HA-tagged TurboID (TurboID-ER) with an N-terminal signal peptide (SP) and a C-terminal KDEL ER-retention motif under the h*SYN1* promoter. Cells were transduced with lentiviruses expressing either cytoplasmatic or ER-targeted TurboID at d.i.v. 7. Proximity labelling was induced by supplementing the cell culture medium with 50 µM biotin (IBA Lifescience, 6-6325-001) for 30 min at 37 °C. Cells were then rapidly cooled on ice, washed with ice-cold PBS and lysed in denaturing lysis buffer (8 M urea, 10 mM Tris buffer, 100 mM NaH_2_PO_4_). The lysates were incubated with streptavidin-coated magnetic beads (Thermo Fisher Scientific, 88817) overnight, washed twice with RIPA buffer, once with 1 M KCl, once with 0.1 M Na_2_CO_3_, once with 2 M urea in 10 mM Tris-HCl (pH 8.0) and twice again with RIPA buffer. Bound proteins were eluted by boiling in SDS sample buffer at 95 °C for 5 min for immunoblot analysis. For in vivo proximity labelling, mice received retrobulbar injections of 100 μl of an AAV carrying ER-retained TurboID (serotype PhP.eB) at a titre of 3 × 10^11^ viral genomes in PBS 3 weeks before EAE induction. For biotin pulsing, chow was soaked with 2.5 mg biotin (IBA Lifescience, 6-6325-001) per mouse and day dissolved in water starting 3 days before termination. At the day of perfusion, mice received an i.p. injection of 2.5 mg biotin dissolved in 100 µl PBS in addition. Spinal cord tissue was collected after transcardial perfusion with 10 ml ice-cold PBS. Tissues were homogenized in lysis buffer using a tissue disruptor (twice for 1 min at 4 °C with intermittent cooling on ice) and lysates were cleared by centrifugation (20,000*g*, 30 min, 4 °C). Supernatants were incubated overnight at 4 °C with equilibrated streptavidin magnetic beads (100 µl per sample) under rotation. Beads were subsequently washed twice with RIPA buffer, once with 1 M KCl, once with 0.1 M Na_2_CO_3_, once with 2 M urea in 10 mM Tris-HCl (pH 8.0), and twice again with RIPA buffer. Bound proteins were eluted in 2× reducing sample buffer by heating at 95 °C for 5 min before downstream analysis.

#### LC–MS/MS based proteomics

Samples were boiled at 95 °C for 5 min and sonicated with a probe sonicator. The digestion was performed in a 96-well LoBind plate (Eppendorf) semi-automated on the Andrew+ Pipetting Robot (Waters). Disulfide bonds were reduced in 10 mM dithiothreitol for 30 min at 56 °C while shaking at 800 rpm and alkylated in presence of 20 mM iodoacetamide for 30 min at 37 °C. Carboxylate-modified magnetic E3 and E7 speed beads (Cytvia Sera-Mag) at a 1:1 ratio in liquid chromatography–mass spectrometry (LC–MS)-grade water were added in a 10:1 (beads/protein) ratio adapted from the SP3-protocol workflow^77^. Protein binding was performed at 50% acetonitrile during shaking at 600 rpm for 18 min. Magnetic beads were washed twice with 80% ethanol and 100% acetonitrile. Digestion with trypsin in 100 mM AmBiCa was performed (sequencing grade, Promega) at a 1:100 (enzyme:protein) ratio at 37 °C overnight while shaking at 500 rpm. Trifluoroacetic acid was added to a final concentration of 1% and shaken at 500 rpm for 5 min. The supernatant containing tryptic peptides was transferred into a new 96-well LoBind plate, ready for subsequent LC–MS/MS analysis. Chromatography separation of tryptic peptides was achieved with a two-buffer system (buffer A: 0.1% formic acid in H_2_O, buffer B: 0.1% formic acid in 80% acetonitrile) on a UHPLC (Vanquish neo UHPLC system, Thermo Fisher Scientific) at a flow rate of 0.4 µl min^−1^. Attached to the UHPLC was a peptide trap (300 µm × 5 mm, C18, PepMap Neo Trap Cartridge, Thermo Fisher Scientific) for online desalting and purification, followed by a 25 cm C18 reversed-phase column (120 Å, 1.7 µm, 75 µm × 250 mm, Aurora Ultimate, IonOptics). Peptides were separated using a 70 min method with linearly increasing acetonitrile concentration from 3% to 34% buffer B over 60 min. MS/MS measurements were performed on a quadrupole-orbitrap hybrid mass spectrometer (Exploris 480, Thermo Fisher Scientific). Eluting peptides were ionized using a nano-electrospray ionization source (nano-ESI) with a spray voltage of 1,700 and analysed in data-independent acquisition (DIA) mode. For each MS1 scan, the ion-accumulation time was set to automatic, with an AGC target of 300%. The scan was set to *m*/*z* 400–800 with a resolution of 120,000 at *m*/*z* 200. Within a precursor mass range of *m*/*z* 400–800, fragmentation in DIA mode with *m*/*z* 12 isolation windows and *m*/*z* 1 window overlaps was performed (total of 33 scan events). Fragmentation was performed at a normalized collision energy of 30% using higher-energy collisional dissociation. The Orbitrap resolution was set to 60,000. LC–MS/MS data were searched using the CHIMERYS algorithm integrated into the Proteome Discoverer software (v.3.1.0.638, Thermo Fisher Scientific) with the Inferys 3.0.0 as the prediction model against a reviewed *Mus musculus* Swissprot database. Carbamidomethylation was set as a fixed modification for cysteine residues. The oxidation of methionine was allowed as variable modification. A maximum number of one missing tryptic cleavage was set. Peptides between 7 and 30 amino acids were considered. A strict cut-off (FDR < 0.01) was set for peptide and protein identification.

#### Ethics

All experimental procedures involving animals complied with institutional guidelines and adhered to the German Animal Welfare Act. Ethical approval for animal experiments was granted by the State Authority of Hamburg, Germany (approval numbers 41/22, 53/24, 40/25). For human eye tissue obtained from the Johns Hopkins University, all procedures were approved within the IRB00374036 “Characterizing mechanisms involved in pathological processes of retina neurodegeneration and optic neuritis present in MS in post-mortem samples”. For the human tissue analyses, as the samples could no longer be linked to identifiable individuals, the work did not qualify as a “research project on humans” under § 9 para. 2 of the Hamburg Chamber of Commerce Act for the Health Professions. Consequently, consultation required under § 15 para. 1 of the Professional Code of Conduct for Physicians in Hamburg was not necessary. For cortex tissue obtained from the UK Multiple Sclerosis Tissue Bank at Imperial College London, all procedures used by the Tissue Bank in the procurement, storage and distribution of tissue have been approved by the relevant Multicentre Research Ethics Committee (08/MRE09/31+5).

#### Statistical analysis

All statistical analysis was performed in R (v.4.4.1). The statistical analyses used for snRNA-seq data are described in the respective sections of the Article. A detailed description of statistical methods for experimental data is provided in the figure legends. Unless stated otherwise, data are presented as mean values. Differences between two experimental groups were determined using unpaired two-tailed Student’s *t*-tests or Mann–Whitney *U*-tests. The reported *n* represents biologically independent samples and not technical replicates. Statistical analysis of the clinical scores in EAE experiments was performed using the Mann–Whitney *U*-test on the areas under the curve for each animal.

### Reporting summary

Further information on research design is available in the Nature Portfolio Reporting Summary linked to this article.

## Online content

Any methods, additional references, Nature Portfolio reporting summaries, source data, extended data, supplementary information, acknowledgements, peer review information; details of author contributions and competing interests; and statements of data and code availability are available at https://doi.org/10.1038/s41586-026-10981-y.

## Supplementary information

Supplementary Information Supplementary Figs. 1–18.

Reporting Summary

Supplementary Table 1 Summary table with information about control and MS donors used for retinal snRNA-seq, immunohistochemistry, qPCR and smFiSH.

Supplementary Table 2 Summary table with information about control and MS donors used for brain histopathology.

Supplementary Table 3 Summary table with information about the antibodies and their application used for immunohistochemistry, immunoblotting, flow cytometry, immunoprecipitation and neutralizing experiments.

Supplementary Table 4 Summary table with information about the primers and oligonucleotides used for cloning and vector construction.

Supplementary Data Source data for the Supplementary Figures.

## Source data

Source Data Fig. 1

Source Data Fig. 2

Source Data Fig. 3

Source Data Fig. 4

Source Data Extended Data Fig. 1

Source Data Extended Data Fig. 2

Source Data Extended Data Fig. 3

Source Data Extended Data Fig. 4

Source Data Extended Data Fig. 5

Source Data Extended Data Fig. 6

Source Data Extended Data Fig. 7

Source Data Extended Data Fig. 8

Source Data Extended Data Fig. 9

Source Data Extended Data Fig. 10

Source Data Extended Data Fig. 11

## Data Availability

Raw human retina snRNA-seq data have been deposited in the European Nucleotide Archive under accession number PRJEB102624. Processed snRNA-seq data, including the raw count matrix after quality control and corresponding cell- and sample-level metadata, are available at Figshare^78^ (https://doi.org/10.6084/m9.figshare.33075182). Sequencing reads were aligned using the 10x Genomics Cell Ranger GRCh38 reference (2020-A; https://www.10xgenomics.com/support/software/cell-ranger/downloads/cr-ref-build-steps#human-ref-2020-a). Raw bulk mRNA-seq data are available from the GEO under accession number GSE332759. Mouse sequencing reads were aligned using the 10x Genomics Cell Ranger mm10 reference (2020-A; https://www.10xgenomics.com/support/software/cell-ranger/downloads/cr-ref-build-steps#mouse-ref-2020-a). The MS proteomics data have been deposited to the ProteomeXchange Consortium through the PRIDE^79^ partner repository under dataset identifier PXD080433. Source data are provided with this paper.

## Code availability

The code used for the study is available from GitHub (https://github.com/Christina-Valeska/neuronal-intracellular-cfh.git).
