# Supplementary material for: Intracellular complement factor H protects neurons during CNS inflammation
Source: Nature. 2026 Sep 2;658(8136):765–73. doi: 10.1038/s41586-026-10981-y (PMC13668678; doi:10.1038/s41586-026-10981-y)

nature portfolio

https://doi.org/10.1038/s41586-026-10981-y

Supplementary information

Intracellular complement factor H protects neurons during CNS inflammation

In the format provided by the authors and unedited

Supplementary information

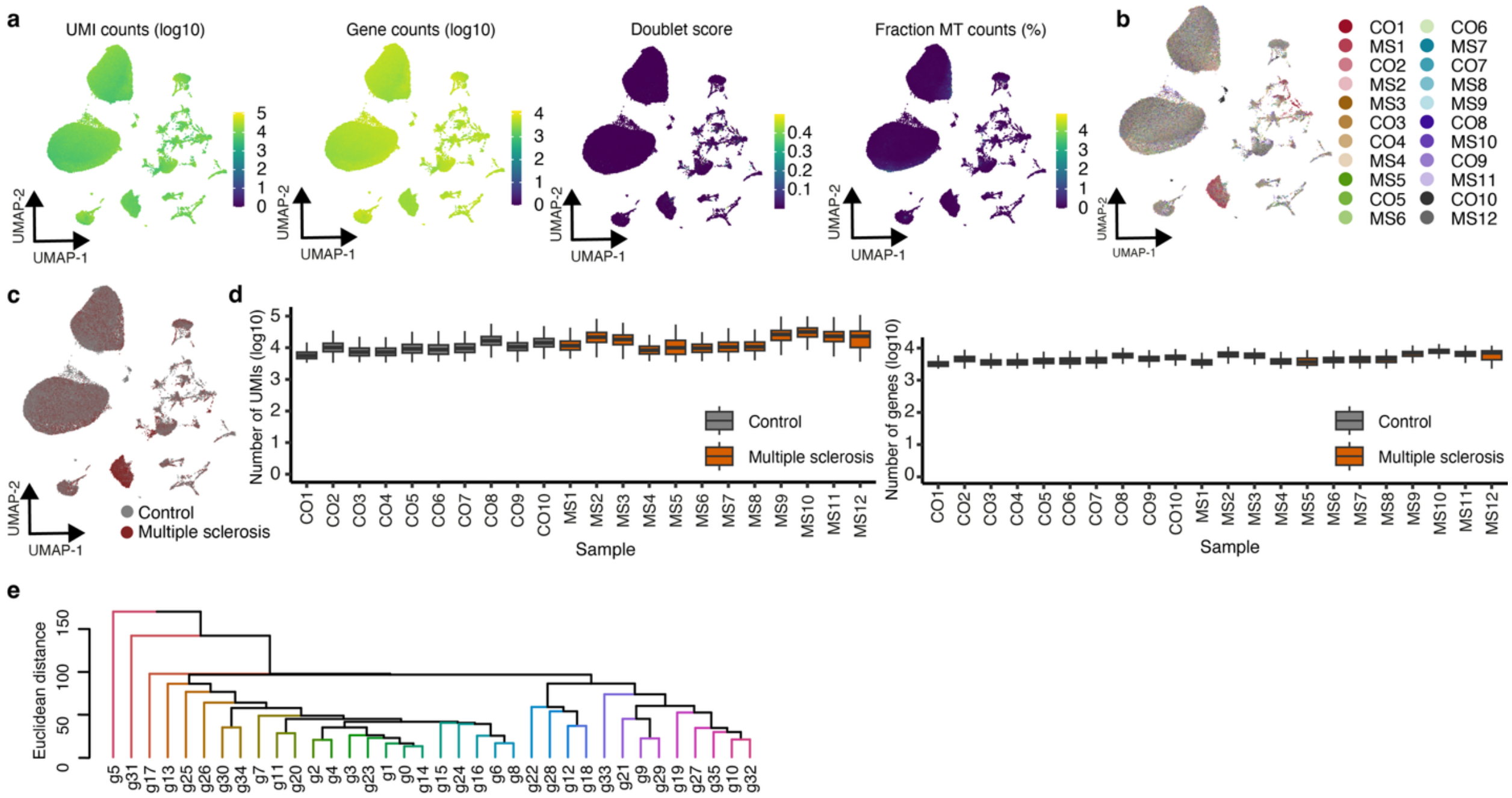

Supplementary Fig.1 | Human RGC snRNA-seq quality control. a, UMAP embedding of the RGC data set coloured by UMI counts, gene counts, doublet score, and fraction of mitochondrial (MT) gene counts. **b**,**c** UMAP embeddings coloured by sample (**b**) and condition (**c**). **d**, Distribution of unique molecular identifiers (UMIs) per nucleus (left) and genes detected per nucleus (right) in each sample. Boxes indicate the interquartile range (IQR), centre lines the median, and whiskers the most extreme values within 1.5 × IQR; outliers are omitted. **e**, Phylogenetic-like tree showing transcriptionally similar clusters based on principal component analysis. Each node represents a cluster before merging, and branch length reflects transcriptional distance between clusters.

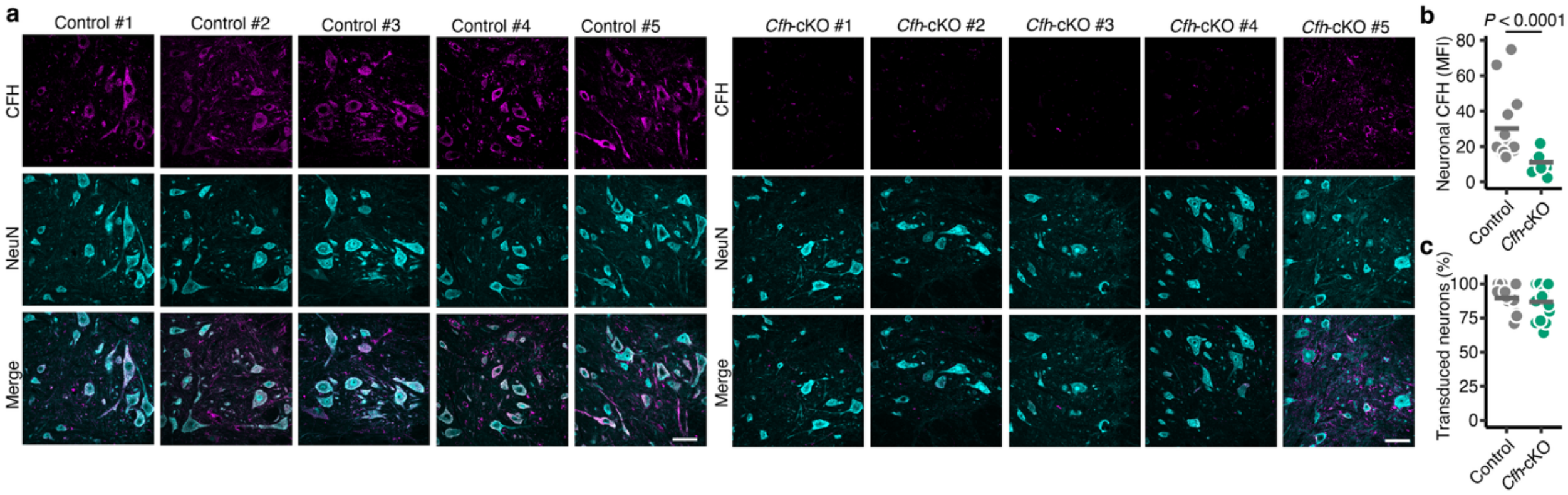

Supplementary Fig.2 | Neuronal CFH deficiency increases disease severity in EAE. a, Representative images of the spinal cord ventral horn at chronic EAE (day 26 post immunisation) from 5 *Cfh*$^{flox/flox}$ mice injected with AAV-hSyn1-GFP (control) or AAV-hSyn1-Cre to generate neuron-specific *Cfh* knockout (*Cfh*-KO). Neurons were identified by NeuN immunostaining. Scale bar, 50 µm. **b**, Neuronal CFH mean fluorescence intensity (MFI) in control (n = 13) and *Cfh*-cKO mice (n = 14). **c**, Transduction efficiency, calculated as the percentage of GFP-positive neurons in the ventral horn of control (n = 13) and *Cfh*-cKO (n = 14) mice. Individual data points represent biological replicates; bars indicate the mean. Statistical comparisons used two-sided unpaired Mann-Whitney *U* tests.

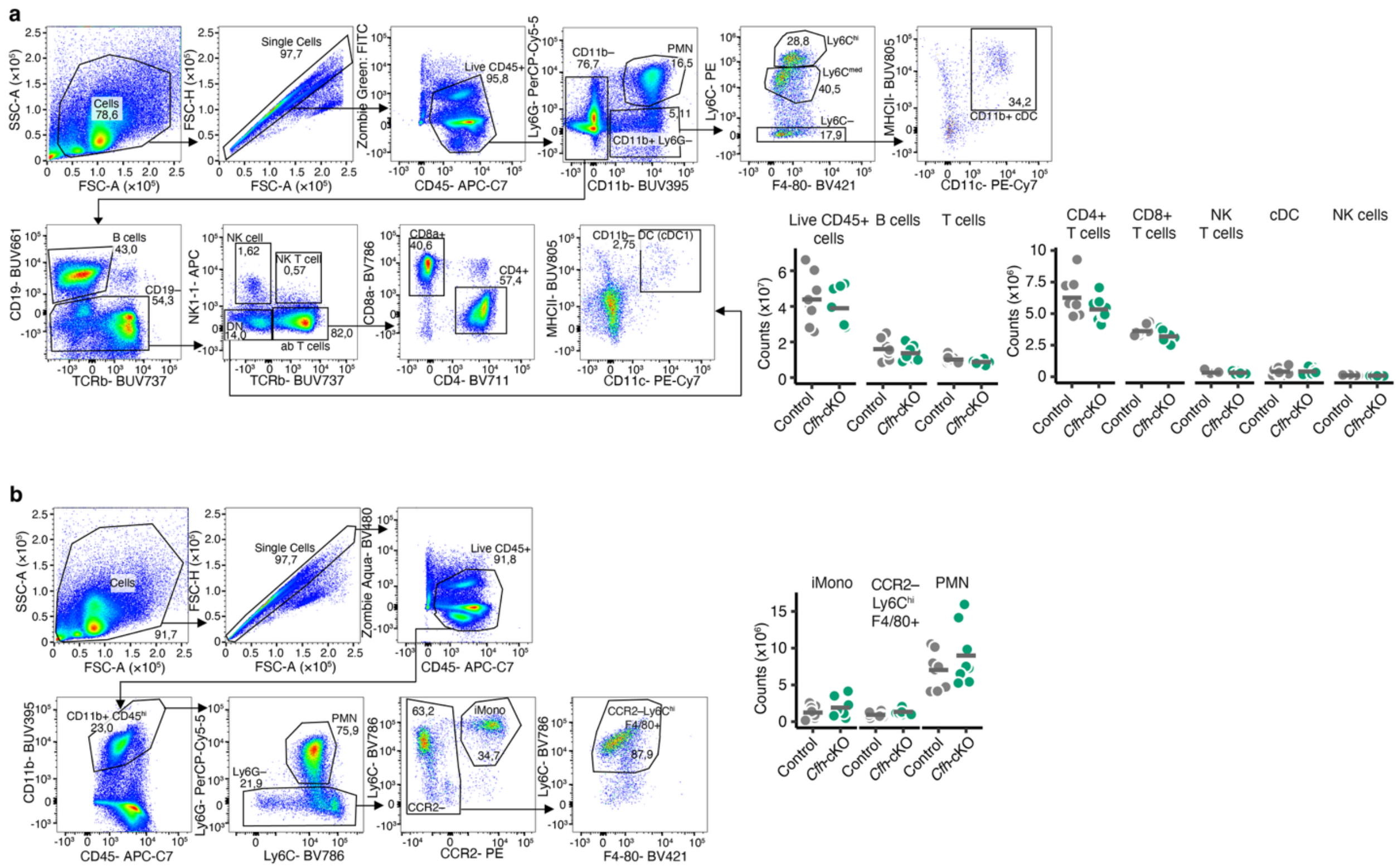

Supplementary Fig.3 | Peripheral immune cell characterisation in *Cfh*-cKO and control mice during acute EAE. **a**, Representative gating strategy and quantification of live CD45+ cells, B cells, T cells, CD4+ T cells, CD8+ T cells, NK T cells, conventional dendritic cells (cDCs), and NK cells in the spleen of control (*n* = 8) and *Cfh*-cKO mice (*n* = 8) during acute EAE (day 15 post-immunisation). **b**, Representative gating strategy and quantification of inflammatory monocytes (iMono; $CCR2^{+}Ly6C^{hi}F4/80^{+}$), $CCR2^{-}Ly6C^{hi}F4/80^{+}$ macrophages, and polymorphonuclear leukocytes (PMNs) in the same mice as shown in **a**. Individual data points represent biological replicates; bars indicate the mean. Statistical comparison used unpaired two-sided Mann-Whitney *U* tests. DN, double negative.

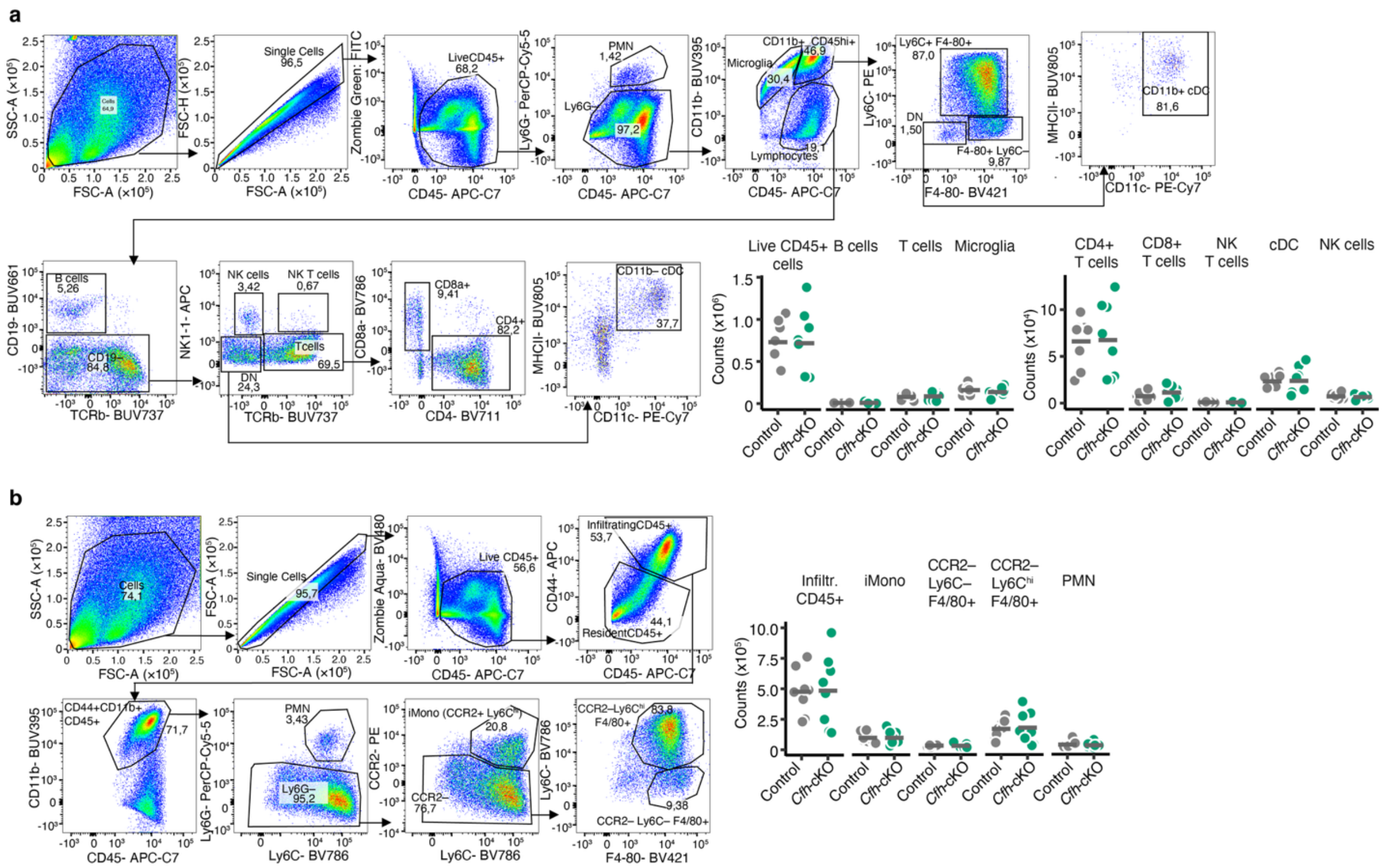

Supplementary Fig.4 l Immune cell characterisation in the spinal cord of *Cfh*-cKO and control mice during acute EAE. a, Representative gating strategy and quantification of live CD45⁺ cells, B cells, T cells, microglia, CD4⁺ T cells, CD8⁺ T cells, NK T cells, cDCs and NK cells in the spinal cord of the same mice as in Supplementary Data Fig.3. **b**, Representative gating strategy and quantification of infiltrating CD45⁺ cells, inflammatory monocytes (iMono; CCR2⁺Ly6C^hi^F4/80⁺), CCR2⁻Ly6C^hi^F4/80⁺ macrophages, Ly6C^hi^F4/80⁺ macrophages and polymorphonuclear leukocytes (PMN) in the same mice shown in **a**. Individual data points represent biological replicates; bars indicate the mean. Statistical comparison used unpaired two-sided Mann-Whitney *U* tests. DN, double negative.

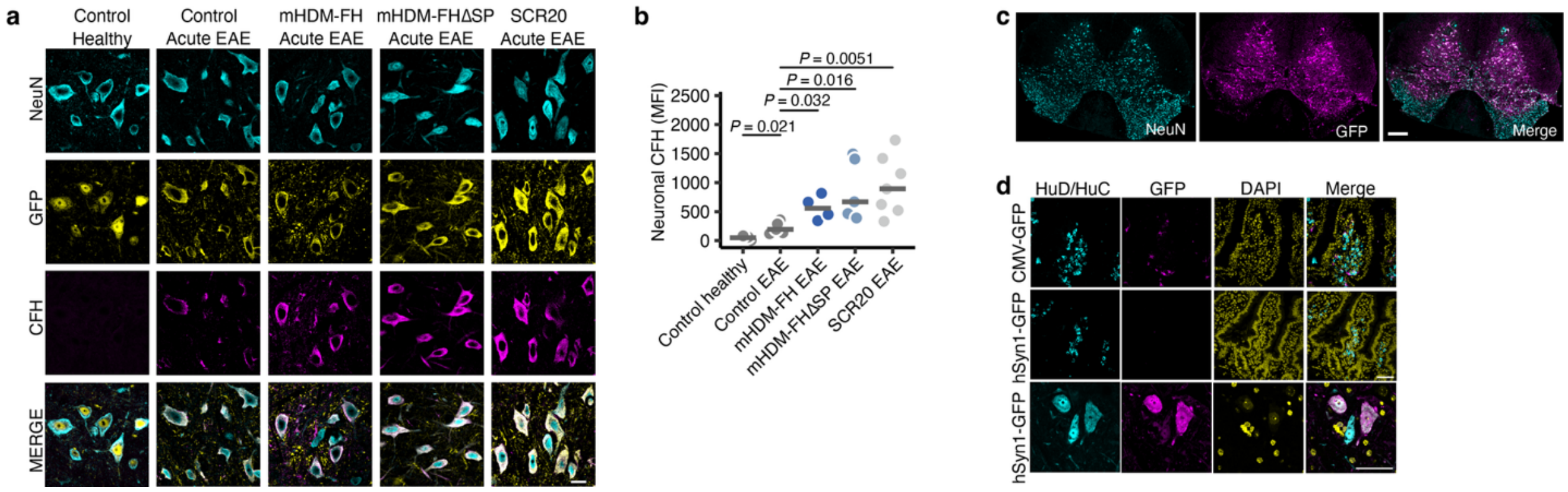

Supplementary Fig.5 | Neuronal mHDM-FH expression protects against neurodegeneration in EAE. a,**b**, Representative CFH immunohistochemistry (**a**) and quantification of neuronal CFH mean fluorescence intensity (MFI) (**b**) in the spinal cord of EAE mice at day 15 post-immunisation following AAV-mediated neuronal expression of GFP (n = 5), mHDM-FH (n = 4), mHDM-FHΔSP (n = 5), or SCR20 (n = 7). Unimmunised mice expressing neuronal GFP served as controls (n = 4). Scale bar, 20 µm. **c**, Representative spinal cord section from a healthy mouse expressing neuronal GFP under the *hSyn1* promotor. Scale bar, 200 µm. **d**, Representative GFP expression in the gut of mice expressing GFP under the CMV promoter (top) or the neuron-specific *hSyn1* promoter (middle). The bottom panel shows spinal cord motor neurons from the same *hSyn1*-GFP-expressing mouse, confirming successful neuronal transduction. Neurons were identified by HuD/HuC immunostaining. Individual data points represent biological replicates; bars indicate the mean. Statistical comparison used FDR-corrected two-sided unpaired Mann-Whitney *U* tests against acute GFP expression.

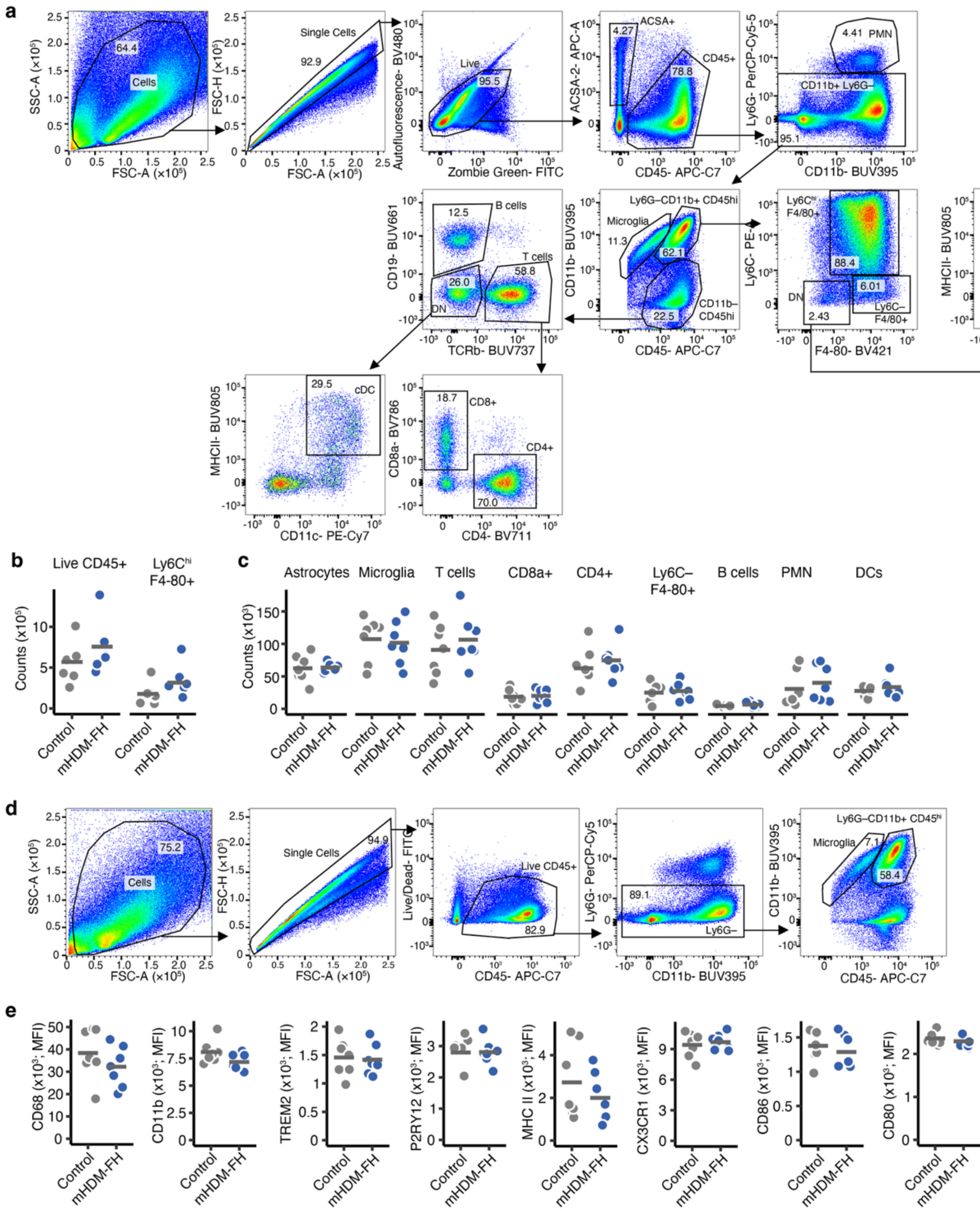

Supplementary Fig.6 | Unchanged immune response in acute EAE following neuronal mHDM-FH expression. **a–c**, Representative gating strategy (**a**) and absolute counts (**b**,**c**) of the indicated immune cell populations in the spinal cords of day 15 post-immunisation EAE mice expressing neuronal GFP (control, $n = 7$), or mHDM-FH ($n = 7$), measured by flow cytometry. **d**,**e**, Representative gating strategy (**d**) and median fluorescence intensity (MFI) of microglial activation markers (**e**) in the same mice. Percentage of cells are indicated within each gate. Individual data points represent biological replicates; bars indicate the mean. Statistical comparisons used two-sided unpaired Mann-Whitney *U* test. DN, double negative. PMN, polymorphonuclear neutrophils.

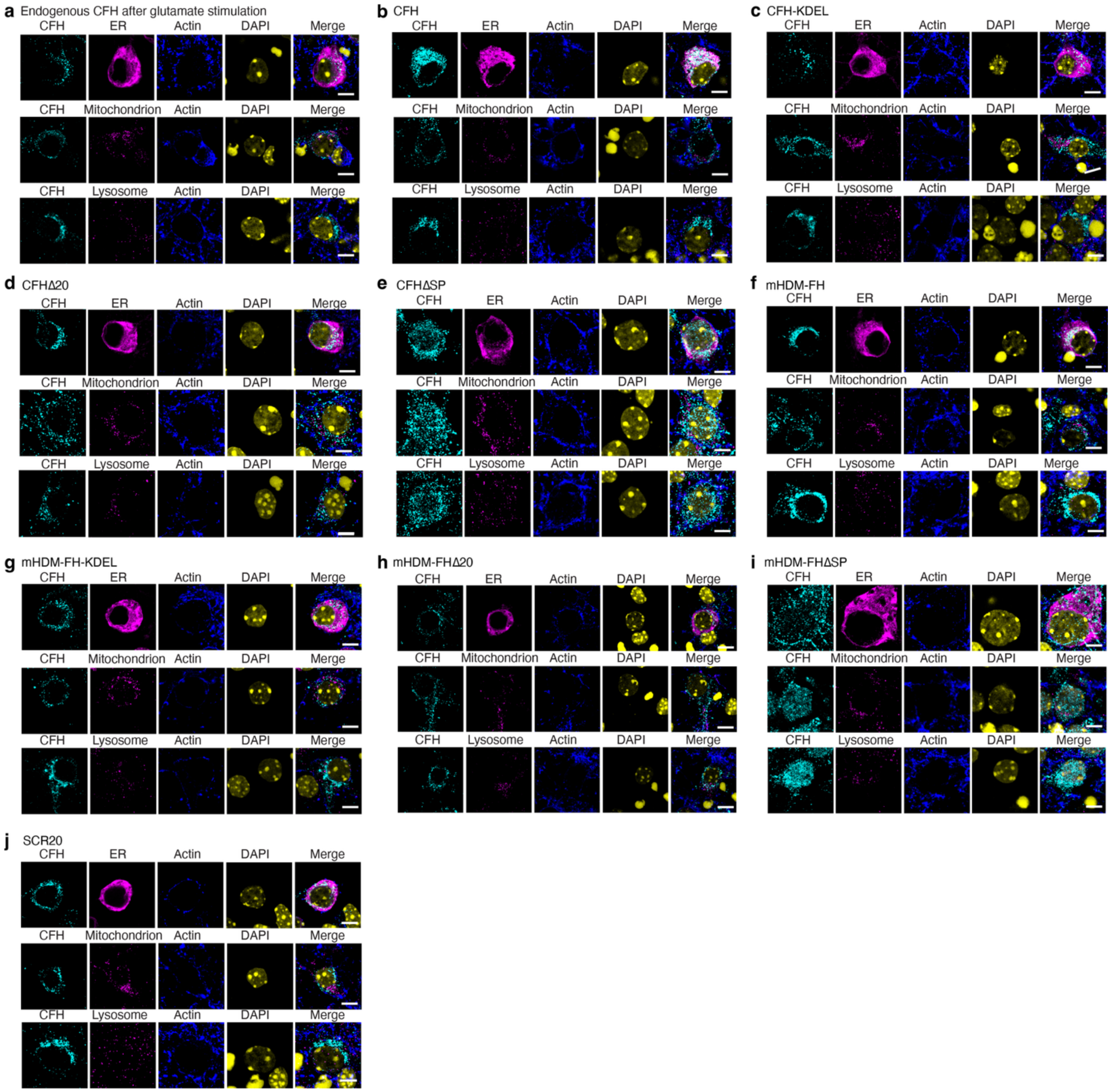

Supplementary Fig.7 | Subcellular localisation of CFH-derived constructs in primary cortical neurons. a–j, Representative images of primary cortical neurons stimulated with glutamate showing endogenous CFH expression (**a**) or expressing CFH (**b**), CFH-KDEL (**c**), CFHΔ20 (**d**), CFHΔSP (**e**), mHDM-FH (**f**), mHDM-FH-KDEL (**g**), mHDM-FHΔ20 (**h**), mHDM-FHΔSP (**i**), or SCR20 (**j**). Neurons were co-transduced with a lentiviral vector expressing GFP-KDEL to visualise the endoplasmic reticulum (ER; top panels), immunostained for TOMM20 to label mitochondria (middle panels), or immunostained for LAMP1 to label lysosomes (bottom panels). Nuclei were counterstained with Hoechst, and the plasma membrane was visualised with phalloidin staining of actin**.** Scale bar, 5 µm.

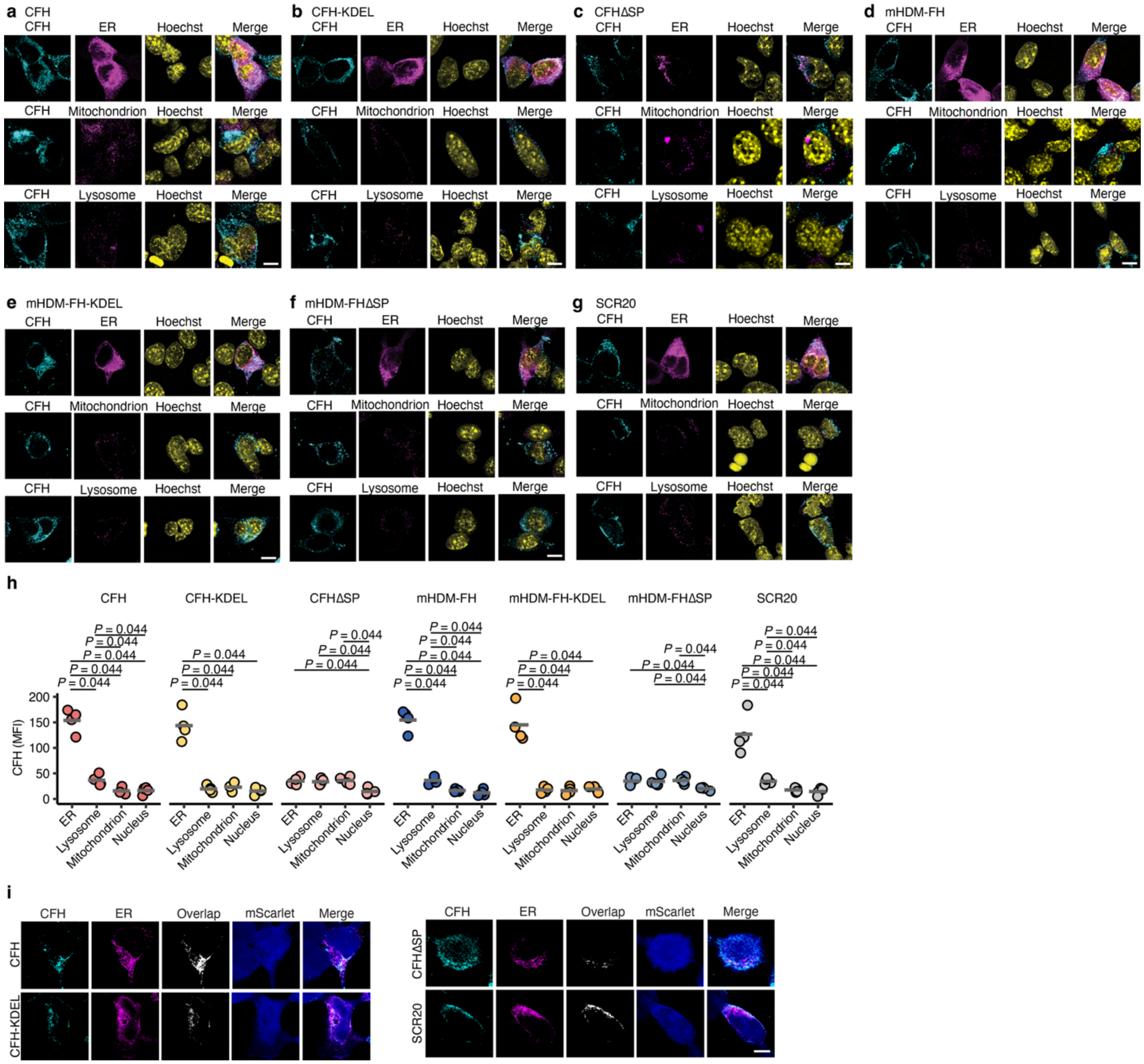

Supplementary Fig.8 | Subcellular localisation of CFH-derived constructs in Pfa1 cells. a–g, Representative images of Pfa1 cells expressing CFH (**a**), CFH-KDEL (**b**), CFHΔSP (**c**), mHDM-FH (**d**), mHDM-FH-KDEL (**e**), mHDM-FHΔSP (**f**), or SCR20 (**g**). Cells were co-transduced with a lentiviral vector expressing GFP-KDEL to visualise the endoplasmic reticulum (ER; top panels), immunostained for TOMM20 to label mitochondria (middle panels), or immunostained for LAMP1 to label lysosomes (bottom panels). Nuclei were counterstained with Hoechst. Scale bar, 5 µm. **h**, Quantification of CFH mean fluorescence intensity (MFI) within the indicated subcellular compartments (n = 4 per construct). **i**, Representative images showing ER localisation of expressed CFH, CFH-KDEL, CFHΔSP, or SCR20. mScarlet was co-expressed to visualise the entire cell. Representative of four independent experiments with similar results; quantification is shown in h. Scale bar, 5 µm. Individual data points represent biological replicates; bars indicate the mean. Statistical comparisons used FDR-corrected unpaired two-sided Mann-Whitney U tests.

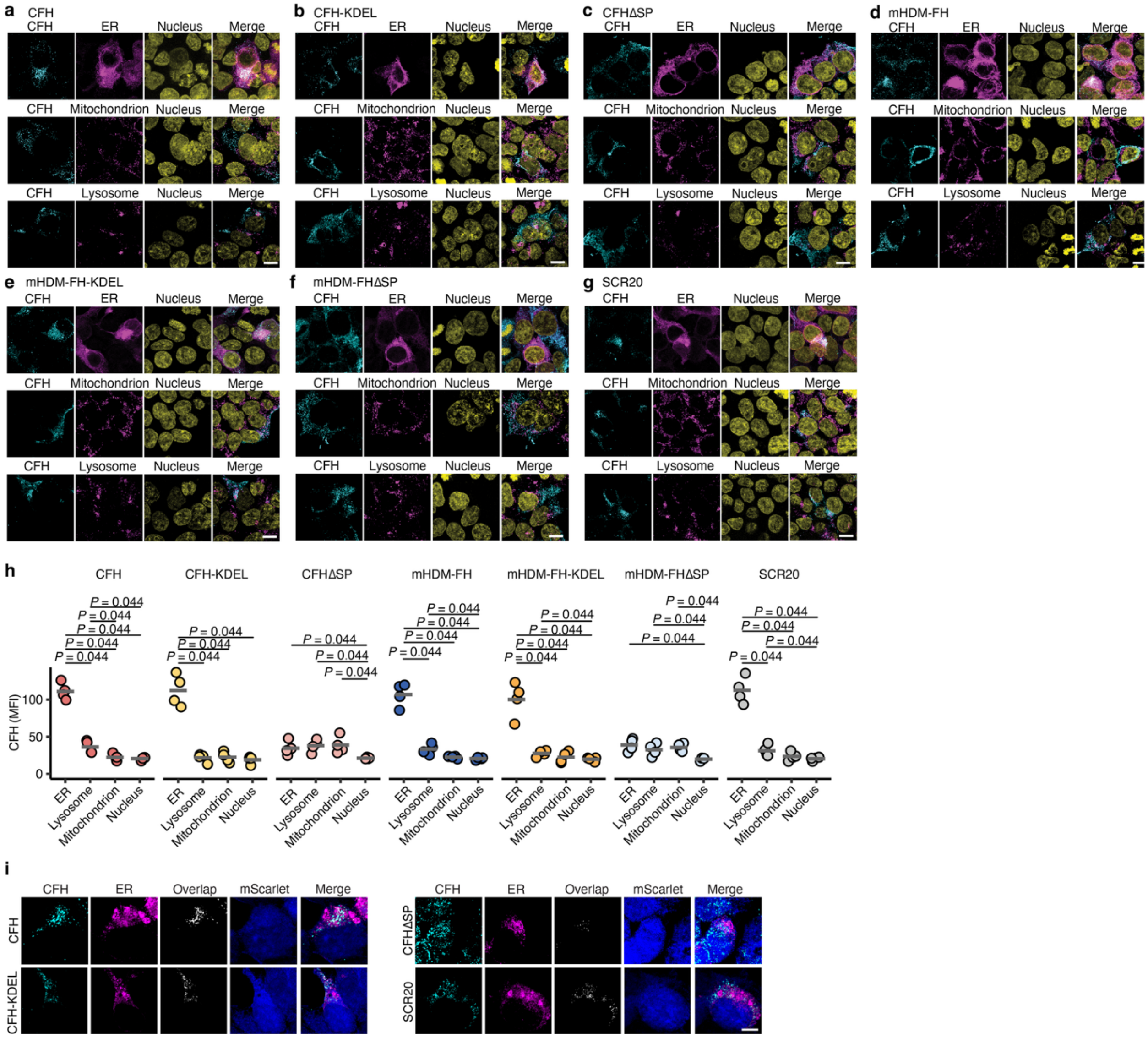

Supplementary Fig.9 I Subcellular localisation of CFH-derived constructs in HEK293T cells. a–g, Representative images of HEK293T cells expressing CFH (**a**), CFH-KDEL (**b**), CFHΔSP (**c**), mHDM-FH **(d)**, mHDM-FH-KDEL (**e**), mHDM-FHΔSP (**f**), or SCR20 (**g**). Cells were co-transduced with a lentiviral vector expressing GFP-KDEL to visualise the endoplasmic reticulum (ER; top panels), immunostained for TOMM20 to label mitochondria (middle panels), or immunostained for LAMP1 to label lysosomes (bottom panels). Nuclei were counterstained with Hoechst. Scale bar, 5 µm. **h**, Quantification of CFH mean fluorescence intensity (MFI) within the indicated subcellular compartments ($n = 4$ per construct). **i**, Representative images showing ER localisation of expressed CFH, CFH-KDEL, CFHΔSP, or SCR20. mScarlet was co-expressed to visualise the entire cell. Scale bar, 5 µm. Representative of four independent experiments with similar results; quantification is shown in h. Individual data points represent biological replicates; bars indicate the mean. Statistical comparisons used FDR-corrected unpaired two-sided Mann-Whitney *U* tests.

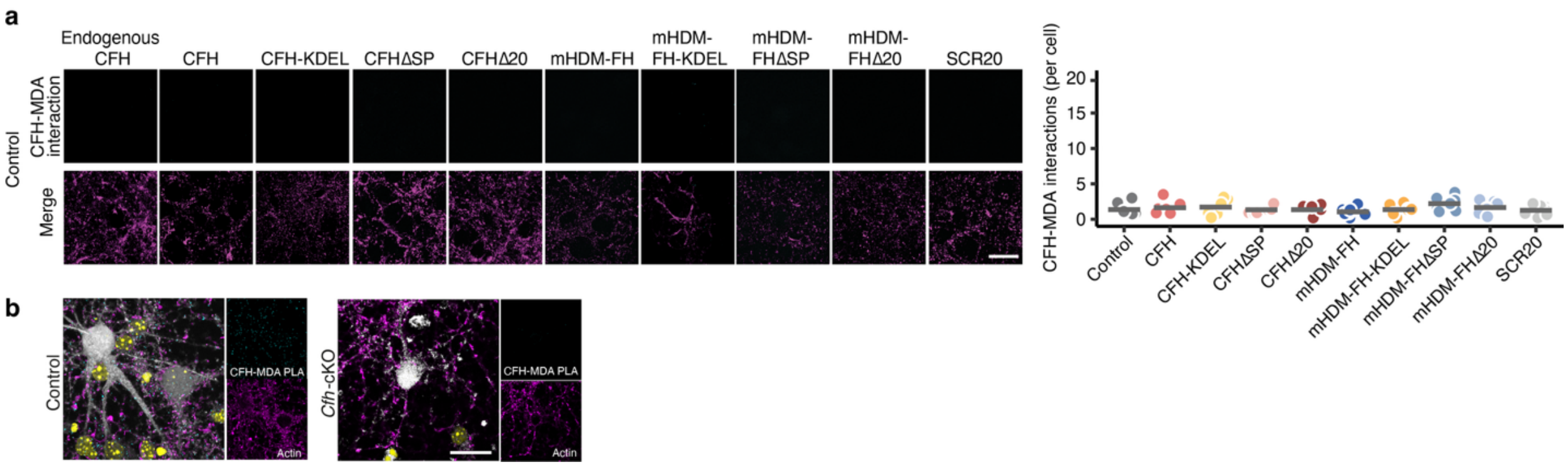

Supplementary Fig.10 | Close proximity of CFH and MDA-modified proteins in neurons. a, Proximity ligation assay (PLA) between CFH and MDA-modified proteins in unstimulated primary cortical neurons. Representative images (left) and quantification of puncta per cell (right) ($n = 7$ biological replicates per construct). Scale bar, 50 µm. **b**, Representative image of PLA between CFH and MDA-modified proteins in control and *Cfh*-cKO primary neuronal cultures after glutamate stimulation. The experiment was performed once. Scale bar, 20 µm. Individual data points represent biological replicates; bars indicate the mean. Statistical comparisons used FDR-corrected unpaired two-tailed Mann-Whitney *U* tests against controls.

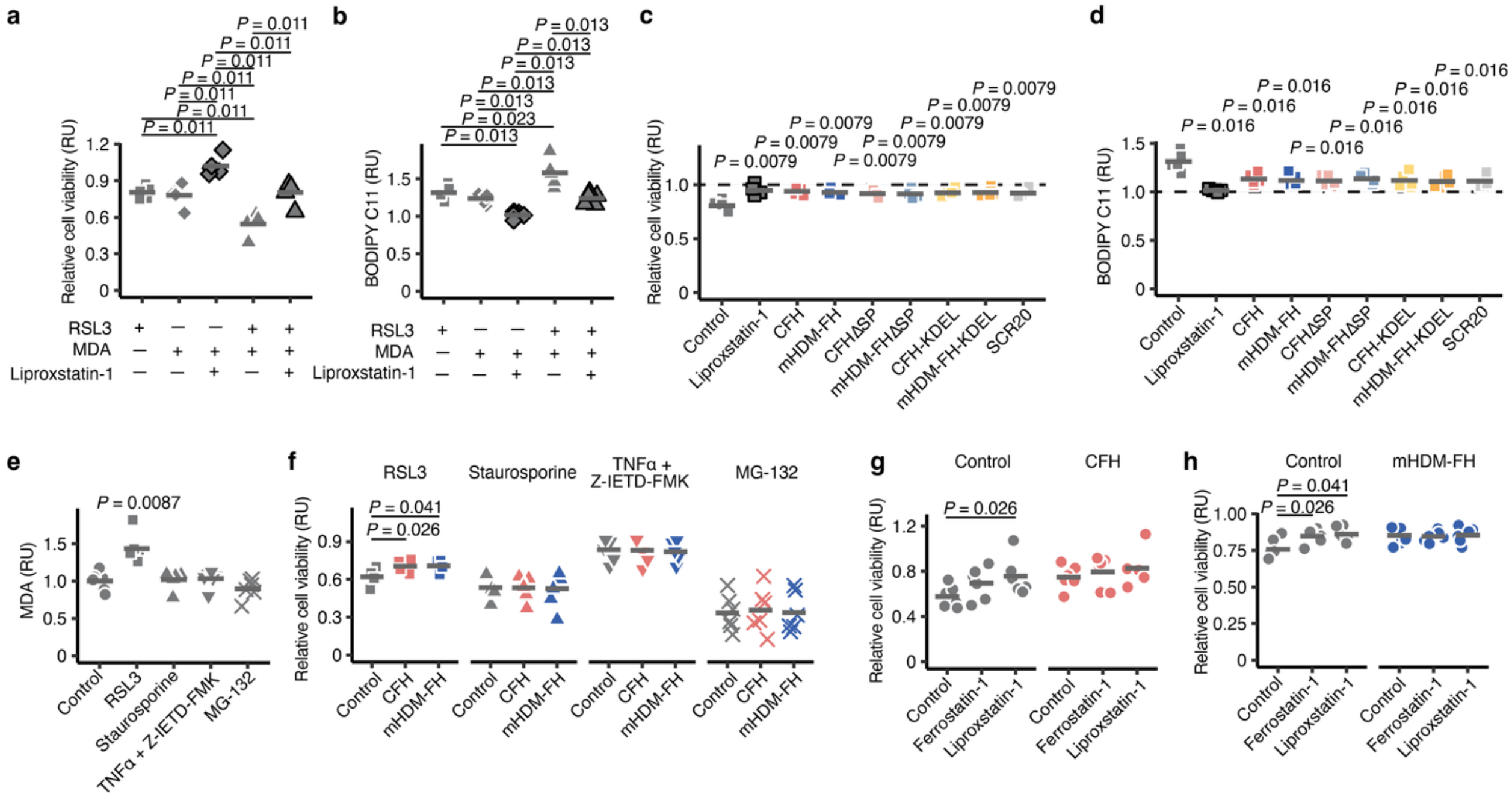

Supplementary Fig.11 | CFH-mediated protection against ferroptosis extends beyond neurons. a,**b**, Relative cell viability (**a**) and lipid peroxidation measured by BODIPY C11 mean fluorescence intensity (MFI) (**b**) in Pfa1 cells following stimulation with RSL3, MDA, or both, with or without liproxstatin-1 pretreatment (*n* = 5 per condition). **c**,**d**, Relative cell viability (**c**), and lipid peroxidation (**d**) in Pfa1 cells following treatment with RSL3 in mScarlet-expressing controls, liproxstatin-1 pretreated cells, or in cells transduced to express CFH, mHDM-FH, CFHΔSP, mHDM-FHΔSP, CFH-KDEL, mHDM-FH-KDEL, or SCR20 (*n* = 5 per construct and condition). **e**,**f**, MDA-modified protein levels relative to unstimulated controls (**e**) and relative cell viability of primary neurons expressing mScarlet, CFH, or mHDM-FH (**f**) following stimulation with RSL3, staurosporine, TNFα plus Z-IETD-FMK, or MG-132 (*n* = 6 per condition). **g**,**h**, Relative cell viability of CFH- (**g**) or mHDM-FH-expressing (**h**) primary neurons compared with mScarlet controls following glutamate treatment, with or without pretreatment with ferrostatin-1 or liproxstatin-1 (*n* = 6 per condition). Individual data points represent biological replicates; bars indicate the mean. Control groups are shared between **a** and **c**, and between **b** and **d**. Statistical comparisons used FDR-corrected two-sided Mann-Whitney *U* tests (**a–b**) against controls (**c–e**), or two-sided Mann–Whitney *U* tests (**f–h**). RU, relative units.

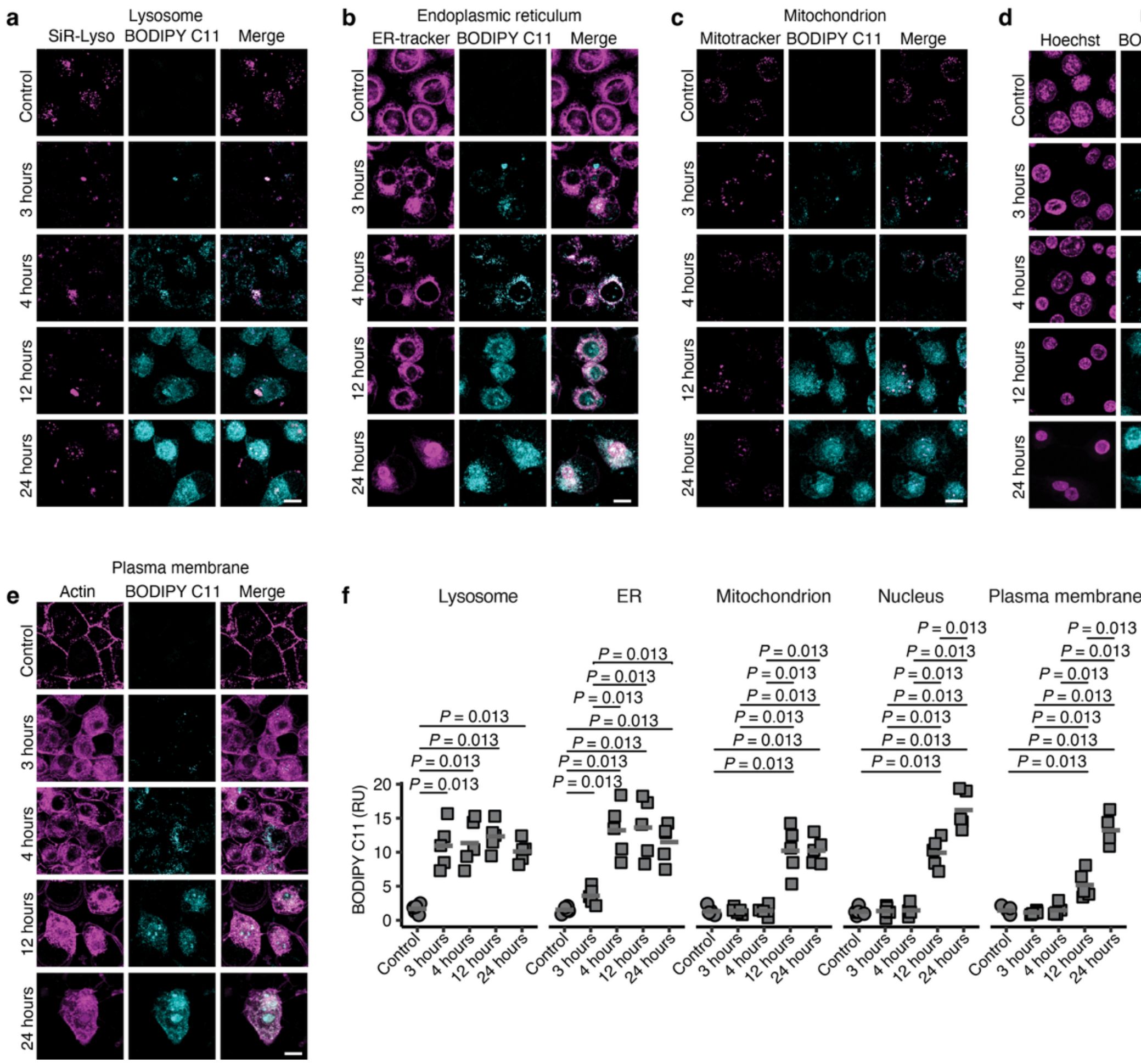

Supplementary Fig.12 | Early lysosomal lipid peroxidation in Pfa1 cells. a–e, Representative images of unstimulated Pfa1 cells and cells 3, 4, 12, and 24 h after treatment with RSL3. Lipid peroxidation was visualised using the BODIPY C11. Live-cell organelle markers were used to identify lysosomes (SiR-Lyso; **a**), endoplasmic reticulum (ER-Tracker; **b**), mitochondria (MitoTracker; **c**), nuclei (Hoechst; **d**), and the plasma membrane (phalloidin–actin staining; **e**). Scale bar, 5 µm. **f,** Quantification of colocalisation between BODIPY C11 and the corresponding organelle markers shown in **a–e** (*n* = 5 biological replicates per time point; RU, relative units). Individual data points represent biological replicates; bars indicate the mean. Statistical comparisons used FDR-corrected two-sided unpaired Mann-Whitney *U* tests.

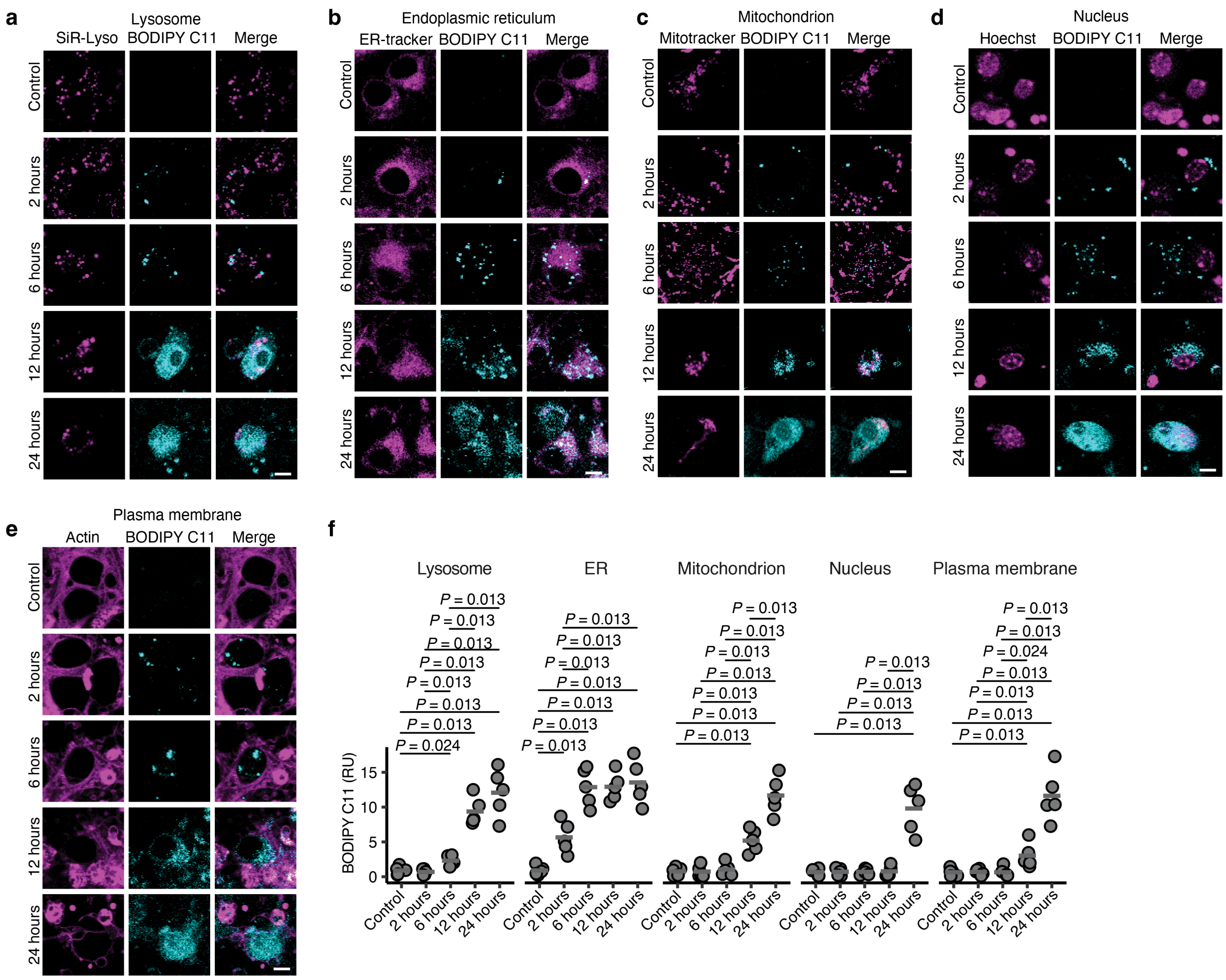

Supplementary Fig.13 | Early endoplasmic reticulum lipid peroxidation in neurons. a–e, Representative images of unstimulated primary cortical neurons and neurons 2, 6, 12, and 24 h after treatment with RSL3. Lipid peroxidation was visualised using the BODIPY C11. Live-cell organelle markers were used to identify lysosomes (SiR-Lyso; **a**), endoplasmic reticulum (ER-Tracker; **b**), mitochondria (MitoTracker; **c**), nuclei (Hoechst; **d**), and the plasma membrane (phalloidin–actin staining; **e**). Scale bar, 5 µm. **f,** Quantification of colocalisation between BODIPY C11 and the corresponding organelle markers shown in **a–e** ($n = 5$ biological replicates per time point; RU, relative units). Individual data points represent biological replicates; bars indicate the mean. Statistical comparisons used FDR-corrected two-sided unpaired Mann-Whitney U tests.

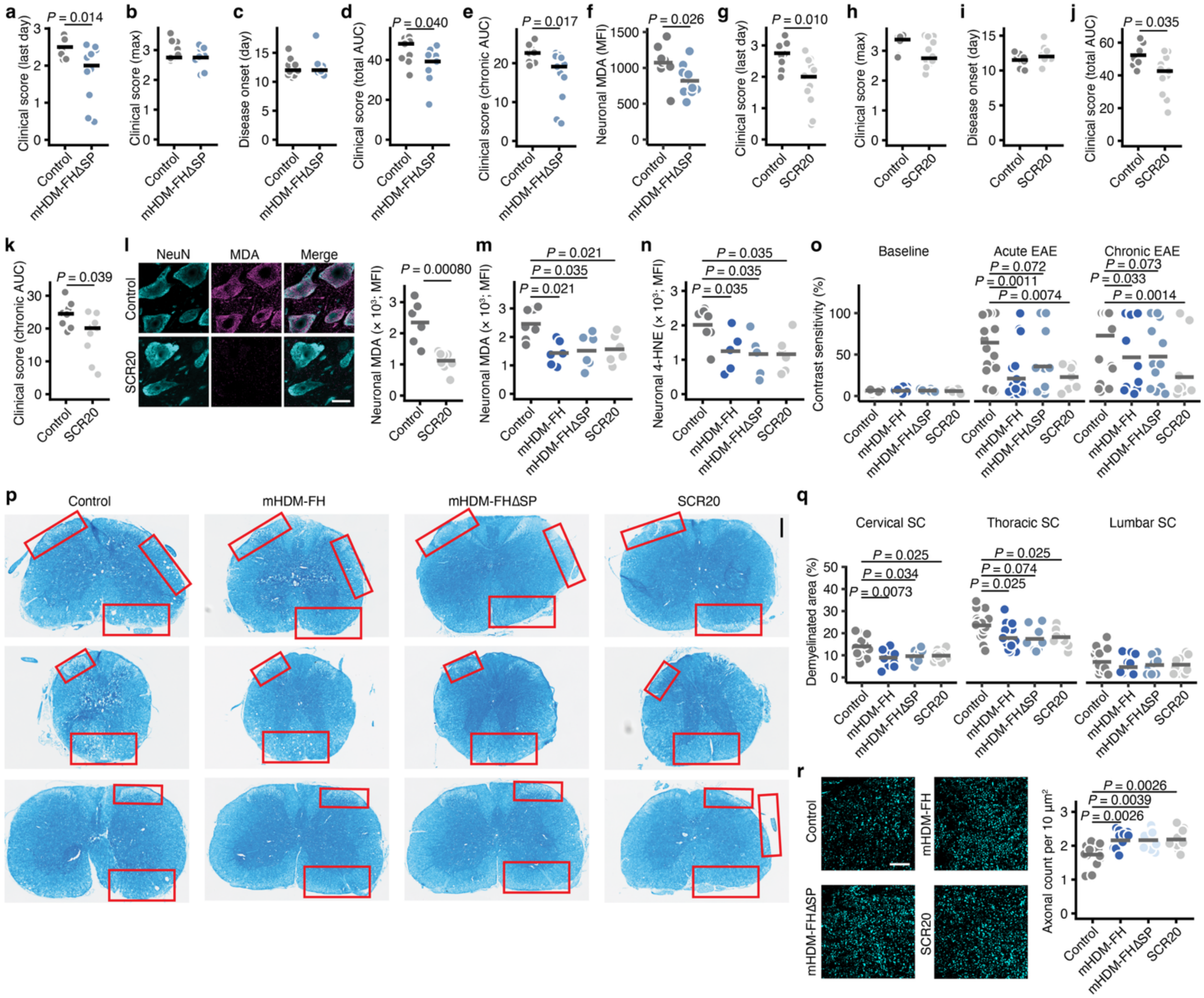

Supplementary Fig.14 | Neuronal mHDM-FHΔSP and SCR20 expression ameliorates clinical and pathological EAE. a–e, Clinical EAE parameters from Fig. 4l in mice expressing neuronal GFP (control, $n = 9$) or mHDM-FHΔSP ($n = 11$): final clinical score (**a**), maximum clinical score (**b**), disease onset (**c**), total area under the curve (AUC; days 0–30 post-immunisation (p.i.)) (**d**), and chronic AUC (days 20–30 p.i.) (**e**). **f**, MFI of MDA-modified proteins in ventral horn (VH) spinal cord neurons at day 30 p.i. (GFP control, $n = 8$; mHDM-FHΔSP, $n = 11$). **g–k**, Clinical EAE parameters from Fig. 4n in mice expressing neuronal GFP (control, $n = 8$) or SCR20 ($n = 11$): final clinical score (**g**), maximum clinical score (**h**), disease onset (**i**), total AUC (**j**), and chronic AUC (days 20–30 p.i.) (**k**). **l**, Representative image (left) and quantification (right) of MDA-modified protein MFI in the VH spinal cord neurons of mice expressing neuronal GFP (control, $n = 6$) or SCR20 ($n = 9$) at day 30 p.i. **m**,**n**, Neuronal MDA-modified protein (**m**), and 4-hydroxynonenal (4-HNE) (**n**) MFI in VH at day 15 p.i. following neuronal expression of GFP (control, $n = 7$), mHDM-FH ($n = 6$), mHDM-FHΔSP ($n = 6$), or SCR20 ($n = 6$). **o**, Visual contrast sensitivity at baseline, acute EAE and chronic EAE in mice expressing GFP (control, $n = 21$ per timepoint), mHDM-FH ($n = 21$ per timepoint), mHDM-FHΔSP ($n = 12$ per timepoint), or SCR20 ($n = 12$ per timepoint), **p**,**q**, Representative Luxol fast blue staining (**p**) and quantification of demyelinated spinal cord white matter (**q**) at day 30 p.i. in mice expressing neuronal GFP (control, $n = 17$), mHDM-FH ($n = 15$), mHDM-FHΔSP ($n = 8$), or SCR20 ($n = 10$). Scale bar, 500 µm. **r**, Representative image (left) and quantification (right) of dorsal column axon numbers following neurofilament staining of mice expressing neuronal GFP (control, $n = 16$), mHDM-FH ($n = 13$), mHDM-FHΔSP ($n = 9$), or SCR20 ($n = 9$). Scale bar, 50 µm. Individual data points represent biological replicates; bars indicate the mean or median (**a–e, g–k**). Statistical comparisons used FDR-corrected two-sided unpaired Mann-Whitney U tests against controls. MFI, Mean fluorescence intensity.

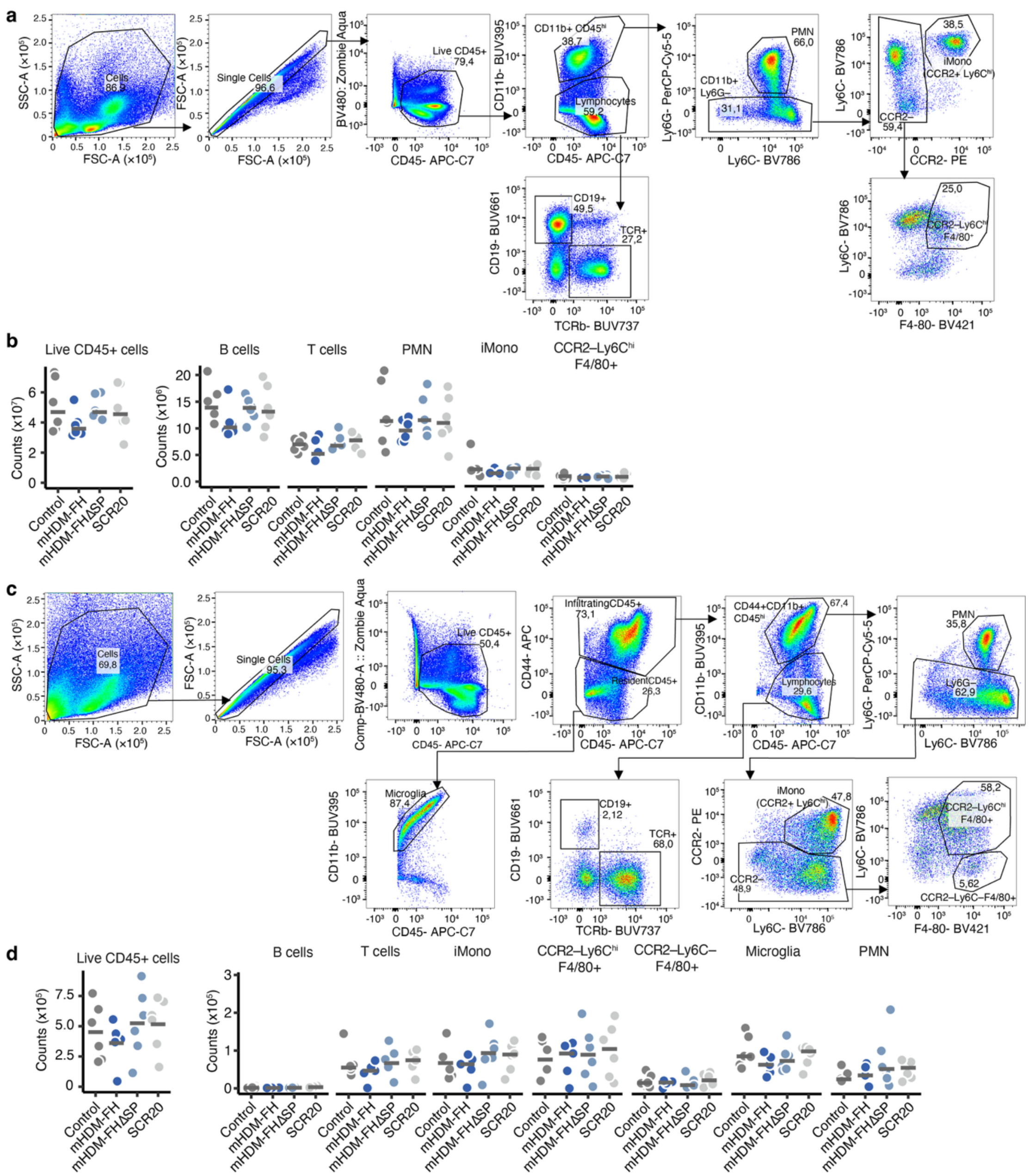

Supplementary Fig.15 | Immune cell infiltration in acute EAE following neuronal CFH-derived construct expression. a,**b**, Representative flow cytometric gating strategy (**a**) and absolute counts (**b**) in the spleen of EAE mice at day 15 post-immunisation (p.i.) following neuronal expression of GFP (control), mHDM-FH, mHDM-FHΔSP, or SCR20 (*n* = 6 per construct). Live CD45⁺ leukocytes were separated into CD11b⁺ myeloid cells and lymphocytes. Myeloid cells were further subdivided into CD11b⁺Ly6G⁺ neutrophils (PMN), Ly6C⁺CCR2⁺ inflammatory monocytes, and CCR2⁻F4/80⁺Ly6C⁺ macrophages, whereas lymphocytes were classified as CD19⁺ B cells and TCRβ⁺ T cells. **c**,**d**, Representative gating strategy (**c**) and absolute immune cell counts (**d**) in the spinal cord of EAE mice at day 15 p.i. following neuronal expression of GFP (control, *n* = 6), mHDM-FH (*n* = 5), mHDM-FHΔSP (*n* = 6) and SCR20 (*n* = 6). The same mice were analysed as in **a**,**b**. Live CD45⁺ leukocytes were separated into CD44+ infiltrating leukocytes and CD44⁻ resident cells, followed by subdivision into CD11b⁺ myeloid cells and CD11b⁻ lymphocytes. Myeloid cells were further resolved into CD11b⁺Ly6G⁺ neutrophils (PMN) and CD11b⁺Ly6G⁻ monocyte-derived cells, the latter comprising Ly6C⁺CCR2⁺ inflammatory monocytes and CCR2⁻F4/80⁺Ly6C⁺ macrophages. Lymphocyte were classified as CD19⁺ B cells and TCRβ⁺ T cells, and CD11b⁺CD44⁻ cells were identified as microglia/resident macrophages. Individual data points represent biological replicates; bars indicate the mean. Statistical comparisons used FDR-corrected two-sided unpaired Mann-Whitney *U* tests.

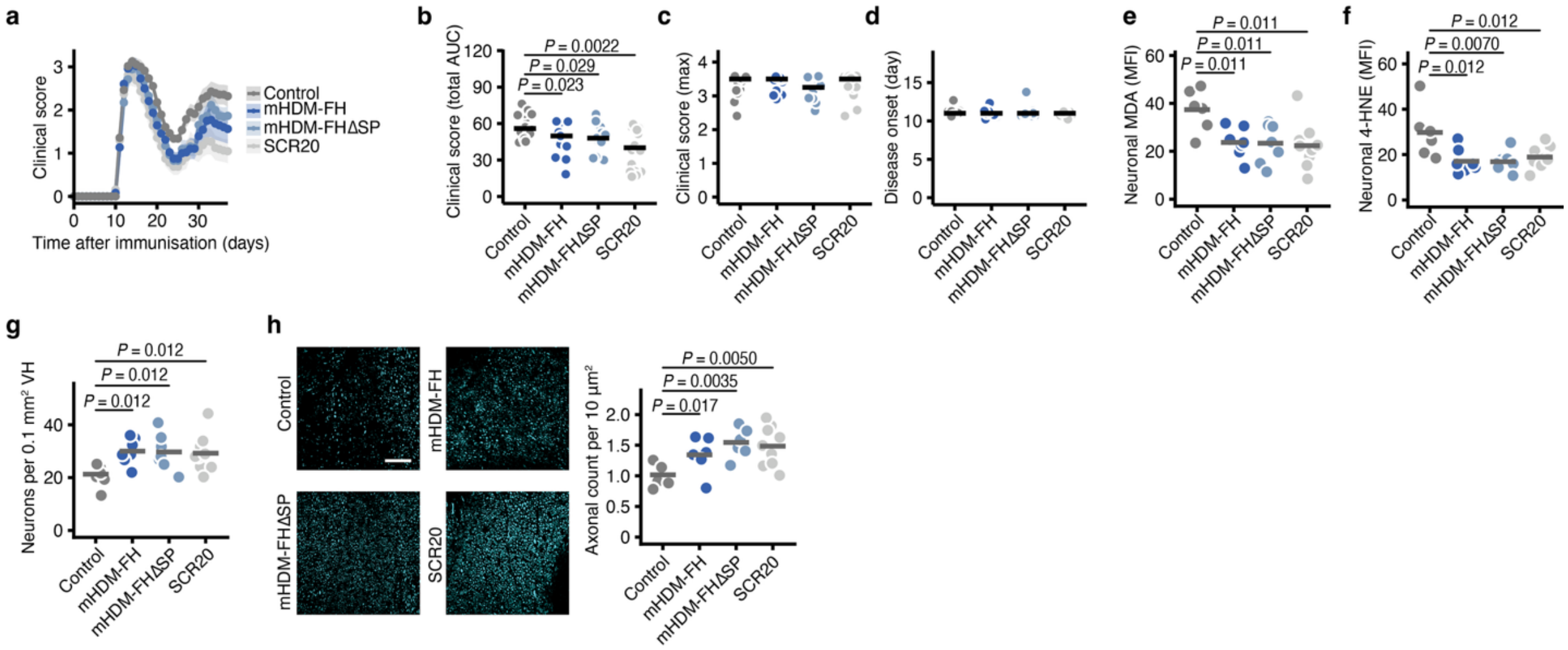

Supplementary Fig.16 | Neuronal CFH constructs are protective in relapsing-remitting EAE in SJL mice. a, EAE clinical disease score (arbitrary units, AU) in SJL mice expressing neuronal GFP (control, $n = 14$), mHDM-FH ($n = 12$), mHDM-FHΔSP ($n = 13$), or SCR20 ($n = 16$). Data are shown as mean ± s.e.m.; symbols indicate the mean and shaded areas the s.e.m. **b–d**, Clinical EAE parameters. total area under the curve (AUC, 0–37 days post-immunisation (p.i.)) (**b**), maximum clinical score (**c**), and disease onset (**d**). **e**,**f**, Mean fluorescence intensity (MFI) of MDA-modified proteins (**e**) and 4-hydroxynonenal (4-HNE) (**f**) in ventral horn (VH) spinal cord neurons at EAE day 37 p.i. (GFP control, $n = 7$; mHDM-FH, $n = 7$; mHDM-FHΔSP, $n = 7$; SCR20, $n = 9$). **g**, VH spinal cord neuronal counts at day 37 p.i. (GFP control, $n = 7$; mHDM-FH, $n = 7$; mHDM-FHΔSP, $n = 7$; or SCR20 $n = 9$). **h**, Representative image (left) and quantification (right) of dorsal column axon numbers at day 37 p.i. (GFP control, $n = 7$; mHDM-FH, $n = 7$; mHDM-FHΔSP, $n = 7$; or SCR20, $n = 9$). Scale bar, 50 µm. Individual data points represent biological replicates; bars indicate the mean or median (**b–d**). Statistical comparisons used unpaired two-sided Mann-Whitney U tests (**a–d**), or FDR-corrected two-sided unpaired Mann-Whitney *U* tests against controls (**e–h**).

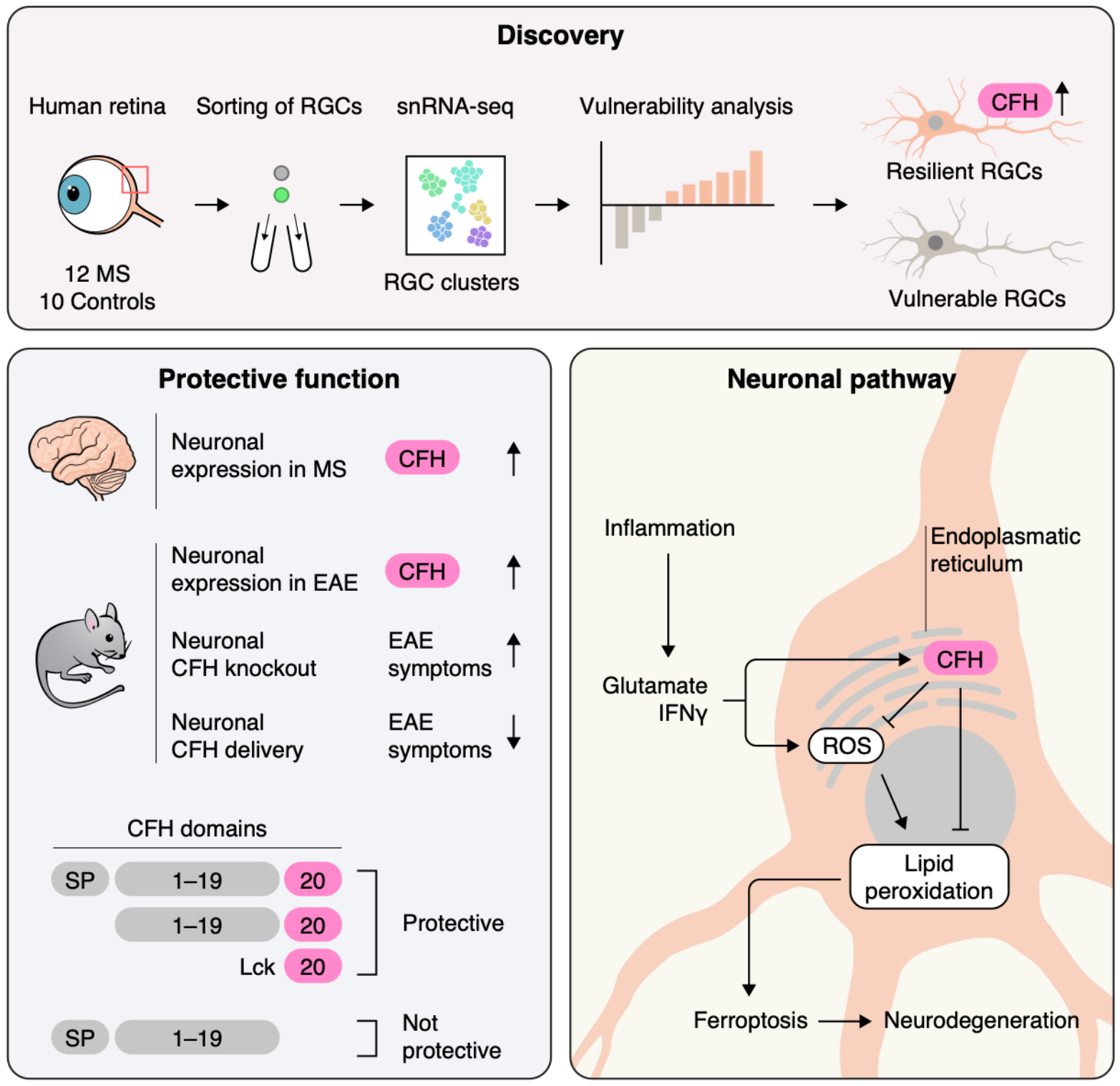

Supplementary Fig.17 | Graphical summary. Single-nucleus RNA sequencing (snRNA-Seq) was performed on human postmortem retinal ganglion cell (RGC) nuclei from multiple sclerosis (MS) and control donors, followed by bioinformatic analyses to identify the molecular determinants of neuronal susceptibility and resilience (e.g., complement factor H; CFH) to inflammation-induced degeneration (**Discovery**). CFH is induced not only in RGCs but also in cortical and spinal cord neurons during MS and experimental autoimmune encephalomyelitis (EAE). Phenotypic assessments of neuron-specific CFH knockout and the overexpression of various truncated CFH constructs revealed that the C-terminal domain of CFH confers neuroprotection (**Protective function**). Mechanistic studies in primary cortical neurons demonstrated that stress-induced intracellular CFH (e.g., by glutamate, IFNγ) protects against oxidative cell damage and ferroptosis by limiting reactive oxygen species (ROS) and lipid peroxidation independent of its secretion by localising to the endoplasmic reticulum (**Neuronal pathway**).

Supplementary Fig.18 | The figures shown below present the Source Data Figures of original uncropped immunoblot images. a, Related to Extended Gata Fig. 4b and 5m. **b**, Related to Extended Gata Fig. 5n. **c,** Related to Extended Gata Fig. 7e. **d**, Related to Extended Gata Fig. 7g. **e**, Related to Extended Gata Fig. 7h.

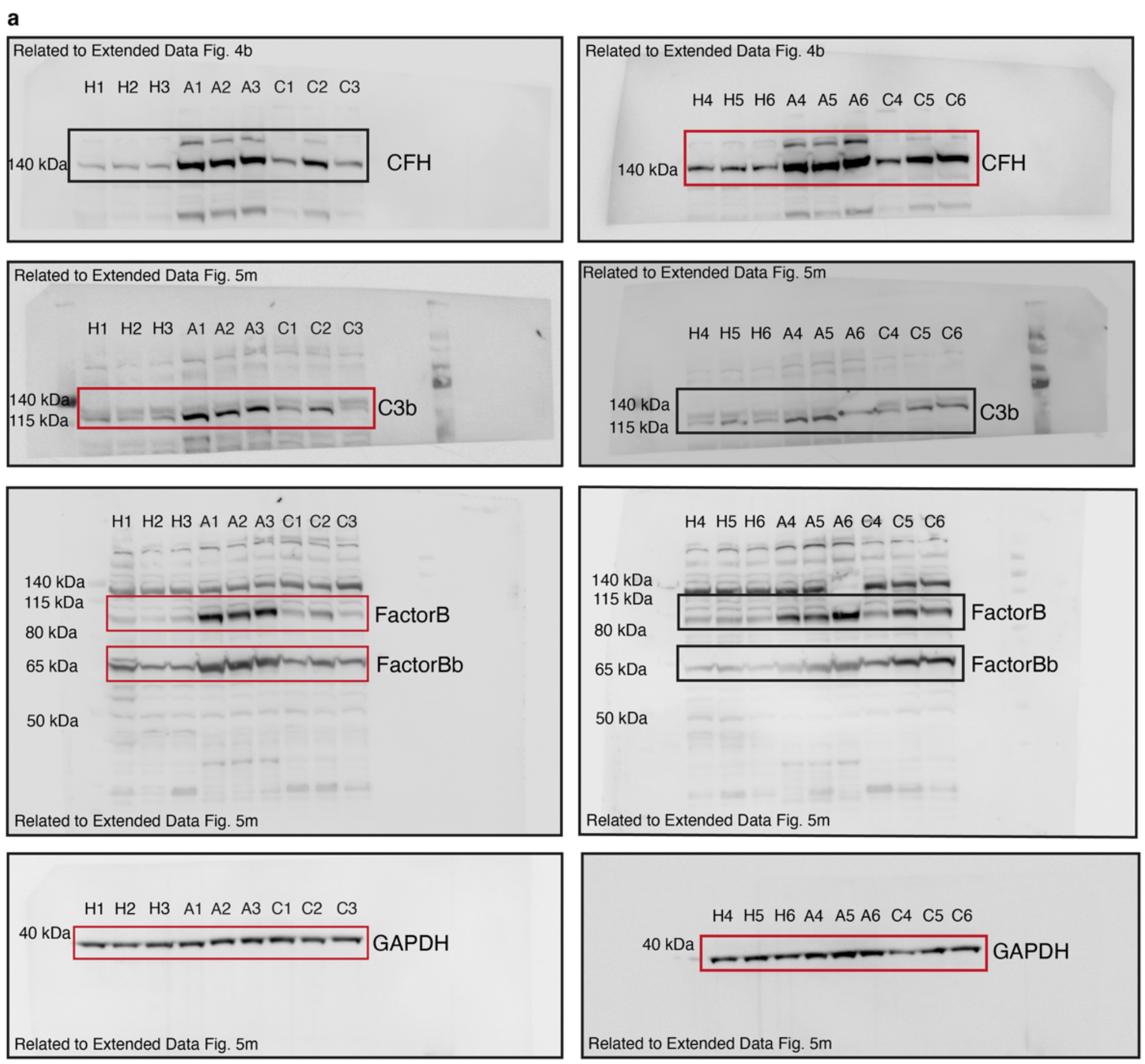

b Related to Extended Data Fig. 5n

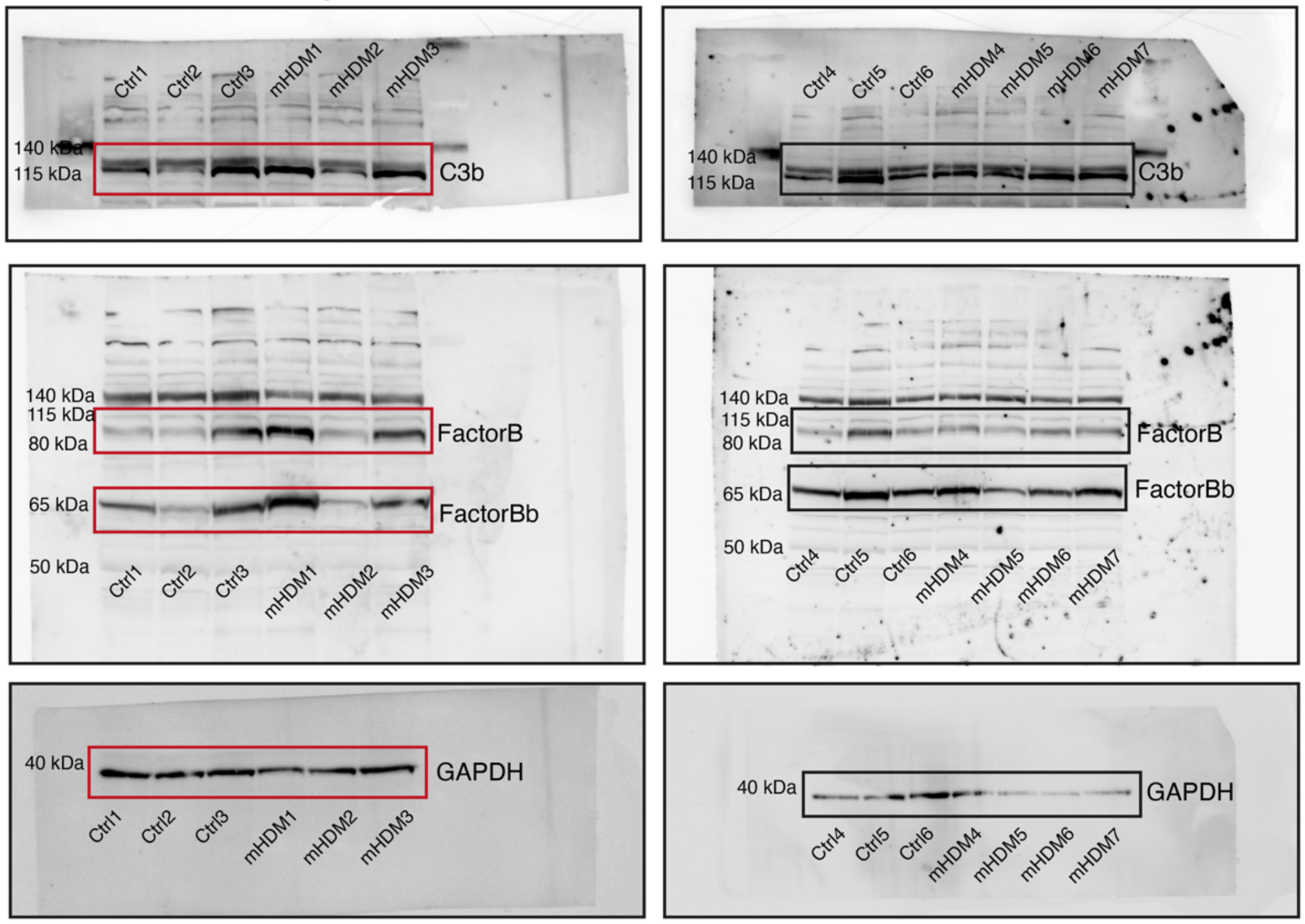

c Related to Extended Data Fig. 7e

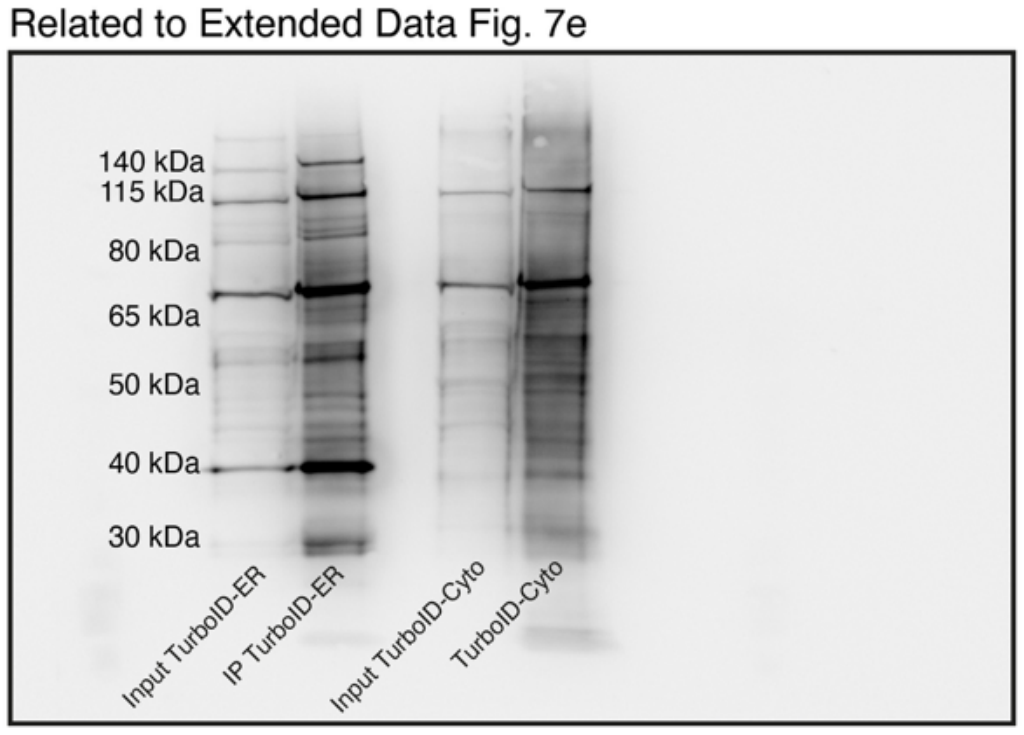

d Related to Extended Data Fig. 7g

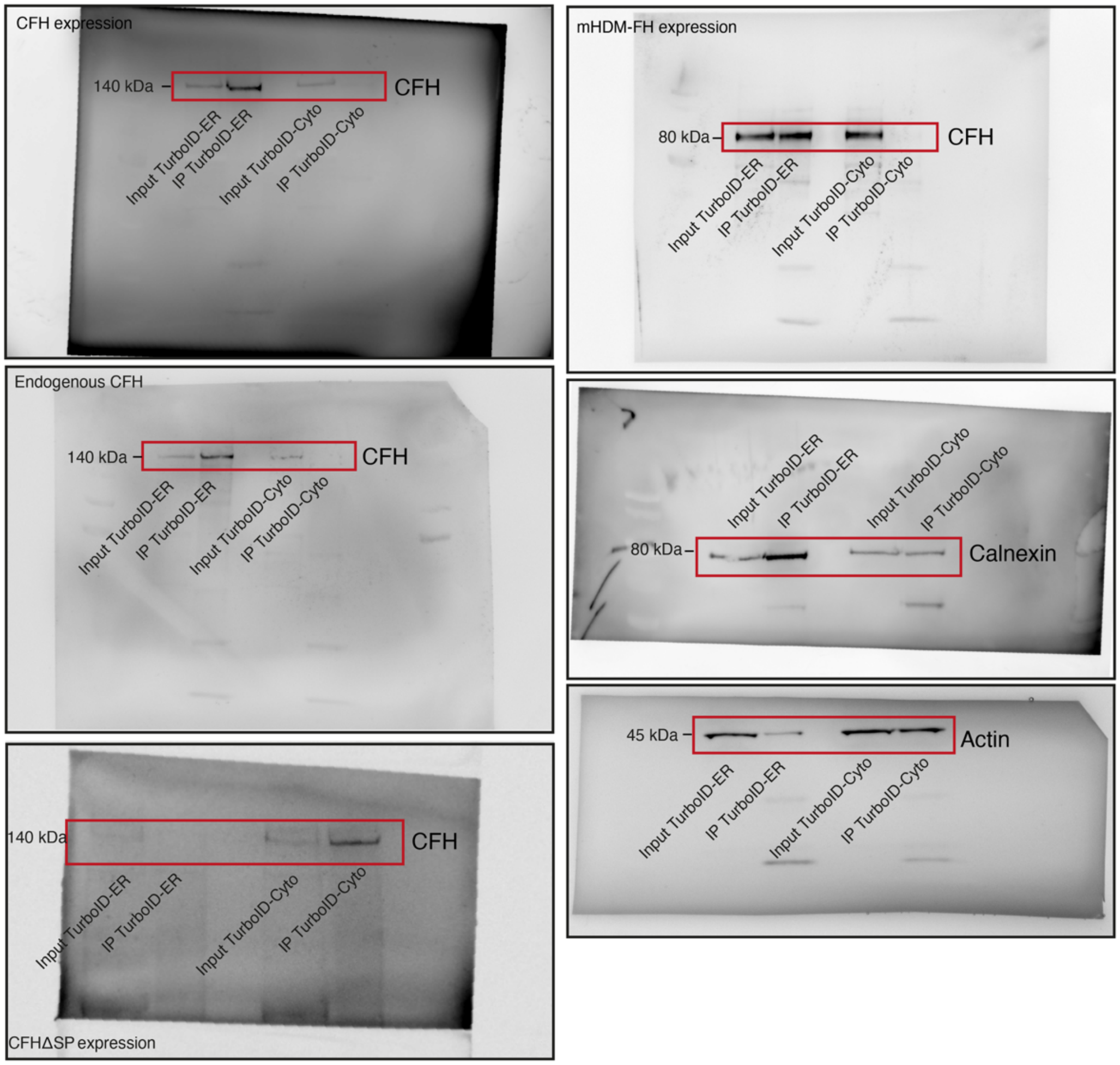

e Related to Extended Data Fig. 7h

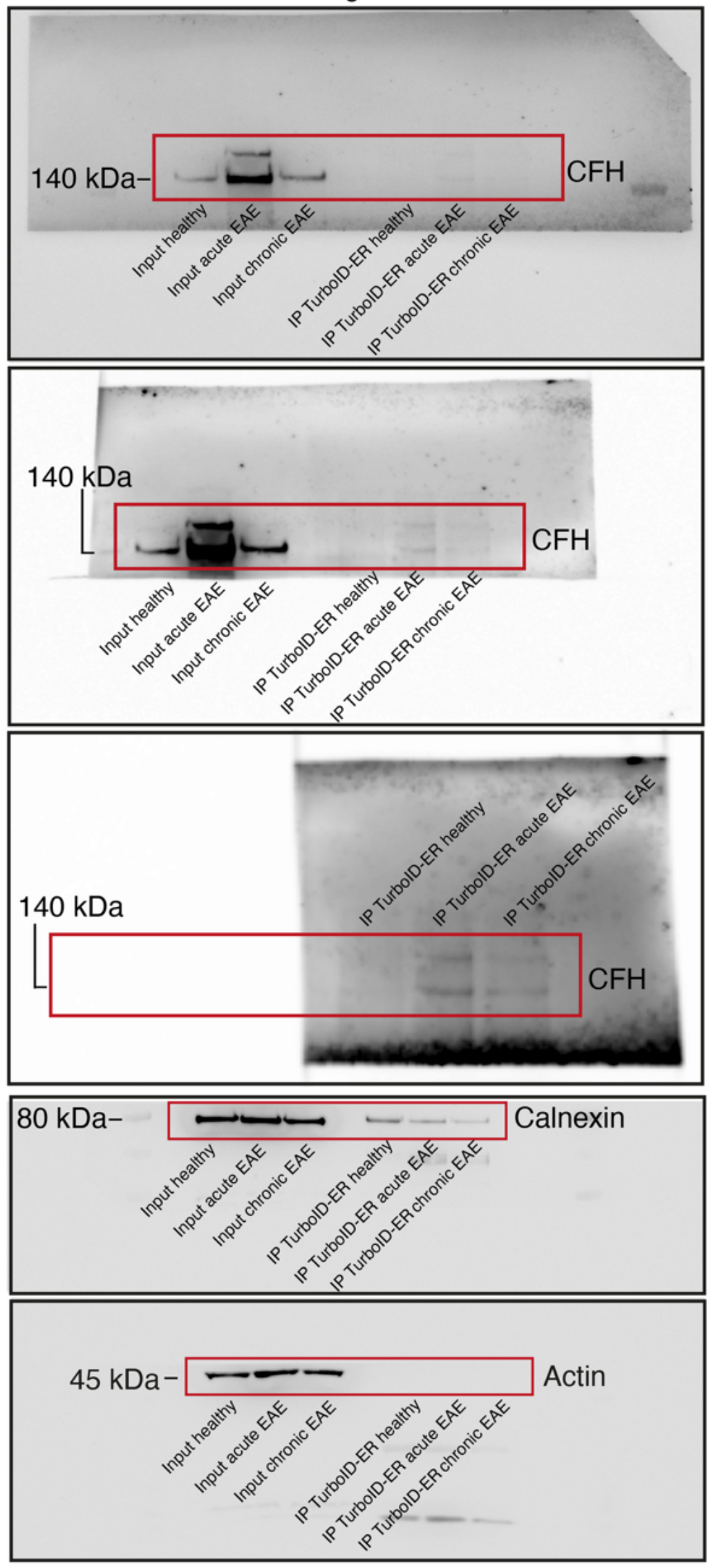

Supplement: Supplementary file 1 [file 41586_2026_10981_MOESM1_ESM.pdf]
