# Supplementary material for: Intracellular complement factor H protects neurons during CNS inflammation
Source: Nature. 2026 Sep 2;658(8136):765–73. doi: 10.1038/s41586-026-10981-y (PMC13668678; doi:10.1038/s41586-026-10981-y)
Supplement: Supplementary file 2 [file 41586_2026_10981_MOESM2_ESM.pdf]

nature portfolio

Corresponding author(s): Manuel Friese

Last updated by author(s): Jul 28, 2026

Reporting Summary

Nature Portfolio wishes to improve the reproducibility of the work that we publish. This form provides structure for consistency and transparency in reporting. For further information on Nature Portfolio policies, see our Editorial Policies and the Editorial Policy Checklist.

Statistics

For all statistical analyses, confirm that the following items are present in the figure legend, table legend, main text, or Methods section.

n/a	Confirmed	
☐	☒	The exact sample size (n) for each experimental group/condition, given as a discrete number and unit of measurement
☐	☒	A statement on whether measurements were taken from distinct samples or whether the same sample was measured repeatedly
☐	☒	The statistical test(s) used AND whether they are one- or two-sided<br>*Only common tests should be described solely by name; describe more complex techniques in the Methods section.*
☒	☐	A description of all covariates tested
☐	☒	A description of any assumptions or corrections, such as tests of normality and adjustment for multiple comparisons
☐	☒	A full description of the statistical parameters including central tendency (e.g. means) or other basic estimates (e.g. regression coefficient) AND variation (e.g. standard deviation) or associated estimates of uncertainty (e.g. confidence intervals)
☐	☒	For null hypothesis testing, the test statistic (e.g. F, t, r) with confidence intervals, effect sizes, degrees of freedom and P value noted<br>*Give P values as exact values whenever suitable.*
☒	☐	For Bayesian analysis, information on the choice of priors and Markov chain Monte Carlo settings
☒	☐	For hierarchical and complex designs, identification of the appropriate level for tests and full reporting of outcomes
☒	☐	Estimates of effect sizes (e.g. Cohen's d, Pearson's r), indicating how they were calculated

Our web collection on statistics for biologists contains articles on many of the points above.

Software and code

Policy information about availability of computer code

Data collection	For flow cytometric nuclei sorting of retinal ganglion cell nuclei, BD FACS Aria III running BD FACSDiva v9.0.1 software (BD Biosciences) was used. Single-nucleus RNA-sequencing data was collected using the 10X Genomics Chromium Single Cell 3'v3 kit. cDNA libraries were measured with the Agilent Bioanalyzer (TapeStation 4150). Sequencing was performed on a Illumina NovaSeq6000 platform.<br>Flow cytometry data collection was performed on a BD Symphony A3 with FACSDiva software version 9.1 (BD Biosciences).<br>Confocal images were acquired on a Zeiss LSM 900 Airyscan 2 confocal microscope equipped with ZEN blue software v3.9. SmFISH images were acquired with a Leica DM6 B microscope equipped with a Leica K5 camera and the THUNDER imaging system for preprocessing.<br>Immunoblots were acquired with LAS-4000 mini v2.11 Image-Reader software. Slides stained with Luxol fast blue were imaged with a NanoZoomer 2.0-RS digital slide scanner with NDP.view2 software. RT-qPCR data were acquired using QuantStudio Flex 6 Real-Time PCR System.
Data analysis	SmFISH images were analysed with QuPath (v0.4.3).<br>Flow cytometry analysis was performed with FlowJo v10.9 (BD Biosciences).<br>Immunoblot, fluorescence microscopy, and bright-field microscopy images were analysed with Fiji (ImageJ, v2.14.0).<br>Bioinformatic analyses were performed using the following software packages: Cell Ranger v7.0.1, CellBender v0.3.0, R v4.4.1, tidyverse v2.0.0, Seurat v5.1.0, clusterProfiler v4.12.6, ggplot2 v3.5.1, ggplotify v0.1.2, openxlsx v4.2.7.1, reticulate v1.39.0, tidyheatmaps v0.2.1, DOSE v3.30.5, msigdbr v7.5.1, Broom v1.0.7, outliers v0.15, scCustomize v2.1.2, scDblFinder v1.18.0, BiocParallel v1.38.0, ggpubr v0.6.0, biomaRt v2.60.1, dendextend v1.18.0, viridis v0.6.5, org.Hs.eg.db v3.19.1, DESeq2 v1.44, ggrepel v0.9.6, enrichplot v1.24.4, EnvStats v3.0.0, rstatix v0.7.2, geneName v0.2.3, AUCell v1.26.

For manuscripts utilizing custom algorithms or software that are central to the research but not yet described in published literature, software must be made available to editors and reviewers. We strongly encourage code deposition in a community repository (e.g. GitHub). See the Nature Portfolio guidelines for submitting code & software for further information.

Data

Policy information about availability of data

All manuscripts must include a data availability statement. This statement should provide the following information, where applicable:

- Accession codes, unique identifiers, or web links for publicly available datasets
- A description of any restrictions on data availability
- For clinical datasets or third party data, please ensure that the statement adheres to our policy

Raw human retina snRNA-sequencing data have been deposited in the ENA (European Nucleotide Archive) database under the accession number PRJEB102624. Processed snRNA-seq data, including the raw count matrix after quality control and corresponding cell- and sample- level metadata, are available via Figshare (DOI: 10.6084/m9.figshare.33075182). Sequencing reads were aligned using the 10x Genomics Cell Ranger GRCh38 reference (2020-A) (https://www.10xgenomics.com/support/software/cell-ranger/downloads/cr-ref-build-steps#human-ref-2020-a). Raw bulk mRNA-sequencing data are available from GEO under the accession number GSE332759. Mouse sequencing reads were aligned using the 10x Genomics Cell Ranger mm10 reference (2020-A). (https://www.10xgenomics.com/support/software/cell-ranger/downloads/cr-ref-build-steps#mouse-ref-2020-a). The mass spectrometry proteomics data have been deposited to the ProteomeXchange Consortium via the PRIDE [PubMed ID: 39494541] partner repository with the data set identifier PXD080433.

Research involving human participants, their data, or biological material

Policy information about studies with human participants or human data. See also policy information about sex, gender (identity/presentation), and sexual orientation and race, ethnicity and racism.

Reporting on sex and gender	We report the sex of all participants in Extended Data Table 1 and 3.
Reporting on race, ethnicity, or other socially relevant groupings	For this study, we selected participants blind to their age, sex and ethnicity based on the availability of frozen tissue specimens. The demographic characteristics are described in Extended Data Table 1 and 3.
Population characteristics	For this study, we selected participants blind to their age, sex and ethnicity based on the availability of frozen tissue specimens. The demographic characteristics are described in Extended Data Table 1 and 3.
Recruitment	Eyeballs from individuals diagnosed with MS were obtained from the Netherlands Brain Bank, while those from control donors without recognisable neuropathological changes were obtained from Johns Hopkins University. Cortical tissue from individuals diagnosed with MS and control individuals were obtained from the UK Multiple Sclerosis Tissue Bank at Imperial College London.
Ethics oversight	For human eye tissue obtained from the Johns Hopkins University, all procedures were approved within the IRB00374036 “Characterizing mechanisms involved in pathological processes of retina neurodegeneration and optic neuritis present in MS in postmortem samples”. For the human tissue analyses, as the samples could no longer be linked to identifiable individuals, the work did not qualify as a "research project on humans" under § 9 para. 2 of the Hamburg Chamber of Commerce Act for the Health Professions. Consequently, consultation required under § 15 para. 1 of the Professional Code of Conduct for Physicians in Hamburg was not necessary. For cortex tissue obtained from the UK Multiple Sclerosis Tissue Bank at Imperial College London, all procedures used by the Tissue Bank in the procurement, storage and distribution of tissue have been approved by the relevant Multicentre Research Ethics Committee (08/MRE09/31+5).

Note that full information on the approval of the study protocol must also be provided in the manuscript.

Field-specific reporting

Please select the one below that is the best fit for your research. If you are not sure, read the appropriate sections before making your selection.

☒ Life sciences ☐ Behavioural & social sciences ☐ Ecological, evolutionary & environmental sciences

For a reference copy of the document with all sections, see nature.com/documents/nr-reporting-summary-flat.pdf

Life sciences study design

All studies must disclose on these points even when the disclosure is negative.

Sample size	Sample sizes for human single-nucleus RNA sequencing and immunohistochemistry experiments were determined based on sample sizes used in previous studies that successfully identified comparable phenotypic effects in human central nervous system tissue (Lorcom et al., 2026; Lerma-Martin et al., 2024; Absinta et al., 2021; Schirmer et al., 2019). For mouse experiments, sample sizes were selected based on the principles of the 3Rs (Replacement, Reduction and Refinement), the use of genetically homogeneous inbred mouse strains, and sample sizes commonly used in previous studies employing the experimental autoimmune encephalomyelitis (EAE) model that detected comparable biological effects (Woo et al. 2025, Woo et al. 2024, Rothammer et al. 2022, Schattling et al. 2019). A minimum of three independent biological replicates was used for all mouse experiments. No statistical methods were used to predetermine sample size.
Data exclusions	Outlier exclusion was only performed for relative retinal ganglion cell subtype frequencies (Fig. 1h,i). Following inspection of the subtype frequency distributions, one donor sample (Sample 034) appeared different from the remaining cohort. Rather than excluding the sample

based on subjective judgement, Grubbs' test was applied to establish an objective statistical criterion for outlier identification on a per-subtype basis. The use of this criterion was not pre-specified. No more than two samples were excluded for any individual subtype analysis, and inclusion of these samples did not change the statistical significance or the conclusion of this study.

Replication

Flow cytometry gating for enrichment of retinal ganglion cells was applied independently to all biological replicates and yielded consistent cell populations across experiments.
Neuronal CFH upregulation during MS was independently confirmed in a second cohort of cortical tissue from individuals with MS compared to control individuals.
Key findings from snRNA-seq analyses were validated using single-molecule fluorescence in situ hybridization.
To ensure robust transcriptomic comparisons, two different bioinformatic approaches were employed to analyze the differential expression profiles of resilient and susceptible RGCs. Moreover, snRNA-sequencing of a second donor cohort (collected approximately three years after the initial cohort) confirmed susceptible and resilient RGC subtypes. All transcriptomic comparisons were successfully replicated.

To demonstrate that the SCR20 of CFH protects against oxidative stress, different CFH variants lacking the SCR20 were cloned and tested in independent experiments. In addition, three different approaches were used to show that extracellular CFH did not mediate the protective effect (medium exchange, blocking antibody, variants lacking the signal peptide). Experiments involving CFH and its derivatives were repeated with mHDM-FH and derived variants, showing high concordance in effect size.

Randomization

For snRNA-seq experiments, donor allocation to the control or MS group was determined by disease status. To minimize potential batch effects, one control and one MS retina were randomly selected for processing on each experimental day. Samples were processed sequentially, with the order of sample preparation systematically alternated to ensure that the interval between tissue dissociation, cell sorting, and downstream processing was comparable between control and MS samples.
For EAE experiments, if both sexes were included, equal numbers of male and female mice were allocated to each experimental group. Furthermore, animals from each litter were evenly distributed across groups to minimize potential litter effects. Within these constraints, mice were randomly assigned to receive either effector-AAVs or control AAVs.

Blinding

Researchers were not blinded to disease status during FANS of samples for snRNA-seq, since MS and control samples were deliberately alternated during processing to minimize batch effects. Sequencing data analysis was not blinded, as it is a quantitative process that is not subject to human bias. Clinical EAE scoring was conducted in a blinded manner, with researchers unaware of both the genotype and the injected AAV to prevent observer bias. Investigators were also blinded during flow cytometry data acquisition and analysis, as well as during image acquisition and quantitative image analysis.

Reporting for specific materials, systems and methods

We require information from authors about some types of materials, experimental systems and methods used in many studies. Here, indicate whether each material, system or method listed is relevant to your study. If you are not sure if a list item applies to your research, read the appropriate section before selecting a response.

Materials & experimental systems

n/a	Involved in the study	
☐	☒	Antibodies
☐	☒	Eukaryotic cell lines
☒	☐	Palaeontology and archaeology
☐	☒	Animals and other organisms
☒	☐	Clinical data
☒	☐	Dual use research of concern
☒	☐	Plants

Methods

n/a	Involved in the study	
☒	☐	ChIP-seq
☐	☒	Flow cytometry
☒	☐	MRI-based neuroimaging

Antibodies

Antibodies used

Primary Antibodies for Immunoblotting
Target Clone Fluorophore Host Vendor Product # Dilution Isotype Application Validation
anti-GAPDH 14C10 N/A rabbit Cell Signaling 2118 1:1000 IgG WB, IHC,IF, FC, https://www.cellsignal.com/products/primary-antibodies/gapdh-14c10-rabbit-monoclonal-antibody/2118?srsltid=AfmBOor3SFcGheNazHt6BayEqG-J-rbdZQZYohdT-2topJojDMl5ckqk
anti-C3/C3b/C3c polyclonal N/A rabbit Proteintech 21337-1-AP 1:1000 IgG WB, IP, IHC, IF, https://www.ptglab.com/de/products/C3-Antibody-21337-1-AP.htm?srsltid=AfmBOoqI5UtVepig7ugi6nFcrR9smthSYESW-Lqf4u7Wmcnny61hw6yQ
anti-Factor B polyclonal N/A goat Quidel A311 1:1000 IgG WB, IHC, IP, IF, https://www.quidelortho.com/us/en/products/microvue-assays/factor-b-antibody-polyclonal
anti-CFH polyclonal N/A sheep R&D Systems AF4999 1:200 IgG WB, https://www.rndsystems.com/products/mouse-complement-factor-h-antibody_af4999
anti-Calnexin polyclonal N/A rabbit Enzo ADI-SPA-860-D 1:1000 IgG ICC, IF, IHC (PS), IP, WB, https://www.enzo.com/product/calnexin-polyclonal-antibody/
anti-Actin polyclonal N/A rabbit Cell Signaling 4967 1:1000 IgG WB, https://www.cellsignal.com/products/primary-antibodies/beta-actin-antibody/4967?srsltid=AfmBOoqkiGgl1ubLA-gIX6M0eTSV2KwAhGEEGX0Xz6zrYusfQSjIUrOf

Primary Antibodies for IF and IHC staining
Target Clone Fluorophore Host Vendor Product # Dilution Isotype Application Validation

anti-NeuN polyclonal N/A guinea pig Synaptic Systems 266 004 1:500 antiserum IHC, ICC, IHC-P, IHC-Fr, https://www.sysy.com/product/1459
anti-NeuN polyclonal N/A chicken Merck Millipore ABN91 1:500 IgY WB, ICC, IHC, https://www.merckmillipore.com/DE/de/product/Anti-NeuN-Antibody,MM_NF-ABN91
anti-RBPMS polyclonal N/A rabbit abcam ab194213 1:500 IgG WB, IHC, https://www.abcam.com/en-us/products/primary-antibodies/rbpms-antibody-ab194213, PMID: 40806966, 40345829, 40126507
anti-CFH polyclonal N/A goat Quidel A312 1:200 IgG WB, ELISA, IF, https://www.quidelortho.com/us/en/products/microvue-assays/factor-h-antibody-polyclonal
anti-CFH polyclonal N/A sheep R&D Systems AF4999 1:200 IgG WB, https://www.rndsystems.com/products/mouse-complement-factor-h-antibody_af4999, this paper Extended Data Fig. 2
anti-CFH polyclonal N/A goat R&D Systems AF4779 1:40 IgG WB, IHC, https://www.rndsystems.com/products/human-complement-factor-h-antibody_af4779
anti-MDA polyclonal N/A rabbit abcam ab27642 1:200 IgG ELISA, WB, IF, https://www.abcam.com/en-us/products/primary-antibodies/malondialdehyde-antibody-ab27642, PMID 33106352, 39481440, 34262021
anti-GFP polyclonal N/A chicken abcam ab13970 1:1000 IgY WB, ICC, IF, https://www.abcam.com/en-us/products/primary-antibodies/gfp-antibody-ab13970
anti-RFP Ch337B1 N/A chicken Synaptic Systems 409 009 1:500 IgY (λ light chain) ICC, IHC, https://www.sysy.com/product/409009
anti-SMI 312 SMI 312 N/A mouse BioLegend 837904 1:500 Mouse IgG1/Mouse IgM IHC-P - Quality tested, WB - Verified, EM, ICC, IHC-F, https://www.biolegend.com/de-de/products/purified-anti-neurofilament-marker-pan-axonal-cocktail-12811, PMID 39145444
anti-COX2 polyclonal N/A rabbit abcam ab15191 1:200 IgG WB, ELISA, IHC-P, https://www.abcam.com/en-us/products/primary-antibodies/cox2-cyclooxygenase-2-antibody-ab15191
anti-iNOS SP126 N/A rabbit abcam ab115819 1:200 IgG EM, ICC, https://www.abcam.com/en-us/products/primary-antibodies/inos-antibody-sp126-ab115819, PMID 40532699
anti-C3 11H9 N/A rat abcam ab11862 1:500 IgG2a ICC,IF, https://www.abcam.com/en-us/products/primary-antibodies/c3-antibody-11h9-ab11862

Antibodies for immune cell FACS
Target Clone Fluorophore Host Vendor Product # Dilution Isotype Application validation
Anti-ACSA-2 IH3-18A3 APC Rat Miltenyi Biotec 130-117-386 1:50 IgG2b FC, MICS, IF, IHC, https://www.miltenyibiotec.com/DE-en/products/acsa-2-antibody-anti-mouse-ih3-18a3.html#conjugate=pe:size=30-ug-in-200-ul
Anti-CD4 OKT4 BV711 Rat BioLegend 100447 1:200 IgG2b FC - Quality tested, IHC-F - Verified, https://www.biolegend.com/de-de/products/brilliant-violet-711-anti-mouse-cd4-antibody-10706
Anti-CD8α 53-6.7 BV786 Rat BioLegend 100750 1:200 IgG2a FC (Routinely Tested), https://www.biolegend.com/de-de/products/brilliant-violet-785-anti-mouse-cd8a-antibody-7957
Anti-CD11b M1/70 BUV395 Rat BD Biosciences 563553 1:400 IgG2b FC (Routinely Tested), https://www.miltenyibiotec.com/DE-en/products/cd11b-antibody-anti-human-reafinity-rea713.html#conjugate=viogreen:size=100-tests-in-200-ul
Anti-CD11c N418 PE/Cy7 Armenian Hamster BioLegend 117318 1:400 IgG FC - Quality tested, https://www.biolegend.com/de-de/products/pe-cyanine7-anti-mouse-cd11c-antibody-3086
Anti-CD19 1D3 BUV661 Rat BD Biosciences 612971 1:400 IgG2a FC (Routinely Tested), https://www.bdbiosciences.com/en-eu/products/reagents/flow-cytometry-reagents/research-reagents/single-color-antibodies-ruo/buv661-rat-anti-mouse-cd19.612971?tab=product_details
Anti-CD45 30-F11 APC/Cy7 Rat BioLegend 103117 1:200 IgG2b FC - Quality tested, https://www.biolegend.com/de-de/products/apc-cyanine7-anti-mouse-cd45-antibody-2530
Anti-CD68 FA-11 PE/Cy7 Rat BioLegend 137028 1:200 IgG2a ICFC - Quality tested, FC - Verified, https://www.biolegend.com/en-dk/products/pe-cyanine7-anti-mouse-cd68-antibody-9124
Anti-CD80 16-10A1 BV605 Armenian Hamster BioLegend 104729 1:100 IgG FC - Quality tested, https://www.biolegend.com/de-de/products/brilliant-violet-605-anti-mouse-cd80-antibody-7641
Anti-CD86 PO3 BUV496 Rat BD Biosciences 750437 1:200 IgG2b FC (Qualified), https://www.bdbiosciences.com/en-se/products/reagents/flow-cytometry-reagents/research-reagents/single-color-antibodies-ruo/buv496-rat-anti-mouse-cd86.750437?tab=product_details
Anti-CD206 C068C2 BV711 Rat BioLegend 141729 1:50 IgG2a ICFC - Quality tested, FC - Verified, https://www.biolegend.com/de-de/products/brilliant-violet-711-anti-mouse-cd206-mmr-antibody-12012
Anti-CX3CR1 SA011F11 PB Mouse BioLegend 149038 1:200 IgG2a FC - Quality tested, https://www.biolegend.com/de-de/products/pacific-blue-anti-mouse-cx3cr1-antibody-13857
Anti-F4/80 BM8 BV421 Rat BioLegend 123137 1:100 IgG2a FC - Quality tested, https://www.biolegend.com/de-de/products/brilliant-violet-421-anti-mouse-f4-80-antibody-7199
Anti-MHC II (I-A/I-E) M5/114.15.2 BUV805 Rat BD Biosciences 748844 1:200 IgG2b Flow cytometry (Qualified), https://www.bdbiosciences.com/en-dk/products/reagents/flow-cytometry-reagents/research-reagents/single-color-antibodies-ruo/buv805-rat-anti-mouse-i-a-i-e.748844?tab=product_details
Anti-Ly6C HK1.4 PE Rat BioLegend 128008 1:200 IgG2c FC - Quality tested, https://www.biolegend.com/de-de/products/pe-anti-mouse-ly-6c-antibody-4904?GroupID=BLG5853
Anti-Ly6G 1A8 PerCP/Cy5.5 Rat BioLegend 127616 1:100 IgG2b FC - Quality tested, https://www.biolegend.com/en-gb/products/percp-cyanine5-5-anti-mouse-ly-6g-antibody-6116?GroupID=BLG7234
Anti-P2RY12 S16007D PE Rat BioLegend 848004 1:100 IgG2b FC - Quality tested, https://www.biolegend.com/de-de/products/pe-anti-mouse-p2ry12-antibody-15400
Anti-TCRβ H57-597 BUV737 Armenian Hamster BD Biosciences 612821 1:100 IgG2 FC (Routinely Tested), https://www.bdbiosciences.com/en-us/products/reagents/flow-cytometry-reagents/research-reagents/single-color-antibodies/buv737-hamster-anti-mouse-tcr-chain.612821?tab=product_details
Anti-TREM2 237920 APC Rat R&D Systems FAB17291A 1:10 IgG2b N/A, https://www.rndsystems.com/products/human-mouse-trem2-apc-conjugated-antibody-237920_fab17291a
Anti-CD44 IM7 APC/Cy7 Rat BioLegend 103028 1:200 IgG2b, https://www.biolegend.com/de-de/products/apc-cyanine7-anti-mouse-human-cd44-antibody-3933
Anti-CD192 Y15-488 rMAb PE Rat BD Biosciences 568093 1:50 IgG1, https://www.bdbiosciences.com/en-br/products/reagents/flow-cytometry-reagents/research-reagents/single-color-antibodies-ruo/pe-rat-anti-mouse-cd192-ccr2.568093?tab=product_details
Anti-CX3CR1 SA011F11 FITC Mouse BioLegend 149020 1:200 IgG2a - Quality tested, https://www.biolegend.com/de-de/products/fitc-anti-mouse-cx3cr1-antibody-11878

Anti-Ly6C HK1.4 BV785 Rat BioLegend 128041 1:100 IgG2a, https://www.biolegend.com/en-us/products/brilliant-violet-785-anti-mouse-ly-6c-antibody-11982

Primary and secondary antibodies for sorting of nuclei
Target Clone Fluorophore Host Vendor Product # Dilution Isotype Application Validation
anti-NeuN polyclonal N/A Chicken Merck Millipore ABN91 1:250 IgY WB, ICC, IHC, https://www.merckmillipore.com/DE/de/product/Anti-NeuN-Antibody,MM_NF-ABN91, PMID 35930635, 33661276, 40532699
anti-RBPMS polyclonal N/A Rabbit abcam ab194213 1:250 IgG IHC, F, https://www.abcam.com/en-us/products/primary-antibodies/rbpms-antibody-ab194213, this paper Figure 1, Extended Data Figure 1

Neutralizing Antibodies
Target Clone Fluorophore Host Vendor Product # Dilution Isotype Application Validation
CFH HM2248 N/A mouse Hycult HM2248 1:100 IgG1 Immuno assays, WB, https://www.hycultbiotech.com/product/complement-factor-h-human-mab-c18-3/, PMID 17495132

Secondary antibodies for sorting of nuclei
anti-rabbit PE polyclonal PE Donkey Biolegend Poly4064 1:200 FC - Quality tested
anti-chicken IgG H&L polyclonal AF647 Donkey Jackson Immuno Research 703-605-155 1:500 IgG ICC, IHC

Secondary Antibodies IF/IHC stainings
Target Clone Fluorophore Host Vendor Product # Reference Isotype Application Validation
anti-rabbit IgG H&L polyclonal AF488 Donkey abcam ab181346 1:500 IgG ELISA, IHC-Fr, IHC-P, FC, ICC/IF
anti-mouse IgG polyclonal AF488 Donkey abcam ab150105 1:500 IgG ELISA, IHC-Fr, IHC-P, FC, ICC/IF
anti-chicken IgG polyclonal AF488 Donkey Jackson Immuno Research 703-545-155 1:500 IgG ICC, IHC
anti-rabbit IgG polyclonal AF555 Donkey abcam ab150074 1:500 IgG ELISA, IHC-Fr, IHC-P, FC, ICC/IF
anti-mouse IgG polyclonal AF555 Donkey abcam ab150106 1:500 IgG ELISA, IHC-Fr, IHC-P, FC, ICC/IF
anti-guinea pig IgG polyclonal Cy3 Donkey Jackson Immuno Research 706-166-148 1:500 IgG ICC, IHC
anti-chicken IgG polyclonal AF555 Donkey Jackson Immuno Research 703-165-155 1:500 IgG ICC, IHC
anti-rabbit IgG H&L polyclonal AF647 Donkey abcam ab181347 1:500 IgG ELISA, IHC-Fr, IHC-P, FC, ICC/IF
anti-mouse IgG H&L polyclonal AF647 Donkey abcam ab150111 1:500 IgG ELISA, IHC-Fr, IHC-P, FC, ICC/IF
anti-sheep IgG H&L polyclonal AF647 Donkey abcam ab150179 1:500 IgG ELISA, IHC-Fr, IHC-P, ICC/IF, FC
anti-chicken IgG H&L polyclonal AF647 Donkey Jackson Immuno Research 703-605-155 1:500 IgG ICC, IHC

Secondary Antibodies for Immunblotting
Target Clone Fluorophore Host Vendor Product # Dilution Isotype Application Validation
anti-rabbit-HRP polyclonal N/A Goat LICORbio 926-80011 1:10000 IgG WB
anti-goat-HRP polyclonal N/A Donkey ThermoFisher A15999 1:10000 IgG WB, IHC, ELISA
anti-sheep-HRP polyclonal N/A Donkey ThermoFisher A16041 1:10000 IgG WB, IHC, ELISA

Validation

All antibodies were purchased from commercial vendors. Antibodies have been validated for use by the manufacturers as described on their respective website and listed above as well as in Extended Data Table 3. Additionally, in this study, knockout validation was performed for the anti-CFH antibody (#AF4999 from R&D).

Eukaryotic cell lines

Policy information about cell lines and Sex and Gender in Research

Cell line source(s)

Immortalized mouse embryonic fibroblasts (Pfa1) were received from the Conrad laboratory (Helmholtz Institute) and have been described in detail elsewhere (PMID: 18762024, 35922516). HEK 293T (ACC 635, DMSZ, purchased in 2017)

Authentication

Pfa1 cells were obtained from the originating laboratory and were not further authenticated. HEK 293T cells were purchased from an authorised vendor.

Mycoplasma contamination

All cell lines were regularly checked for mycoplasma contamination using the VenorGeM Advance kit (Minerva biolabs, 11-7024) according to the manufacturer's instructions. Cell lines were free of mycoplasma contamination.

Commonly misidentified lines
(See ICLAC register)

None of the cell lines used in this study is listed in the registry.

Animals and other research organisms

Policy information about studies involving animals; ARRIVE guidelines recommended for reporting animal research, and Sex and Gender in Research

Laboratory animals

All mice, including C57BL/6J WT (The Jackson Laboratory), SJL/JRj WT (Janvier Labs), C3 knockout (B6.129S4-C3tm1Crr/J, The Jackson Laboratory) and Cfh-flox mice were maintained under specific pathogen-free conditions in the central animal facility of the University Medical Center Hamburg-Eppendorf (UKE). Adult mice aged 8–20 weeks were used for all experiments. The mice were kept in a 12-hour light/12 hour dark cycle, at 22 ± 2°C and 40-60% relative humidity with ad libitum access to standard chow (Altromin, 1328P) and water. EAE mice additionally received DietGel® Recovery (Ssniff, H007-72065).
For EAE experiments involving C57BL/6J WT, SJL/JRj WT and C3 knockout mice, only female animals were included. To assess potential sex dependent effects, an additional cohort of male C57BL/6J mice was used to compare neuronal mHDM-FH overexpression with a GFP-only control. For experiments with Cfh-flox mice both sexes were used. As no sex-specific differences were observed, data from both sexes were pooled and reported together.

Generation of CFH neuronal knockout mice: CFHtm1a(EUCOMM) Wtsi sperm was ordered (the Jackson Laboratory) and revitalised on C57/B6 wildtype animals (The Jackson Laboratory), resulting in litters positive for the CFHtm1a(EUCOMM) Wtsi allele. To obtain the conditional (tm1c) allele (Cfh-flox), animals were bred with ACTB:FLPe B6J mice expressing the Flip recombinase (The Jackson laboratory, Strain #005703)73. To generate neuronal CFH knockouts we performed intravenous injections of an AAV (serotype PhP.eB) carrying a Cre recombinase under the control of an hSyn1 promotor.

Wild animals

This study does not involve wild animals.

Reporting on sex

For EAE experiments involving C57BL/6J WT, SJL/JRj WT and C3 knockout mice, only female animals were included. To assess potential sex dependent effects, an additional cohort of male C57BL/6J mice was used to compare neuronal mHDM-FH overexpression with a GFP-only control. For experiments with Cfh-flox mice both sexes were used. As no sex-specific differences were observed, data from both sexes were pooled and reported together.

Field-collected samples

This study does not include field-collected samples.

Ethics oversight

All experimental procedures involving animals complied with institutional guidelines and adhered to the German Animal Welfare Act. Ethical approval for animal experiments was granted by the State Authority of Hamburg, Germany (approval numbers 41/22, 53/24, 40/25).

Note that full information on the approval of the study protocol must also be provided in the manuscript.

Plants

Seed stocks

No plants were uses in this study

Novel plant genotypes

Describe the methods by which all novel plant genotypes were produced. This includes those generated by transgenic approaches, gene editing, chemical/radiation-based mutagenesis and hybridization. For transgenic lines, describe the transformation method, the number of independent lines analyzed and the generation upon which experiments were performed. For gene-edited lines, describe the editor used, the endogenous sequence targeted for editing, the targeting guide RNA sequence (if applicable) and how the editor was applied.

Authentication

Describe any authentication procedures for each seed stock used or novel genotype generated. Describe any experiments used to assess the effect of a mutation and, where applicable, how potential secondary effects (e.g. second site T-DNA insertions, mosiacism, off-target gene editing) were examined.

Flow Cytometry

Plots

Confirm that:

☒ The axis labels state the marker and fluorochrome used (e.g. CD4-FITC).

☒ The axis scales are clearly visible. Include numbers along axes only for bottom left plot of group (a 'group' is an analysis of identical markers).

☒ All plots are contour plots with outliers or pseudocolor plots.

☒ A numerical value for number of cells or percentage (with statistics) is provided.

Methodology

Sample preparation

For immune cell characterization in EAE:
Spinal cord tissue was collected after transcardial PBS perfusion and dissociated into single-cell suspensions using 1 mg mL–1 collagenase A (Roche, 11088793001) and 0.1 mg mL–1 DNase I from bovine pancreas (Merck Millipore, Cat. No. 260913) with the gentleMACS Octo Dissociator (Miltenyi Biotec, program: Multi_F). The dissociated tissue was passed through a 70 µm cell strainer, and immune and glia cells were enriched using a discontinuous density gradient. After centrifugation at 2,500 rpm, 4 °C for 30 minutes, cells were collected from the interphase between the 30% Percoll and 70% Percoll layers. Non-specific Fc receptor–mediated antibody binding was blocked by pre-incubation with TruStain FcX anti-mouse CD16/32 antibody (BioLegend, Cat. No. 101320) for 10 minutes at 4 °C, followed by surface antibody staining in Brilliant Stain Buffer (BD Biosciences, Cat. No. 563794) for 30 minutes at 4 °C. Dead cells were excluded from analysis using Zombie Green Fixable Viability Stain (BioLegend, Cat. No. 423112). For intracellular staining of CD206 and CD68, cell suspensions were fixed for 20 minutes at room temperature using Fixation Buffer (BioLegend, Cat. No. 420801), followed by 20 minutes incubation with anti-CD68 antibody in Intracellular Staining Permeabilization Wash Buffer (BioLegend, Cat. No. 421002). Absolute cell numbers for CD45high leukocytes and CD45med microglia were determined using Precision Count Beads (BioLegend, Cat. No. 424902).
For enrichment of retinal ganglion cell nuclei: Postmortem fresh frozen retinal tissue was homogenized in Tris-based lysis buffer containing 0.1% NP40 to extract nuclei. Nuclei were filtered and pelleted at 500g for 5 minutes followed by incubation with antibodies against NeuN and RBPMS for 15 minutes in Tris-based staining buffer containing 0.02% Tween and 2% BSA. Nuclei were washed and pelleted at 500g for 5 min and resuspended in staining buffer with secondary antibody followed by incubation for 15 minutes. Following a final washing step, nuclei were resuspended in sorting buffer (Tris-based buffer with 2% BSA) and collected in Ames medium supplemented with 1.5% BSA.

Instrument	For immune cell characterization in EAE: BD Symphony A3 flow cytometer (BD Biosciences)<br>For enrichment of retinal ganglion cell nuclei for snRNA-seq: BD FACS Aria III Cell Sorter
Software	For immune cell characterization in EAE: FlowJo v10.9 (BD Biosciences)<br>For enrichment of retinal ganglion cell nuclei for snRNA-seq: BD FACSDiva v9.0.1 (BD Biosciences)
Cell population abundance	For enrichment of retinal ganglion cells: nuclei were sorted based on their positivity for both NeuN and RBPMS (1-5% of total events). For immune cell characterization in EAE, the abundance of cell populations is provided in the respective figures.
Gating strategy	For enrichment of retinal ganglion cell nuclei: A FSC-A and SSC-A gate was used to exclude debris. A FSC-A/ FSC-H gate was used to extract single nuclei. A DAPI/FSC-A gate selected for DAPI positive single nuclei. NeuN+/RBPMS+ nuclei were selected to enrich for retinal ganglion cells.<br>The gating strategies for immune cell characterization in EAE are provided in Extended Data Fig. 7, 9 and 24.

☒ Tick this box to confirm that a figure exemplifying the gating strategy is provided in the Supplementary Information.
